# Genetic Diversity and Selection in Three *Plasmodium vivax* Merozoite Surface Protein 7 (*Pvmsp-7*) Genes in a Colombian Population

**DOI:** 10.1371/journal.pone.0045962

**Published:** 2012-09-25

**Authors:** Diego Garzón-Ospina, Carolina López, Johanna Forero-Rodríguez, Manuel A. Patarroyo

**Affiliations:** 1 Fundación Instituto de Inmunología de Colombia – FIDIC, Bogotá DC, Colombia; 2 Microbiology postgraduate program, Universidad Nacional de Colombia, Bogotá DC, Colombia; 3 School of Medicine and Health Sciences, Universidad del Rosario, Bogotá DC, Colombia; Universidade Federal de Minas Gerais, Brazil

## Abstract

A completely effective vaccine for malaria (one of the major infectious diseases worldwide) is not yet available; different membrane proteins involved in parasite-host interactions have been proposed as candidates for designing it. It has been found that proteins encoded by the merozoite surface protein (*msp*)-7 multigene family are antibody targets in natural infection; the nucleotide diversity of three *Pvmsp-7* genes was thus analyzed in a Colombian parasite population. By contrast with *P. falciparum msp-7* loci and ancestral *P. vivax msp-7* genes, specie-specific duplicates of the latter specie display high genetic variability, generated by single nucleotide polymorphisms, repeat regions, and recombination. At least three major allele types are present in *Pvmsp-7C*, *Pvmsp-7H* and *Pvmsp-7I* and positive selection seems to be operating on the central region of these *msp-7* genes. Although this region has high genetic polymorphism, the C-terminus (Pfam domain ID: PF12948) is conserved and could be an important candidate when designing a subunit-based antimalarial vaccine.

## Introduction

*Plasmodium vivax* malaria should not be considered a benign disease anymore due to its wide geographical distribution [1], [2] and ability to cause severe clinical syndromes [3], [4], [5], [6]. It was estimated that 2.85 billion people were at risk of being infected by this parasite in 2009 [1]. Although several research groups have focused on developing a vaccine against *P. vivax*, relatively few vaccine candidate molecules have been thoroughly characterized [7], [8]; some of them are membrane proteins making attractive targets for vaccine development since they are essential for the recognition and invasion of erythrocytes, hence immune responses directed against them could inhibit parasite entry [9]. The merozoite surface protein (MSP) family is a group of surface proteins which are involved in the initial interaction between the parasite and the host cell. However, the genetic diversity of these proteins has been the main problem for developing a vaccine [10], [11], [12], [13], [14]. Proteins displaying high antigenic diversity elicit strain-specific immune responses leading to low protective efficacy upon vaccination, while proteins displaying limited variability are attractive targets for testing as vaccine candidates. Research involving proteins displaying high overall polymorphism should be focused on the conserved functional domains [15] so that strain-specific responses may be avoided.

MSP-1, MSP-6 and MSP-7 form the most abundant protein complex on the *P. falciparum* merozoite surface [16], [17]. *msp-1* and *msp-6* are both single copy genes while *msp-7* has a large number of genes which seem not to have diverged functionally. Furthermore, the *Plasmodium* genus has dissimilar copy numbers of this gene [18], [19]. The *msp-7* genes have a single exon and have been named in alphabetical order according to their position regarding the PVX_082640 flanking gene [18]. Different *msp-7* genes are expressed simultaneously in the schizont stage of *Plasmodium* species [20], [21], [22] and several of them have been localized on the membrane surface [19], [21], [22], [23], not only forming part of the main merozoite protein complex but also binding to erythrocytes [24]. Knockout and invasion inhibition assays have also shown that MSP-7 is involved in the invasion of erythrocytes [19], [24], [25]. Moreover, Wang’s group has shown that a recombinant MSP-7 is recognized by sera from malaria-infected individuals where IgG3 subclass antibodies prevail [26]. Immunization with members of this family has been shown to protect vaccinated mice against experimental challenge [21].

*P. falciparum* has eight *msp-7* genes and *P. vivax* eleven, [18], [27]; the members of this family have low genetic variability in the former specie, [28], [29] as do the *P. vivax msp-7A* (GenBank ID: XM_001614080.1) and *msp-7K* (GenBank ID: XM_001614090.1) genes [30]. A recent study has shown that *P. vivax* specie-specific duplicates MSP-7C (GenBank ID: XM_001614082.1) and MSP-7H (GenBank ID: XM_001614087.1) are recognized by IgG antibodies from *P. vivax*-infected patient sera [31]. These proteins are phylogenetically related to PvMSP-7I (GenBank ID: XM_001614088.1) [18], [27] and fragments from different MSP-7 proteins may have to be included to block this invasion route due to the biological implication that functional redundancy might have on vaccine development. Taking into account that some of these proteins are recognized by the immune system, their genetic diversity should be evaluated to determine their potential use as potent anti-malarial vaccine candidates.

## Results

Forty-eight parasite samples were collected from symptomatic patients living in representative regions of Colombia for this study: Amazonian (Calamar, Guaviare department), Andean (Apartadó and El Bagre, Antioquia department), Caribbean (Tierra Alta and Puerto Libertador, Córdoba department), Orinoquia (Mapiripan, Meta department and Tauramena, Casanare department) and Pacific (Istmina, Chocó department and Tumaco, Nariño department) (Fig. S1). Forty-two samples corresponding to single *P. vivax msp-1* allele infections were considered for PCR amplification even though amplicons were not detected in a few samples (*Pvmsp-7C* n = 37, *Pvmsp-7H* n = 37 and *Pvmsp-7I* n = 42).

### Polymorphism in *Pvmsp-7* Loci

1,167-bp *Pvmsp-7C*, 1,232-bp *Pvmsp-7H* and 1,182-bp *Pvmsp-7I* gene fragments were amplified and direct sequenced. Nucleotide sequence data here reported are available in GenBank: accession numbers JQ423957-JQ424037. Twenty-three haplotypes were found for *Pvmsp-7C* while twenty-eight haplotypes were found for both *Pvmsp-*7*H* and *Pvmsp-*7*I*. Haplotype diversity (Hd) was lower in the *Pvmsp-7C* gene than in *Pvmsp-7H* and *Pvmsp-*7*I* (Table 1). 148 sites from the total nucleotide sequence length analyzed were polymorphic in *Pvmsp-7C* and 142 sites in *Pvmsp-7H,* while 121 polymorphic sites were found in *Pvmsp-7I* (Table 1).

**Table 1:** Estimates of DNA diversity and neutrality test for *Pvmsp-7* genes from a Colombian population.

n	Gene	Sites	Ss	S	Ps	H	Hd (se)	θ^w^ (se)	π (se)	Tajima	Fu & Li		Fu	Z_ns_	ZZ	RM
										D	D*	F*	Fs			
37	*msp-7C*	1,098	148	1	147	23	0.93 (0.03)	0.0323 (0.001)	0.0548 (0.003)	2.094**	1.810*	2.287*	7.64	0.339	0.173*	22
37	*msp-7H*	1,131	142	19	123	28	0.96 (0.02)	0.0301 (0.002)	0.0357 (0.003)	0.388	0.952	0.987	-0.57	0.126	0.343*	27
42	*msp-7I*	1,058	127	6	121	28	0.97 (0.01)	0.0280 (0.002)	0.0430 (0.003)	1.420	1.584**	1.818*	2.12	0.232	0.334*	13

Ss: Number of segregating sites, S: number of singleton sites, Ps: number of parsimony-informative sites, H: number of haplotypes, Hd: haplotype diversity, θ^W^: Watterson estimator, π: nucleotide diversity. (se): Standard deviation. Sites excluded from the analysis: *Pvmsp-7C*: nucleotides 1 to 17 (amino acids 1 to 6), 544 to 546 (amino acid 182), 616 to 618 (amino acid 206), 625 to 627 (amino acid 209), 631 to 648 (amino acids 211 to 216), 655 to 666 (amino acids 219 to 222), 676 to 684 (amino acids 226 to 228), 706 to 708 (amino acid 236), 1,105 to 1,107 (amino acid 369) and nucleotides 1,168 to 1,191 (amino acids 390 to 397); *Pvmsp-7H*: nucleotides 436 to 450 (amino acids 146 to 150), 448 to 486 (amino acid 162), 568 to 627 (amino acids 190 to 200) and nucleotides 772 to 774 (amino acid 258); *Pvmsp-7I*: nucleotides 1 to 18 (amino acids 1 to 6), 421 to 522 (amino acids 141 to 174) and nucleotides 526 to 537 (amino acids 176 to 179).

* : p<0.02, **: p<0.05.

Nucleotide diversity for these three genes (*Pvmsp-7C* π = 0.0548, *Pvmsp-7H* π = 0.0357 and *Pvmsp-7I* π = 0.0430) was higher than that previously reported for other *P. vivax msp-7* family members (*Pvmsp-7A* π = 0 and *Pvmsp-7K* π = 0.0022) [30]. Furthermore, the *msp-7* genes from this study were among the most polymorphic *P. vivax* genes reported to date (Table S1). Regarding *Pvmsp-7* diversity (π value average) in the different Colombian regions, the Pacific area was the most diverse followed by the Andean, Caribbean and Orinoco regions while the Amazonian was the least polymorphic (Table S2).

**Figure 1:**
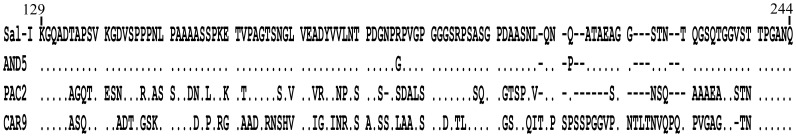
Alignment of deduced PvMSP-7C amino acid sequences, including the Sal-I sequence in which the three main allele types can be observed. Dots indicate conserved residues and dashes represent gaps introduced for alignment.

Three major allele types were found in deduced PvMSP-7C amino acid sequences (Fig. 1 and Fig. S2) between residues 134 and 238 (numbered according to the alignment in Fig. S2). Allele types had similar frequency in the Colombian parasite population.

**Figure 2:**
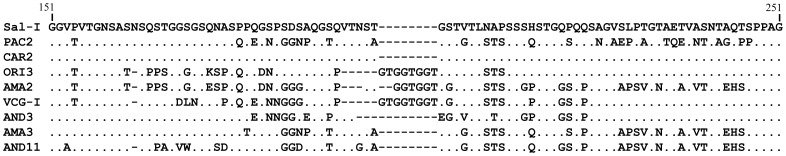
Alignment of deduced PvMSP-7H amino acid sequences, including the Sal-I sequence. The alignment shows major allele types found in Colombian populations. Dots indicate conserved residues and dashes represent gaps introduced for alignment.

An AEAFG insertion-deletion from residue 146 to 150 and a GTG_(n)_GT[V/E] repeat region from residue 195 to 209 (numbered according to the alignment in Fig. S3) were revealed by protein sequence alignment in PvMSP-7H. Three regions throughout the protein sequences led to discriminating several allele types (Fig. 2 and Fig. S3). The first region from residue 153 to 170 (numbered according to the alignment given in the Fig. S3) had four different peptide sequences. Five different sequences were found in the second region (from residue 172 to 194) and three different ones were found from residue 229 to 247. These three regions were randomly associated (Fig. 2 and Fig. S3).

**Figure 3:**
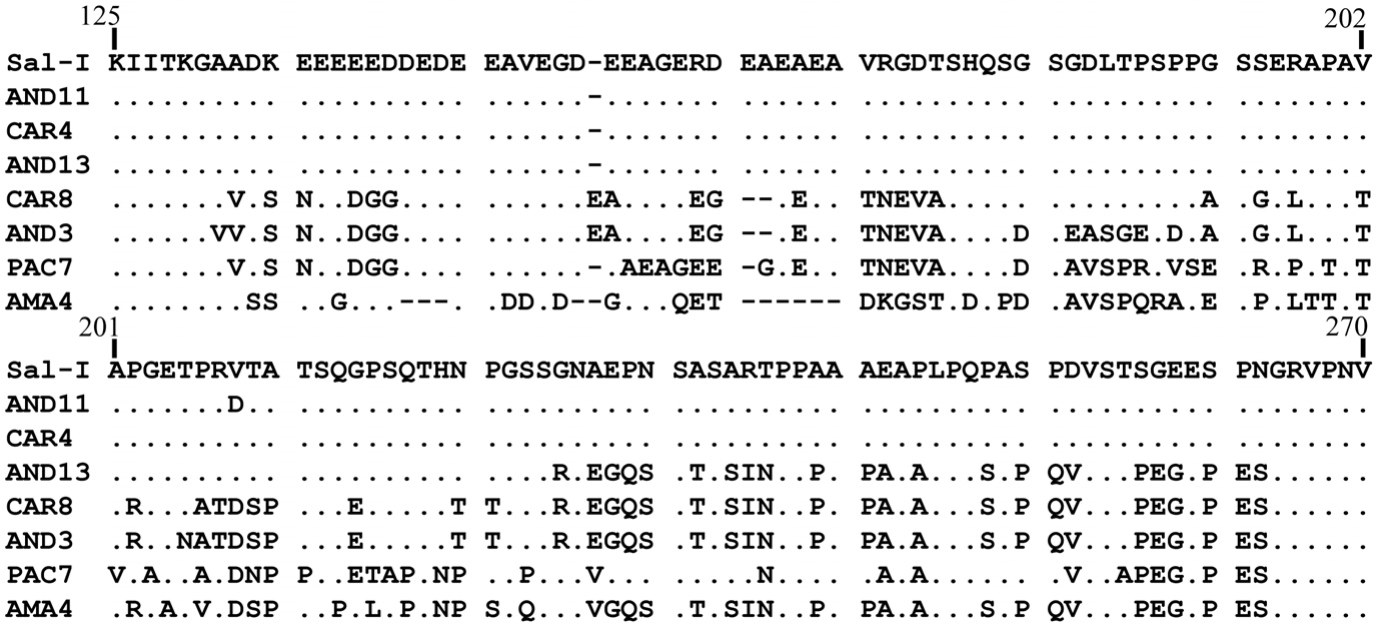
Alignment of deduced PvMSP-7I amino acid sequences, including the Sal-I sequence in which the protein sequences differentiating major allele types can be observed. Dots indicate conserved residues and dashes represent gaps introduced for alignment.

EEAVEGD and EA repeats could be observed in the deduced PvMSP-7I amino acid sequences. Similar to the genes mentioned above, several major allele types were observed between residues 131 and 219 (numbered according to the alignment in Fig. S4) characterized by two regions. The first region (between amino acids 131 and 140) had three different peptide sequences (Fig. 3 and Fig. S4). The second region ran from residue 163 to residue 219, having an extra four different peptide sequences. These regions were not randomly associated (Fig. 3 and Fig. S4). A further four peptide sequences were found from residue 221 to residue 234 and two more from residue 236 to residue 264 and, contrary to those mentioned above, these fragments were found to be associated with either of the alleles described above (Fig. 3 and Fig. S4).

### Neutral Evolutionary Test and Selection in the *Pvmsp-7* Loci

Applying neutral evolution tests (Tajima, Fu & Li) to the Colombian *P. vivax* population gave significant values above 0 for *Pvmsp-7C* and *Pvmsp-7I* (p<0.05 Tajima D, p<0.02 Fu & Li D* and F*) (Table 1); these values indicated an excess of intermediate frequency alleles. The neutral evolution tests for *Pvmsp-7H* revealed no statistically significant differences; this gene therefore seems to be evolving under neutral expectations. However, a sliding window analysis for D, D* and F* statistics showed that different selective forces could be acting throughout the *Pvmsp-7* genes. It was found that balancing selection acted on the central gene region while negative selection could be acting at the 5′-ends (significant values for *Pvmsp-7C* and *Pvmsp-7H*) and 3′-ends (no significant values) (Fig. S5).

The average number of synonymous substitutions per synonymous site (d_S_) and non-synonymous substitutions per non-synonymous site (d_N_) was estimated to determine whether natural selection was affecting the *msp-7* loci. Although d_N_ was higher than d_S_ in *Pvmsp-7C* and *Pvmsp-7I*, d_N_ was lower than d_S_ in the *Pvmsp-7H* gene even though these values were not statistically significant (Table 2). Taking into account that a sliding window for ω (d_N_/d_S_) (Fig. S6) showed that the ω rate was higher than 1 (signal of positive selection) in the central region of the *msp-7* genes analyzed here, the genes were thus split into 3 regions: 5′-end (*msp-7C*: between nucleotides 1 and 390, *msp-7H*: between nucleotides 1 and 471, *msp-7I*: between nucleotides 1 and 525), central (*msp-7C*: between nucleotides 391 and 717, *msp-7H*: between nucleotides 472 and 771, *msp-7I*: between nucleotides 526 and 789) and 3′-end (*msp-7C*: between nucleotide 718 and 1,191, *msp-7H*: between nucleotide 772 and 1,200, *msp-7I*: between nucleotide 790 and 1,188) (Table S3); d_N_ and d_S_ were then computed. d_S_ was higher than d_N_ for both 5′- and 3′-ends, just the 5′-end having significant statistical difference. On the other hand, the d_N_ substitutions in the central region were significantly higher than d_S_ substitutions for the three genes (Table 2).

**Table 2:** Average number of synonymous substitutions per synonymous site (d_S_) and non-synonymous substitutions per non-synonymous site (d_N_) covering all sequence pairs at the 5′-end, central region, 3′-end and complete gene.

	5′-end		Central region		3′-end		Full length gene	
	d_S_ (se)	d_N_ (se)	d_S_ (se)	d_N_ (se)	d_S_ (se)	d_N_ (se)	d_S_ (se)	d_N_ (se)
*msp-7C*	0.070 (0.016)**	0.011 (0.004)	0.122 (0.023)	0.287 (0.031)•	0.004 (0.003)	0.000 (0.000)	0.053 (0.008)	0.059 (0.007)
*msp-7H*	0.059 (0.010)•	0.010 (0.003)	0.052 (0.016)	0.162 (0.019)•	0.010 (0.005)	0.005 (0.002)	0.038 (0.006)	0.037 (0.005)
*msp-7I*	0.041 (0.014)*	0.020 (0.007)	0.060 (0.019)	0.186 (0.021)•	0.017 (0.007)	0.007 (0.003)	0.036 (0.007)	0.048 (0.006)

se: standard error. 5′-end (*Pvmsp-7C*: nucleotides 1–390, *Pvmsp-7H*: nucleotides 1–471, *Pvmsp-7I*: nucleotides 1–525), central (*Pvmsp-7C*: nucleotides 391–717, *Pvmsp-7H*: nucleotides 472–771, *Pvmsp-7I*: nucleotides 526–789) and 3′-end (*Pvmsp-7C*: nucleotides 718–1,191, *Pvmsp-7H*: nucleotides 772–1,200, *Pvmsp-7I*: nucleotides 790–1,188).

* : p<0.04, **: p<0.001, •: p<0.0001.

Furthermore, several maximum likelihood-based methods were used for identifying which codon sites were under positive or negative selection. *Pvmsp-7C* had 19 sites under positive selection and 21 negatively selected sites according to SCAL, FEL, IFEL and REL methods. Positive selection signatures were found for *Pvmsp-7H* in 50 sites while another 32 sites were negatively selected and *Pvmsp-7I* had 10 sites under positive selection and 30 negatively selected sites (predicted by at least one method, Tables S4 and S5). Taking into account that recombination (see below) can affect the reliability of identifying sites under selection [32], the analysis was performed again, this time considering recombination. Two sites were thus positively selected and 43 were under negative selection for *Pvmsp-7C*. Ten sites were positively selected and 24 were negatively selected for *Pvmsp-7H*, and 4 sites were under positive selection while 13 sites were under negative selection for *Pvmsp-7I* (Tables S6 and S7).

### Linkage Disequilibrium (LD) and Recombination

**Figure 4:**
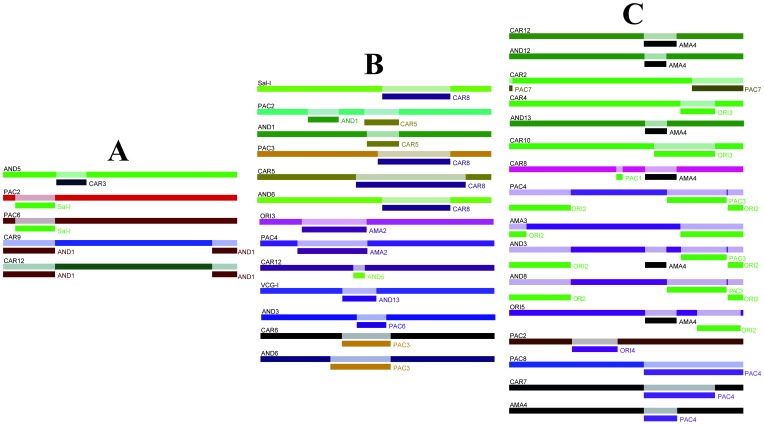
Schematic representation of recombination fragments identified by RDP3 v.3.4 for *Pvmsp-7C* (A), *Pvmsp-7H* (B) and *Pvmsp-7I* (C). The sequence names in black above the rectangles indicate the name of recombinant sequence. The rectangle with name to the right (name of the close relative minor parent) shown in different colors is a graphical representation of a sequence fragment that has potentially been derived through recombination. Only recombination events having p<0.03 were taken into account. SAL-I: Salvador strain, AMA: Amazonian, AND: Andean, CAR: Caribbean, ORI: Orinoco, PAC: Pacific.

Random association was observed among *Pvmsp-7* haplotypes, therefore suggesting their independent segregation. LD analysis measured by Z_nS_ (average of R?^2^) for whole data revealed no statistically significant values for the three *msp-7* genes (Table 1). The relationship of R?^2^ (linkage disequilibrium) with physical distance in regression analysis showed that LD declined as nucleotide distance increased (Fig. S7). The ZZ statistic had significant values (Table 1). Therefore, both the regression analysis and ZZ statistic suggested that intragenic recombination was taking place in *Pvmsp-7C*, *Pvmsp*-*7H* and *Pvmsp*-*7I*. Recombination events were detected by using DnaSP which revealed several RM events in all genes (Table 1). Figure 4 shows the recombinant regions detected with RDP v3.4 software.

### Clustering Analysis

Since recombination was detected for *msp-7* genes, phylogeny had to be inferred taking it into account. A phylogenetic tree was inferred for each recombinant region detected (Fig. S8, S9, S10). All topologies showed no geographical clustering among the Colombian isolates; moreover, Colombian and Salvadorian sequences clustered together (Fig. S8, S9, S10).

All *Pvmsp-7* sequences were aligned and trees were constructed by using ML with the TN93*+G* model. The tree showed three monophyletic groups (Fig. S11A); the first group clustered *Pvmsp-7H* sequences, close to the second group which clustered *Pvmsp-7I* sequences, while the third group clustered *Pvmsp-7C* sequences. This topology agreed with the phylogenetic relationship previously reported for *Pvmsp-7* [18], [27]. However, *Pvmsp-7* sequences showed recombinant fragments possibly produced by gene conversion between paralogous genes at the 5′ and 3′-ends but not in the central region (Fig. S12 and Table S8). The *Pvmsp-7* genes became clustered into paraphyletic groups when trees were inferred taking gene conversion into account (Fig. S11B–E). Codons 38 and 112 in the three genes, as well as the 57, 70, 102 and 125 for *Pvmsp-7C* and *Pvmsp-7H*, and codons 48 and 86 for *Pvmsp-7H* and *Pvmsp-7I* (numbered according to the alignment in Fig. S13) were predicted as negatively selected sites but, taking into account that they lay within the gene conversion tracks detected, purifying selection at these sites could have been the result of gene conversion between paralogous gene loci.

The d_S_ and d_N_ rates between paralogous gene pairs were estimated to determine whether homogeneity at the *Pvmsp-7* ends was caused by concerted evolution (Table 3); the d_S_ was higher than d_N_ at the 5′-end for the three genes. The d_S_ rate at the 3′-end between *Pvmsp-7C* and the other two genes was higher than d_N_ rate but the d_S_ was similar to d_N_ between *Pvmsp-7H* and *Pvmsp-7I* (Table 3).

**Table 3:** Synonymous nucleotide substitution per synonymous site and nonsynonymous nucleotide substitution per nonsynonymous site and the standard deviations between three *Pvmsp-7* genes.

	Synonymous nucleotide substitution per synonymous site (d_S_)								
	5′-end			Central region			3′-end		
	*Pvmsp-7C*	*Pvmsp-7H*	*Pvmsp-7I*	*Pvmsp-7C*	*Pvmsp-7H*	*Pvmsp-7I*	*Pvmsp-7C*	*Pvmsp-7H*	*Pvmsp-7I*
*Pvmsp-7C*									
*Pvmsp-7H*	0.241 (0.050)			1.016 (0.199)			0.879 (0.162)		
*Pvmsp-7I*	0.468 (0.085)	0.518 (0.092)		0.956 (0.167)	0.790 (0.140)		0.788 (0.138)	0.136 (0.031)	
	**Non-synonymous nucleotide substitution per non-synonymous site (d_N_)**								
	**5′-end**			**Central region**			**3′-end**		
	***Pvmsp-7C***	***Pvmsp-7H***	***Pvmsp-7I***	***Pvmsp-7C***	***Pvmsp-7H***	***Pvmsp-7I***	***Pvmsp-7C***	***Pvmsp-7H***	***Pvmsp-7I***
*Pvmsp-7C*									
*Pvmsp-7H*	0.072 (0.017)			1.430 (0.238)			0.234 (0.035)		
*Pvmsp-7I*	0.125 (0.028)	0.152 (0.031)		1.729 (0.244)	1.841 (0.306)		0.323 (0.047)	0.122 (0.028)	

5′-end from nucleotides 1 to 384 (amino acids 1 to 128), central region from nucleotides 385 to 807 (amino acids 129 to 269) and 3′-end from nucleotides 808 to 1,248 (amino acids 270 to 416), numbered according to the alignment in Fig S13.

## Discussion

At least eleven MSPs have been reported in *P. falciparum* and nine *P. vivax* orthologous genes have been described so far. Two *P. vivax* MSP families (*msp-3* and *msp-7*) are particularly interesting since they have expanded differentially [18], [33]. The *P. vivax msp-7* gene family has 11 functional genes [18] and at least 8 of them are transcribed during the *P. vivax* schizont stage [20]. MSP-7 forms part of the main protein complex interacting with host cells [16], [22]. Taking into account that this protein is localized on the parasite membrane [19], [21], [22], [23], it can be targeted by the host immune system and thus becomes an attractive vaccine candidate.

Besides being recognized by the immune system, vaccine candidates must have limited polymorphism. Results found in this study highlighted differences in allele diversity among *msp-7* gene family members, at least in the two main human malaria species. By contrast with the very little polymorphism displayed by *Pfmsp-7* genes [28], [29], *Pvmsp-7* genes (*Pvmsp-7C*, *Pvmsp-7H* and *Pvmsp-7I*) have high genetic diversity. It is worth noting, however, that not all the *Pvmsp-7* genes display a similar pattern. *Pvmsp-7A* and *Pvmsp-7K*, like *Pfmsp-7A*, *Pfmsp-7B*, *Pfmsp-7C*, *Pfmsp-7D*, *Pfmsp-7E*, *Pfmsp-7F* and *Pfmsp-7H* (GenBank ID numbers: XM_001350038.1–44.1) display very low polymorphism [28], [29], [30] suggesting that *msp-7* genes might be exposed to different selective pressure (such as that exerted by the immune system) or have different biological constraints. Although low genetic polymorphism does not hold for *Pvmsp-7C*, *Pvmsp-7H* and *Pvmsp-7I*, vaccine development using these proteins could be focused on their conserved C-terminal domain (Pfam ID number PF12948) due to the high level of conservation it displays and, like in *P. falciparum*, the C-terminal domain could be involved in interaction with erythrocytes [24].

The genetic diversity found in these three genes places them among the most diverse *P. vivax* genes described so far (Table S1). Different allele types were found when Colombian samples were compared with Sal-I (GenBank ID numbers: XM_001614082.1, XM_001614087.1, XM_001614088.1) and unpublished Korean sequences, (GenBank ID numbers: GU476538.1, GU476534.1, GU476518.1 and ACY66906-26) (data not shown), suggesting that such genetic diversity is distributed worldwide. Moreover, no correlation between nucleotide diversity (π) and *P. vivax* incidence in the geographical regions in question was observed (data not shown).

The *Pvmsp-7C* and *Pvmsp-7I* genes appear to be under balancing selection. It has been suggested that this type of selection increases variability within a population [34] maintaining alleles at intermediate frequencies, as can be observed in the alignment. Nevertheless, Tajima and Fu & Li tests are influenced not only by selection but also by the population history that can alter neutral allele frequency expectations. Therefore, the positive values in the tests could have been the result of a decrease in the population. However, a population affected by genetic drift (a mechanism that decreases the population) was not found for the Colombian population (Fs not statically significant). Moreover, the Hd and π values for both genes suggested a stable population having a long-term effective population size; allele frequency distribution would not therefore be influenced by a demographic process.

By contrast with the *Pvmsp-7C* and *Pvmsp-7I* results discussed above, *Pvmsp-7H* appeared to be under a standard neutral model of molecular evolution since there were no significant values in neutrality tests and no difference between d_N_ and d_S_ was found; consequently, high polymorphism would be expected in regions lacking functional constraints [35]. However, a deviation from neutral expectation would only be detected if the average from the whole gene was significantly greater or smaller than 0. The Nei-Gojobori method behaves similarly; positive selection can be detected only if the d_N_ average from the whole gene is significantly greater than d_S_ (the opposite occurs for negative selection). The sliding window test (for neutral statistics and ω) for different selective pressures appeared to be acting throughout *Pvmsp-7* gene sequences, as has been described for others proteins [36], [37], [38], [39]. The tests showed that the 5′- and 3′-ends had purifying selection signals (negative values in the neutrality test and ω <1) while the central region of the three genes had balancing or positive selection signals (positive values in the neutrality test and ω >1). So, despite the loci displayed being under balancing selection or under neutrality, natural selection may have varied across codons. Two different approaches were thus followed to investigate this hypothesis. One estimated the d_N_ and d_S_ rates at the 5′- and 3′-ends and in the central region of the three genes. The central region was under positive selection while the 5′- and 3′-ends seemed to be under negative selection (d_S_ higher than d_N_, only significant for the 5′-end)_._ Similar results were observed when using a second approach in which several maximum likelihood methods were performed to determine positively and negatively selected codons. The central region of the *Pvmsp-7C*, *Pvmsp-7H* and *Pvmsp-7I* genes had codons under positive selection while sites under negative selection were preferentially located at the 5′- and 3′-ends which were relatively fully conserved in *Pvmsp-7* genes.

Besides selective pressure and functional constraints, evolution of malarial antigens might be affected by recombination [13], [14], [39], [40], [41]. Several statistics and algorithms showed that intragenic recombination played an important role in generating new allele variants in individual *msp-7* genes. These events affect the accuracy of detecting selected sites, increasing type I errors [32]. When sequences were screened for recombination, the positively selected sites decreased, suggesting that several of the positively selected sites were false positive. However, some were true positively selected sites (Table S6). These sites did not seem to be originated by the stochastic nature of the mutation process, since it would have been expected that d_N_ substitutions (and/or positive selection) would have been randomly found across the genes; instead, these sites were just observed in the *msp-7* central region and the non-synonymous substitutions in the positively selected sites were found as parsimonious sites and not as singleton sites. Recombination and selection thus seem to drive *Pvmsp-7* genes’ antigenic variation, similar to what occurs in other pathogen antigens [42], [43], [44]. Likewise, it has been shown that both diversification by recombination and purifying selection take place in different regions of the *E. coli fimA* gene [45], and this also occurs in the *Pvmsp-7* genes (Fig. 4 and Table S7).

On the other hand, multigene families might be evolving by recombination among paralogous genes, thereby contributing to allele diversity or the homogenization of the multigene family in the event of unequal crossover or gene conversion [46]. The gene conversion and subsequent phylogenetic analysis revealed that these genes had not evolved independently; several conversion tracks at the 5′- and 3′-ends (but not in the central region) were detected among *msp-7* genes, suggesting that at least these three genes could be evolving by concerted evolution mediated by a biased gene conversion, homogenizing only the 5′ and 3′-ends. Thus, recombination would seem to affect the evolution of the *msp-7* family as a whole, and individual *msp-7* genes. However, protein homogeneity can also be attained by purifying selection. Under the assumption of gene conversion, it would be expected that d_S_ between duplicated genes would be similar to d_N_, whereas if purifying selection is the major evolutionary force, then d_S_ would be much higher than d_N_ [47], According to d_S_ and d_N_ rate comparison, the homogeneity at the *Pvmsp-7* genes’ 5′-end was apparently caused by functional constraints rather than by concerted evolution, since *Pvmsp-7* genes have diverged extensively by silent nucleotide substitution at these ends. The conservation of the 3′-end in *Pvmsp-7H* and *Pvmsp-7I* but not in *Pvmsp-7C* seems to be maintained by gene conversion. This behavior might be a consequence of closely spaced gene duplicates being more likely to undergo gene conversion [48], [49]. *Pvmsp-7H* and *Pvmsp-7I* genes are neighbors separated by 1,086 base pairs while the *Pvmsp-7C* gene is separated from the others by 9,619 base pairs. Accordingly, the high *Pvmsp-7*s’ C-terminal conservation may have been the result of functional constraints and purifying selection, or the result of gene conversion (between *Pvmsp-7H* and *Pvmsp-7I*) possibly due to the presence of a functional domain (Pfam ID number PF12948) within this region. Therefore, the central region (the most diverse) could have been under selective pressure exerted by the immune system; consequently, the intragenic recombination and, to a lesser extent, the positively selected sites increased genetic diversity, generating different allele variants as an evasion mechanism.

Phylogenetic analysis showed that, regardless of an isolate’s origin, sequences tended to cluster without having clear geographical distribution. This pattern might indicate a constant *P. vivax* gene flow in Colombian regions. This result agreed with previous reports of other highly polymorphic *P. vivax* genes not involved in geographical clustering [13], [14], [39].

Previous studies have shown the potential role of MSP-7 in parasite invasion of erythrocytes [19], [24], [25], [50] which, added to its immunogenicity [26], [31], and following the rules for subunit-based vaccine development [51], [52], make the MSP-7 conserved domain an attractive candidate to be evaluated when designing an antimalarial vaccine.

## Materials and Methods

### Ethics Statement

All *P. vivax*-infected patients who provided us with the blood samples signed an informed consent and the purpose of the study was carefully explained to them. All procedures carried out in this study were approved by our institute’s ethics committee.

### Source of Parasite DNA and Field Isolate Genotyping

Peripheral blood samples from patients proving positive for *P. vivax* malaria by microscope examination were collected from geographical regions of Colombia from 2007 to 2010. DNA was obtained using a Wizard Genomic DNA Purification kit (Promega) following manufacturer’s instructions.

All parasite samples were genotyped by PCR-RFLP of the *Pvmsp-1* gene’s blocks 6, 7 and 8 as previously described [53] for selecting only samples having a single *P. vivax msp-1* allele infection.

### Amplification and Sequencing

Primers were designed to amplify *Pvmsp-7C*, *Pvmsp-7H* and *Pvmsp-7I* DNA fragments based on the Sal-I reference sequences (GenBank IDs: XM_001614082.1, XM_001614087.1 and XM_001614088.1, respectively). The DNA fragment from *Pvmsp-7C* was amplified with 7Cdto 5′ ACCACAAAGATGAATAAAACG 3′ and 7Crev 5′ CACCTCAATCGTGTTCAGC 3′ primers. *Pvmsp-7H* was amplified by using 7Hdto 5′ GTGTGCATCAGTATAGCGAC 3′ and 7Hrev 5′ AAGAAGGTTAGCCATAAATGC 3′ primers and *Pvmsp-7I* was amplified with 7Idto 5′ ACAATGAGGGGCAAGTACG 3′ and 7Irev 5′ TTCATTCGTTGCTCACTTCG 3′ primers. All PCR reactions were performed using KAPA HiFi HotStart Readymix containing 0.3 µM of each primer in a final 25 µL volume. Thermal conditions were set as follows: one cycle of 5 min at 95°C, 25 cycles of 20 sec at 98°C, 15 sec at 62°C for *Pvmsp-7C* and *Pvmsp-7*I and 60°C for *Pvmsp-7H*, 30 sec at 72°C, followed by a 5 min final extension at 72°C. PCR products were purified using the Wizard PCR preps kit (Promega), and then sequenced with a BigDye Terminator kit (MACROGEN, Seoul, South Korea) in both directions. Internal primers were used for sequencing (intdc 5′ CTGTTGGACCCGGTGGAG3′, intrc 5′ CTTGTTGATTCGCTCCTGG 3′; intdh 5′ TCAAATACAGCACAGACTTCC, intrh 5′ CCTCAGGACAACCCGAAAG 3′; intdi 5′ TCACAAACGCACAACCCAGG 3′ and 5′ GCTCCATTACCACAACCGG 3′). Two PCR products obtained from independent PCR amplifications were sequenced per isolate to discard errors.

### Statistical Analysis for the *msp-7* Sequences

Electropherograms were assembled using CLC DNA workbench 6 (CLC bio, Cambridge, MA, USA) and Clustal W [54] was used for aligning the deduced amino acid sequences, followed by manual editing. Additionally, repeats with 90% similarity in the deduced *msp-7* amino acid sequences were detected by using the T-REKS algorithm [55]. The PAL2NAL program [56] was used for constructing codon alignments from the corresponding aligned amino acid sequences. Colombian *P. vivax msp-7C, msp-7H* and *msp-7I* sequences were compared against the previously described Sal-I strain sequences available in GenBank (ID numbers: XM_001614082.1, XM_001614087.1, and XM_001614088.1).

DNA polymorphism was calculated with DnaSP v.5 software [57]. Tests to assess departure from the neutral model were applied using Tajima’s D and Fu & Li’s D* and F* statistics. The former statistic compares the differences between the total number of segregating sites and the average number of nucleotide differences between sequence pairs. The Fu & Li test calculates the D* statistic which is based on the difference between the number of singletons and the total number of mutations, as well as the F* statistic which is based on the difference between the number of singletons and the average number of nucleotide differences between sequence pairs. Positive and negative values from both tests correspond to departures from neutrality. The Fs statistic [58] was used; it is based on gene frequency distribution. All tests were applied using DnaSP v.5 software, considering coalescent simulations for obtaining confidence intervals [57]. Natural selection was estimated using the modified Nei-Gojobori method [59] to calculate the average number of non-synonymous (d_N_) and synonymous (d_S_) substitutions. Differences between d_N_ and d_S_ were assessed by applying the Z-test using MEGA software v.5 [60]. Additionally, codon sites under positive or negative selection at the population level were assessed by using Datamonkey web server [61] with IFEL, a codon-based maximum likelihood method [62]. This method infers selective pressure at population level; positive or negatively selected sites were assessed by FEL, SLAC and REL methods [63]. All algorithms estimated the ω (d_N_/d_S_ ratio) at every codon in the alignment. A ≤0.1 p-value was considered significant for IFEL, FEL and SLAC methods and ≥50 Bayes factor for REL. Only Colombian sequences were considered for all analyses performed; positions containing gaps or repeats in the alignment were not taken into account (Table S9).

Linkage disequilibrium (LD) was evaluated by calculating the Z_nS_ statistic [64] which is the average of R?^2^ over all pairwise comparisons. A lineal regression between LD (R?^2^) and nucleotide distances was performed to evaluate whether recombination was taking place in *Pvmsp-7* genes. Recombination events were assessed using DnaSP v.5 software [57] applying the ZZ statistic [65] and RM parameter [66]. The latter statistic incorporates the effective population size and probability of recombination between adjacent nucleotides per generation. RDP3 v3.4 software [67] was used for detecting recombination regions in *msp-7* genes. This tool looks for evidence of recombination among aligned sequences by examining all possible triplet combinations following a scanning approach with a range of different recombination detection algorithms. Additionally, the algorithm developed by Betrán *et al.* (1997) [68] incorporated in DnaSP [57] and the GENECONV program [69] incorporated in RDP3 v3.4 software [67] were used to detect gene conversion among paralogous genes.

### Geographical Clustering

Maximum Likelihood (ML) trees describing the phylogenetic consequences of the recombination events were constructed using RDP3 v3.4 software [67] with the HKY model (selected by the ModelTest algorithm [70]) to evaluate relationships between polymorphism and the geographical distribution of the isolates. Additionally, *Pvmsp-7C*, *Pvmsp-*7*H* and *Pvmsp-7I* sequences were aligned and trees were constructed by using ML with the TN93*+G* model selected by the ModelTest algorithm [70]. Bootstrap analysis (with 1,000 replicates each) was used for assigning confidence levels to branch nodes. Positions containing gaps as well as regions in the alignment that contained repeats were not taken into account in the phylogenetic analysis (Table S9).

## Supporting Information

Figure S1 **Geographical location of the study regions within Colombia.** Amazonian region (samples from Calamar in the Guaviare department), Andean region (samples from Apartadó and El Bagre in the Antioquia department), Caribbean region (samples from Tierra Alta and Puerto Libertador in the Córdoba department), Orinoco region (samples from Tauramena in the Casanare department and Mapiripán in the Meta department), and Pacific region (samples from Istmina in the Chocó department and Tumaco in the Nariño department). Black dots on the map represent the areas from which patients came who donated the infected blood samples. 1: Puerto Libertador, 2: Tierra Alta, 3: Istmina, 4: Tumaco, 5: Apartado, 6: El Bagre, 7: Tauramena, 8: Mapiripan, 9: Calamar.
(TIF)

Figure S2 Sliding window analysis of Tajima D and Fu & Li D* and F* statistics along the ***Pvmsp-7C*** **(A),** ***Pvmsp-7H*** **(B) and** ***Pvmsp-7I*** **(C) genes.** Bars [Tajima’s D (blue), Fu & Li’s D* (red) and F* (green)] below each figure represent the regions where the tests showed a significant deviation from the neutral expectation. 5′-end (*Pvmsp-7C*: nucleotides 1–390, *Pvmsp-7H*: nucleotides 1–471, *Pvmsp-7I*: nucleotides 1–525), central (*Pvmsp-7C*: nucleotides 391–717, *Pvmsp-7H*: nucleotides 472–771, *Pvmsp-7I*: nucleotides 526–789) and 3′-end (*Pvmsp-7C*: nucleotides 718–1,191, *Pvmsp-7H*: nucleotides 772–1,200, *Pvmsp-7I*: nucleotides 790–1,188).
(PDF)

Figure S3 **Sliding window analysis for ω rates (d_N_/d_S_) throughout the** ***Pvmsp-7C*** **(Blue),** ***Pvmsp-7H*** **(Red) and** ***Pvmsp-7I*** **(Green) genes.** Discontinuity of the curves is due to gaps within the alignments which were not considered for the analysis. 5′-end (*Pvmsp-7C*: nucleotides 1–390, *Pvmsp-7H*: nucleotides 1–471, *Pvmsp-7I*: nucleotides 1–525), central (*Pvmsp-7C*: nucleotides 391–717, *Pvmsp-7H*: nucleotides 472–771, *Pvmsp-7I*: nucleotides 526–789) and 3′-end (*Pvmsp-7C*: nucleotides 718–1,191, *Pvmsp-7H*: nucleotides 772–1,200, *Pvmsp-7I*: nucleotides 790–1,188).
(PDF)

Figure S4 **The linkage disequilibrium (LD) plot for** ***P. vivax Pvmsp-7C*** **(A),** ***Pvmsp-7H*** **(B) and** ***Pvmsp-7I*** **(C).** Trace line represents the regression line which declined as nucleotide distance increased suggesting that intragenic recombination was taking place in *msp-7* genes.
(PDF)

Figure S5 **ML trees describing the phylogenetic consequences of the intragenic recombination in** ***Pvmsp-7C*** **.** A topology is inferred for each recombinant fragment, (A) from nucleotides 685 to 855, (B) from nucleotides 1,060 to 1,167 (excluding nucleotides 1,105 to 1,107) and from 18 to 266, and (C) from nucleotides 62 to 266. Isolates clustered without a clear geographical distribution. Sal-I: Salvador strain, AMA: Amazon, AND: Andean, CAR: Caribbean, ORI: Orinoco, PAC: Pacific.
(TIF)

Figure S6 **ML trees describing the phylogenetic consequences of the intragenic recombination in** ***Pvmsp-7H*** **.** A topology is inferred for each recombinant fragment, (A) nucleotides 189 to 543 (excluding nucleotides 436 to 450 and nucleotides 484 to 486), (B) nucleotides 353 to 661 (excluding nucleotides 436 to 450 and 568 to 627), (C) nucleotides 425 to 655 (excluding nucleotides 436 to 450, 484 to 486 and 568 to 627), (D) nucleotides 500 to 1,057 (excluding nucleotides 568 to 627 and 772 to 774), (E) nucleotides 628 to 977 (excluding nucleotides 772 to 774), (F) nucleotides 630 to 997, (G) nucleotides 416 to 590 (excluding nucleotides 436 to 450 and 484 to 486), (H) nucleotides 472 to 531 (excluding nucleotides 484 to 486), and (I) nucleotides 482 to 646 (excluding nucleotides 568 to 627). Isolates clustered without a clear geographical distribution. Sal-I: Salvador strain, AMA: Amazon, AND: Andean, CAR: Caribbean, ORI: Orinoco, PAC: Pacific.
(TIF)

Figure S7 **ML trees describing the phylogenetic consequences of the intragenic recombination in** ***Pvmsp-7I*** **.** A topology is inferred for each recombinant fragment identified, (A) nucleotides 19 to 187 and nucleotides 683 to 1,188, (B) nucleotides 683 to 849, (C) nucleotides 684 to 797, (D) nucleotides 19 to 358 and nucleotides 796 to 1,188, (E) nucleotides 798 to 1,105, (F) nucleotides 868 to 1,188, (G) nucleotides 920 to 1,188, (H) nucleotides 733 to 1,044, (I) nucleotides 688 to 797, and (J) nucleotides 683 to 849. Isolates clustered without a clear geographical distribution. SAL-I: Salvador strain, AMA: Amazon, AND: Andean, CAR: Caribbean, ORI: Orinoco, PAC: Pacific.
(TIF)

Figure S8 (A) Phylogenetic tree obtained by ML for *Pvmsp-7* sequences based on the TN93*+G* model, ignoring recombination. Three monophyletic groups are shown; the first groups clustered *Pvmsp-7H* (red) sequences, the second group clustered *Pvmsp-7I* (green) sequences and the third group clustered *Pvmsp-7C* (blue) sequences. (B-E) Trees describing phylogenetic consequences of some gene conversion tracks identified. (B) nucleotides 71 to 265 (amino acids 24 to 89), (C) nucleotides 913 to 1,090 (amino acids 305 to 390), (D) nucleotides 913 to 1,090 (amino acids 305 to 354), (E) nucleotides 913 to 1,175 (amino acids 305 to 392). Positions are numbered according to the alignment in Fig. S13. These topologies suggest that at least *Pvmsp-7C*, *Pvmsp-7H* and *Pvmsp-7I* genes did not evolve independently. Numbers represent bootstrap values with 1,000 replicates.
(PDF)

Figure S9 **Schematic representation of gene conversion tracks identified by DnaSP and GENECONV for** ***Pvmsp-7C*** **(blue),** ***Pvmsp-7H*** **(red) and** ***Pvmsp-7I*** **(green) as a combined data set.** Rectangles in a different color are graphical representations of sequence fragments that have potentially been originated by gene conversion and are localized at the conserved 5′- and 3′-ends. The black bars delimit the 5′-end, the central region and the 3′-end, [5′-end (*Pvmsp-7C*: nucleotides 1–390, *Pvmsp-7H*: nucleotides 1–471, *Pvmsp-7I*: nucleotides 1–525), central (*Pvmsp-7C*: nucleotides 391–717, *Pvmsp-7H*: nucleotides 472–771, *Pvmsp-7I*: nucleotides 526–789) and 3′-end (*Pvmsp-7C*: nucleotides 718–1,191, *Pvmsp-7H*: nucleotides 772–1,200, *Pvmsp-7I*: nucleotides 790–1,188)].
(PDF)

Figure S10 **Alignment of deduced PvMSP-7C amino acid sequences.**
(PDF)

Figure S11 **Alignment of deduced PvMSP-7H amino acid sequences.**
(TIF)

Figure S12 **Alignment of deduced PvMSP-7C amino acid sequences.**
(TIF)

Figure S13 **Alignment of the paralogous PvMSP-7deduced amino acid sequences.**
(PDF)

Table S1 **Nucleotide diversity for** ***P. vivax*** **antigens. n: number of isolates, π: nucleotide diversity.**
(PDF)

Table S2 **Nucleotide diversity (π) values for subpopulations within Colombia.**
(PDF)

Table S3 **Nucleotide and amino acid positions within the 5′- end, central region and 3′-end.**
(PDF)

Table S4 **Positively selected sites detected for** ***Pvmsp-7*** **genes without taking recombination into account.** Numbers according to the reference Sal-I protein sequence *Pvmsp-7C*: XP_001614132.1, *Pvmsp-7H*: XP_001614137.1 and *Pvmsp-7I*: XP_001614138.1.
(PDF)

Table S5 **Negatively selected sites detected for** ***Pvmsp-7*** **genes without taking recombination into account.** Numbers according to the reference Sal-I protein sequence *Pvmsp-7C*: XP_001614132.1, *Pvmsp-7H*: XP_001614137.1 and *Pvmsp-7I*: XP_001614138.1.
(PDF)

Table S6 **Positively selected sites detected for** ***Pvmsp-7*** **genes taking recombination into account.** Numbers according to the reference Sal-I protein sequence *Pvmsp-7C*: XP_001614132.1, *Pvmsp-7H*: XP_001614137.1 and *Pvmsp-7I*: XP_001614138.1.
(PDF)

Table S7 **Negatively selected sites detected for** ***Pvmsp-7*** **genes taking recombination into account.** Numbers according to the reference Sal-I protein sequence *Pvmsp-7C*: XP_001614132.1, *Pvmsp-7H*: XP_001614137.1 and *Pvmsp-7I*: XP_001614138.1.
(PDF)

Table S8 **Conversion tracks identified by DnaSP and GENECONV, between** ***Pvmsp-7*** **genes.** Nucleotides and amino acids based in alignment of the Fig. S13.
(PDF)

Table S9 **Nucleotides and amino acid positions excluded from the analysis.**
(PDF)
