# Supplementary figures and images for: Genetic Diversity and Selection in Three Plasmodium vivax Merozoite Surface Protein 7 (Pvmsp-7) Genes in a Colombian Population
Source: PLoS One. 2012 Sep 25;7(9):e45962. doi: 10.1371/journal.pone.0045962 (PMC3458108; doi:10.1371/journal.pone.0045962)

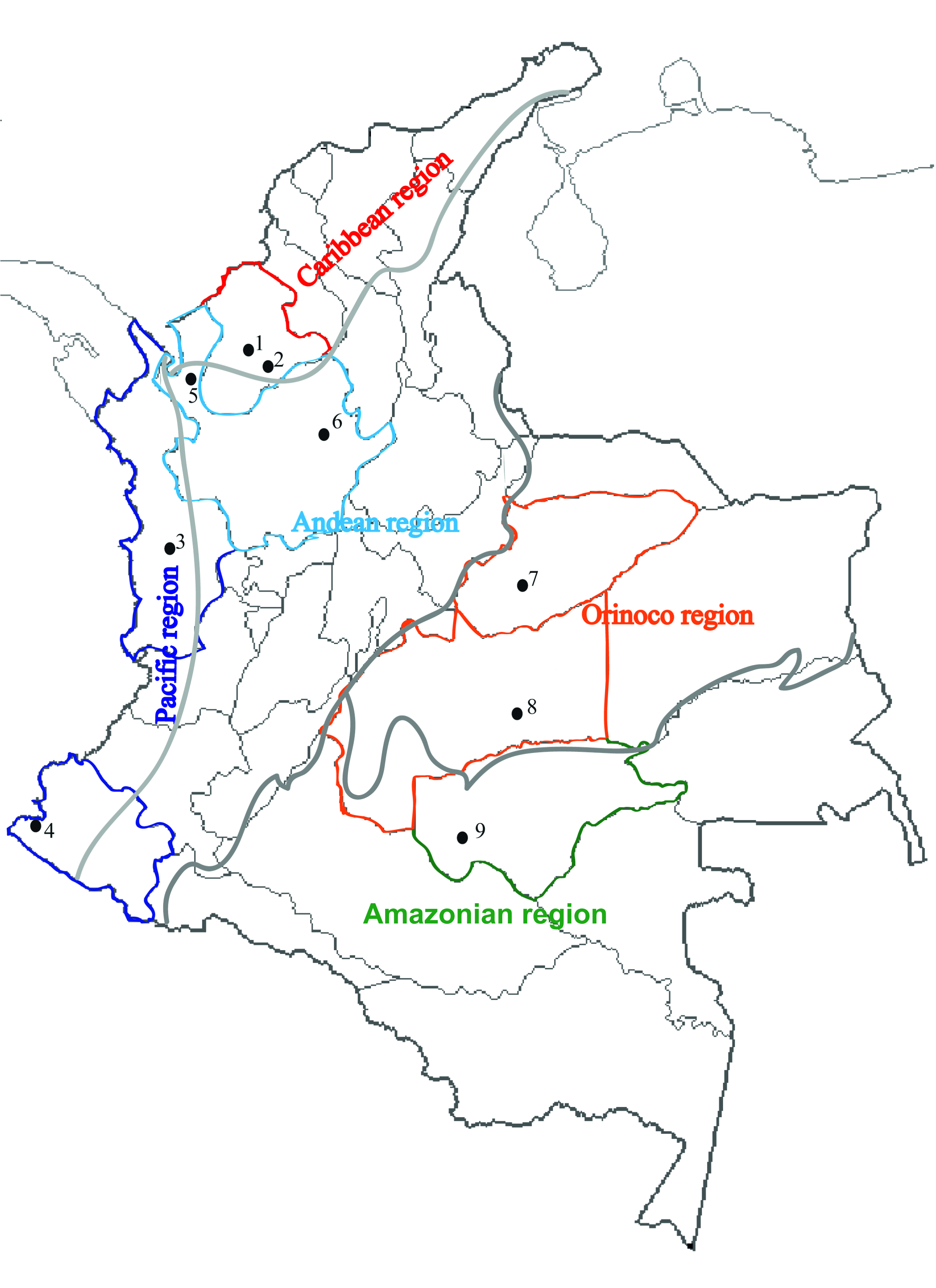

Supplement: Figure S1 — Geographical location of the study regions within Colombia. Amazonian region (samples from Calamar in the Guaviare department), Andean region (samples from Apartadó and El Bagre in the Antioquia department), Caribbean region (samples from Tierra Alta and Puerto Libertador in the Córdoba department), Orinoco region (samples from Tauramena in the Casanare department and Mapiripán in the Meta department), and Pacific region (samples from Istmina in the Chocó department and Tumaco in the Nariño department). Black dots on the map represent the areas from which patients came who donated the infected blood samples. 1: Puerto Libertador, 2: Tierra Alta, 3: Istmina, 4: Tumaco, 5: Apartado, 6: El Bagre, 7: Tauramena, 8: Mapiripan, 9: Calamar. (TIF) [file pone.0045962.s001.tif]

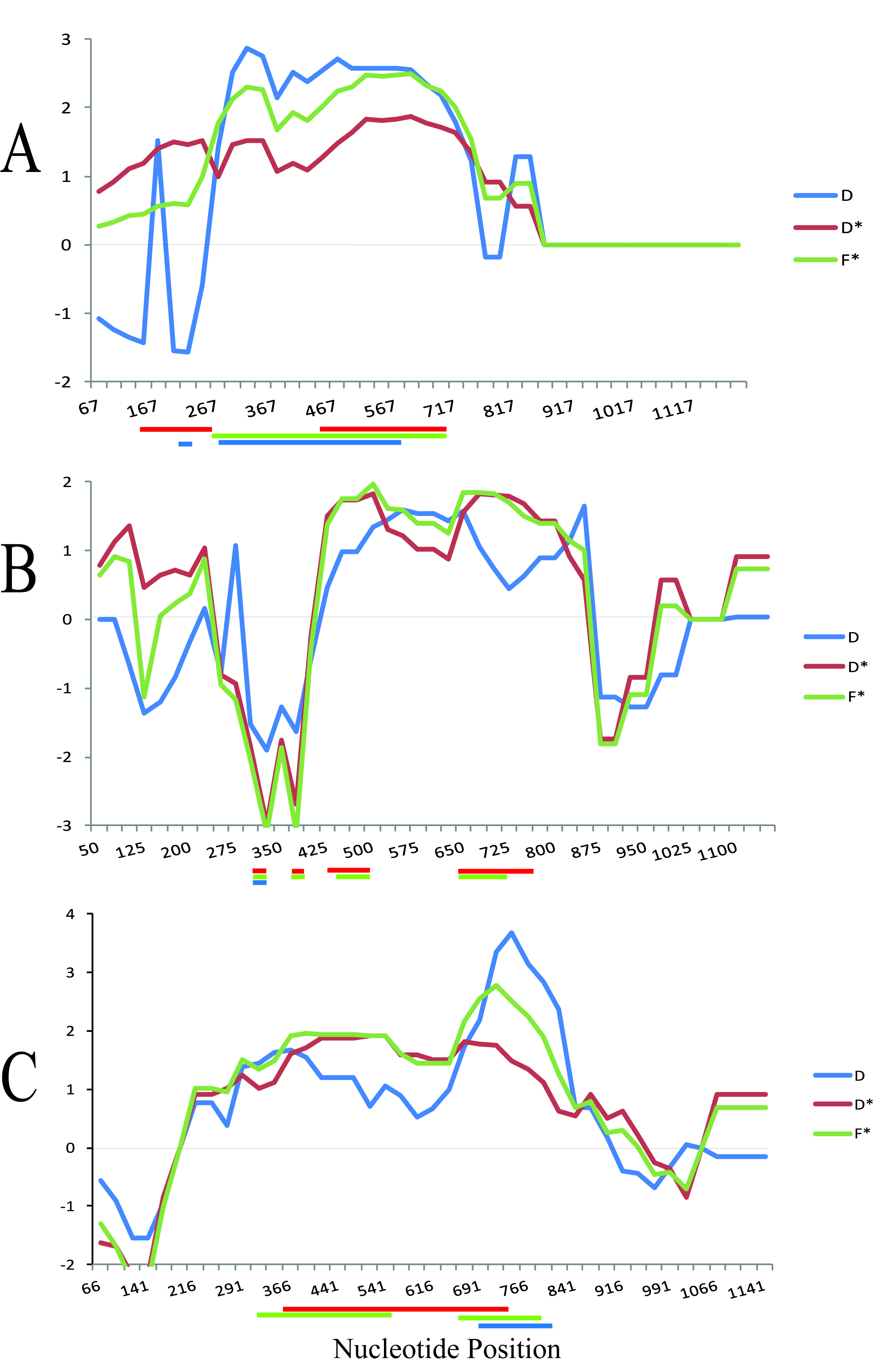

Supplement: Figure S5 — ML trees describing the phylogenetic consequences of the intragenic recombination in Pvmsp-7C . A topology is inferred for each recombinant fragment, (A) from nucleotides 685 to 855, (B) from nucleotides 1,060 to 1,167 (excluding nucleotides 1,105 to 1,107) and from 18 to 266, and (C) from nucleotides 62 to 266. Isolates clustered without a clear geographical distribution. Sal-I: Salvador strain, AMA: Amazon, AND: Andean, CAR: Caribbean, ORI: Orinoco, PAC: Pacific. (TIF) [file pone.0045962.s005.tif]

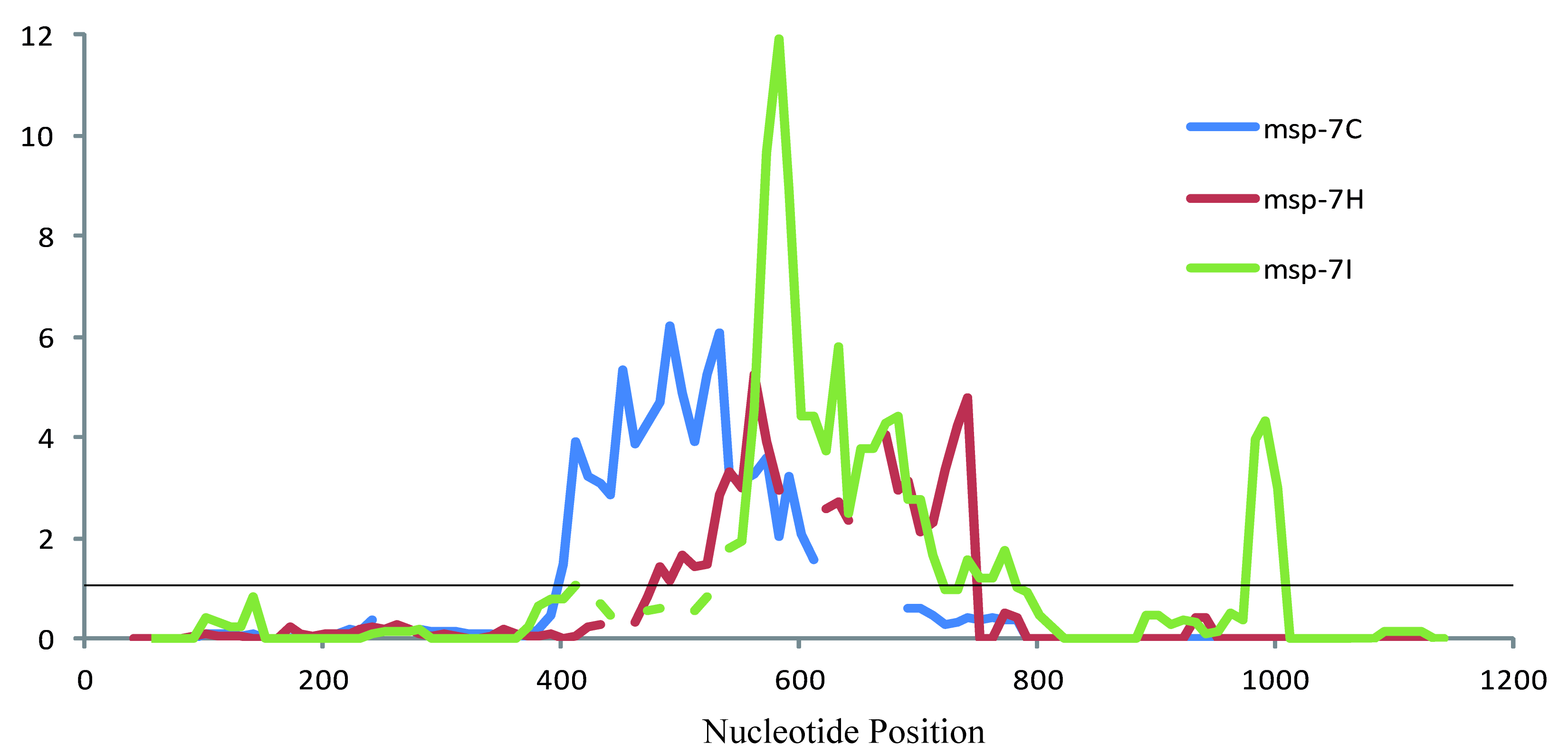

Supplement: Figure S6 — ML trees describing the phylogenetic consequences of the intragenic recombination in Pvmsp-7H . A topology is inferred for each recombinant fragment, (A) nucleotides 189 to 543 (excluding nucleotides 436 to 450 and nucleotides 484 to 486), (B) nucleotides 353 to 661 (excluding nucleotides 436 to 450 and 568 to 627), (C) nucleotides 425 to 655 (excluding nucleotides 436 to 450, 484 to 486 and 568 to 627), (D) nucleotides 500 to 1,057 (excluding nucleotides 568 to 627 and 772 to 774), (E) nucleotides 628 to 977 (excluding nucleotides 772 to 774), (F) nucleotides 630 to 997, (G) nucleotides 416 to 590 (excluding nucleotides 436 to 450 and 484 to 486), (H) nucleotides 472 to 531 (excluding nucleotides 484 to 486), and (I) nucleotides 482 to 646 (excluding nucleotides 568 to 627). Isolates clustered without a clear geographical distribution. Sal-I: Salvador strain, AMA: Amazon, AND: Andean, CAR: Caribbean, ORI: Orinoco, PAC: Pacific. (TIF) [file pone.0045962.s006.tif]

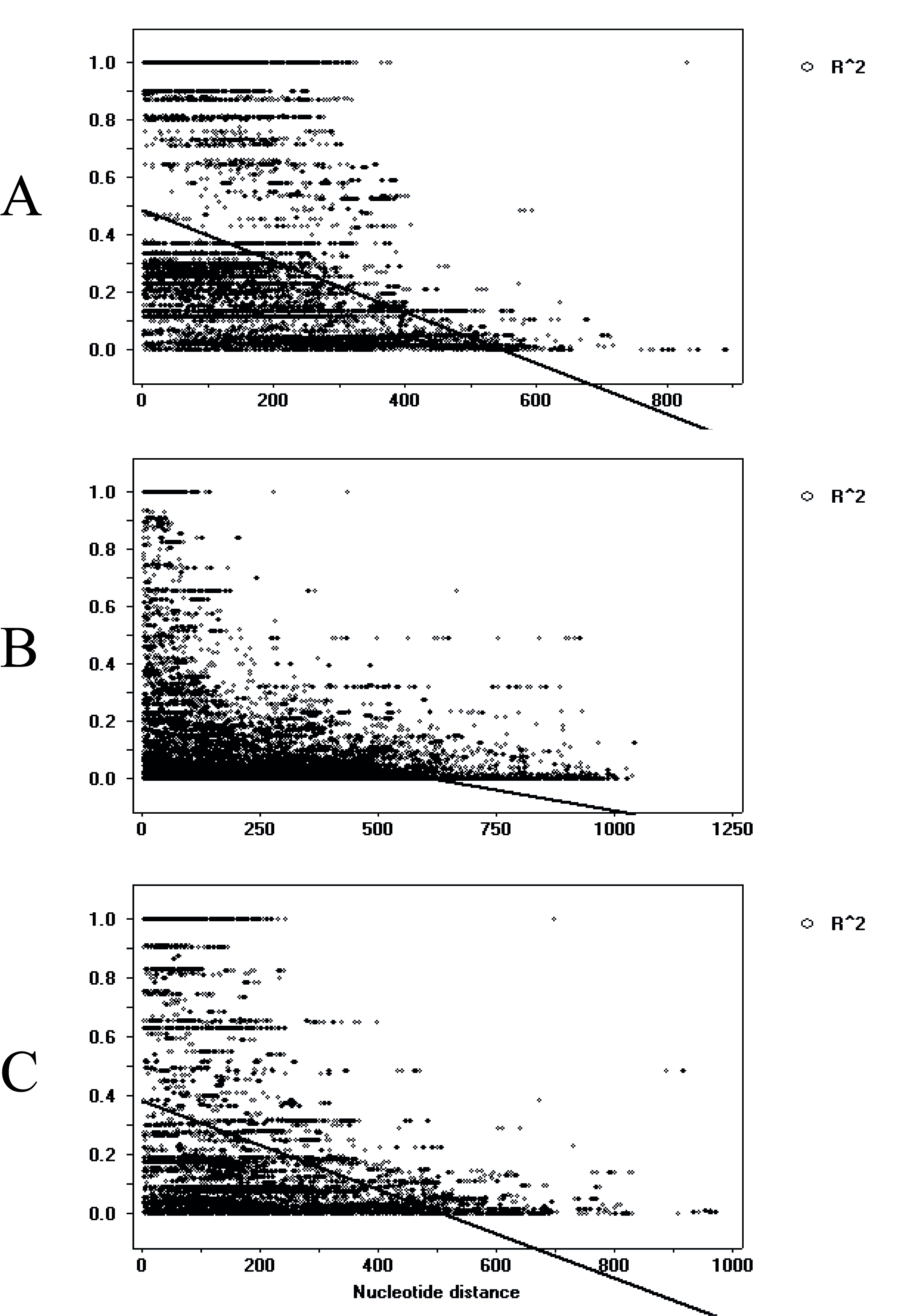

Supplement: Figure S7 — ML trees describing the phylogenetic consequences of the intragenic recombination in Pvmsp-7I . A topology is inferred for each recombinant fragment identified, (A) nucleotides 19 to 187 and nucleotides 683 to 1,188, (B) nucleotides 683 to 849, (C) nucleotides 684 to 797, (D) nucleotides 19 to 358 and nucleotides 796 to 1,188, (E) nucleotides 798 to 1,105, (F) nucleotides 868 to 1,188, (G) nucleotides 920 to 1,188, (H) nucleotides 733 to 1,044, (I) nucleotides 688 to 797, and (J) nucleotides 683 to 849. Isolates clustered without a clear geographical distribution. SAL-I: Salvador strain, AMA: Amazon, AND: Andean, CAR: Caribbean, ORI: Orinoco, PAC: Pacific. (TIF) [file pone.0045962.s007.tif]

A

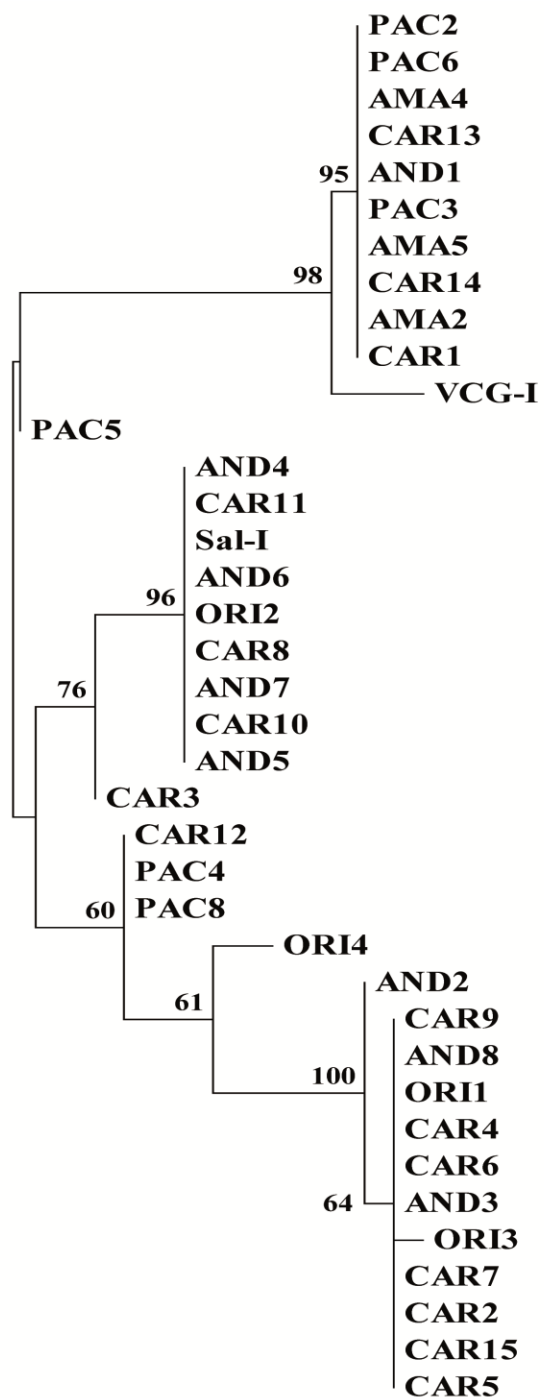

B

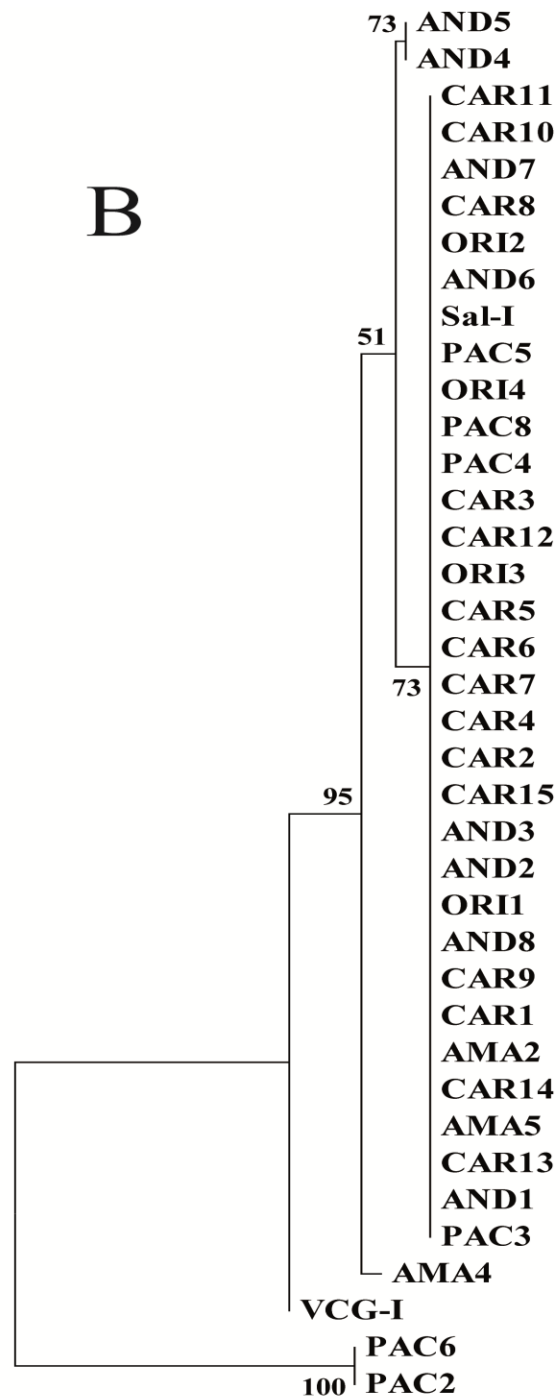

C

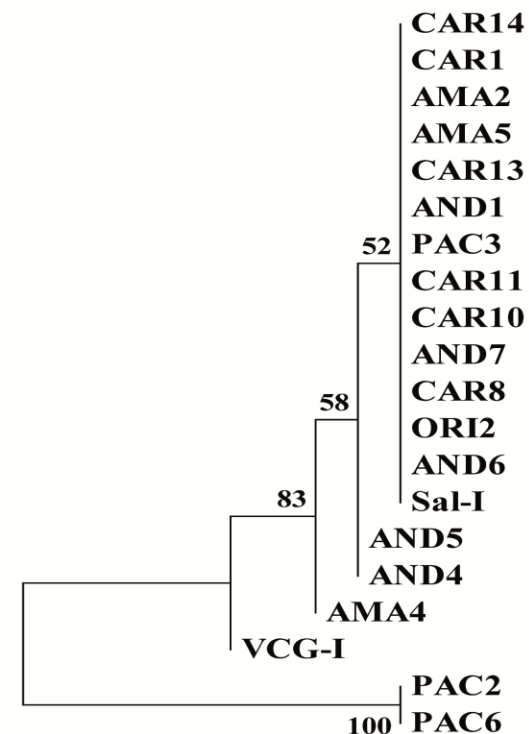

Supplement: Figure S8 — (A) Phylogenetic tree obtained by ML for Pvmsp-7 sequences based on the TN93+G model, ignoring recombination. Three monophyletic groups are shown; the first groups clustered Pvmsp-7H (red) sequences, the second group clustered Pvmsp-7I (green) sequences and the third group clustered Pvmsp-7C (blue) sequences. (B-E) Trees describing phylogenetic consequences of some gene conversion tracks identified. (B) nucleotides 71 to 265 (amino acids 24 to 89), (C) nucleotides 913 to 1,090 (amino acids 305 to 390), (D) nucleotides 913 to 1,090 (amino acids 305 to 354), (E) nucleotides 913 to 1,175 (amino acids 305 to 392). Positions are numbered according to the alignment in Fig. S13. These topologies suggest that at least Pvmsp-7C, Pvmsp-7H and Pvmsp-7I genes did not evolve independently. Numbers represent bootstrap values with 1,000 replicates. (PDF) [file pone.0045962.s008.pdf]

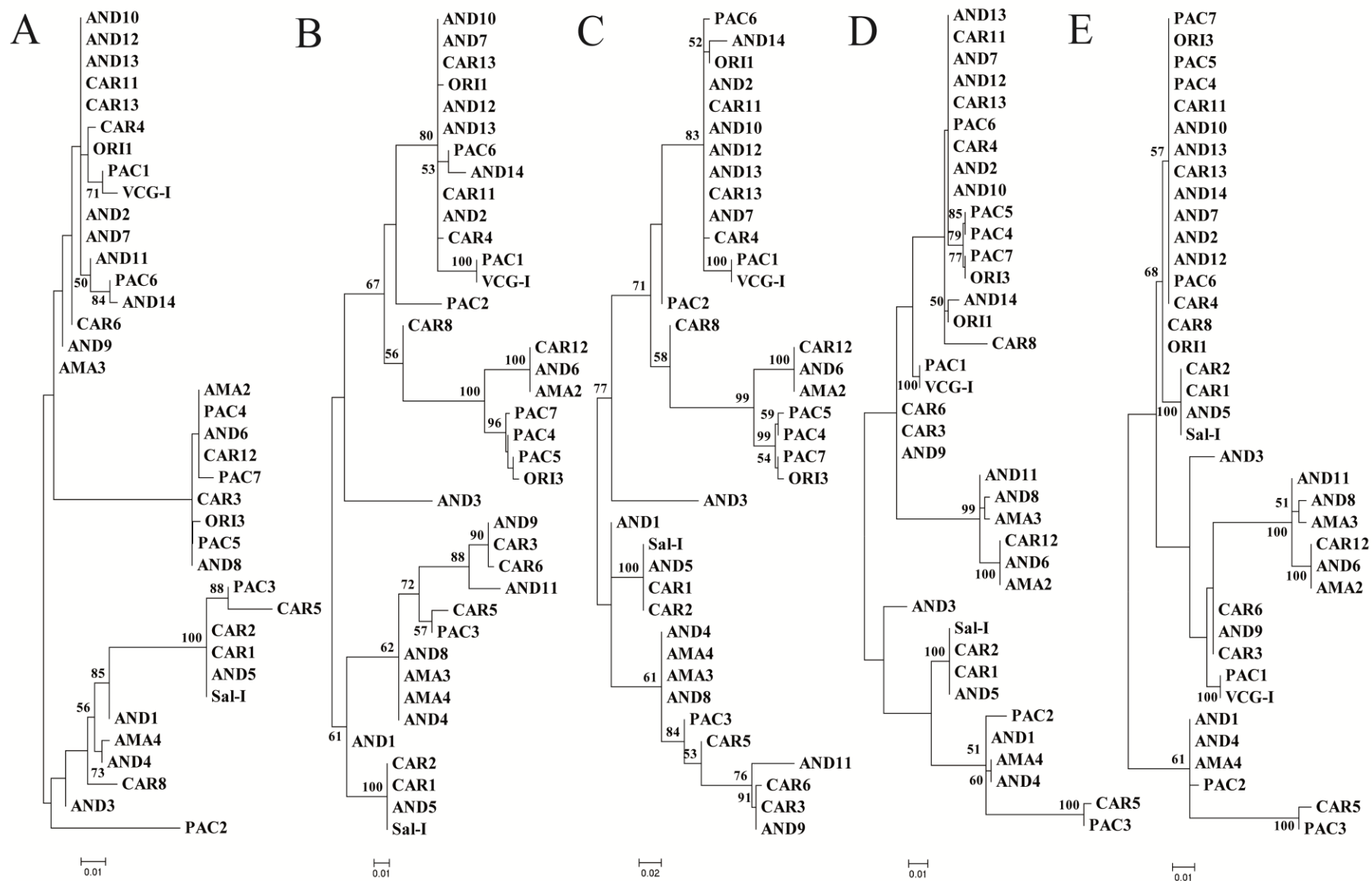

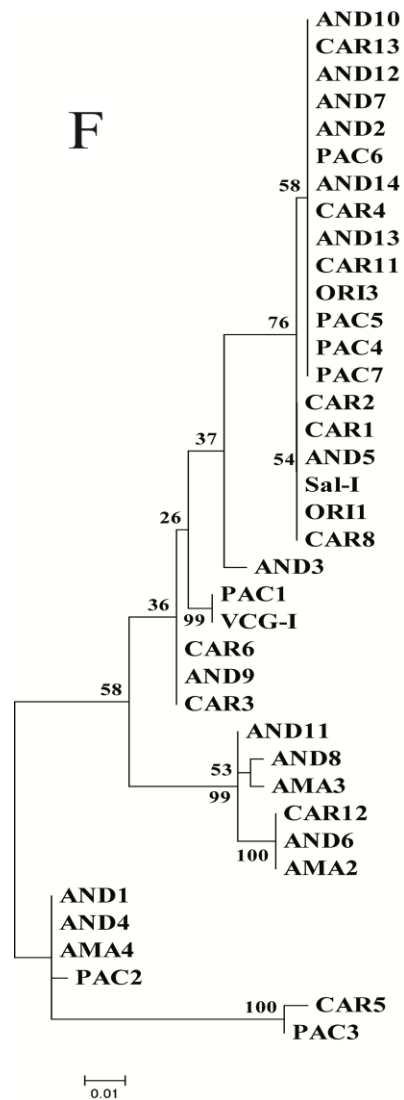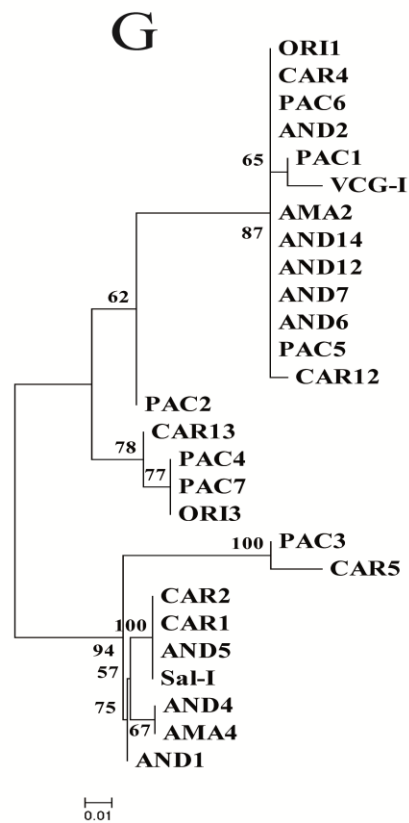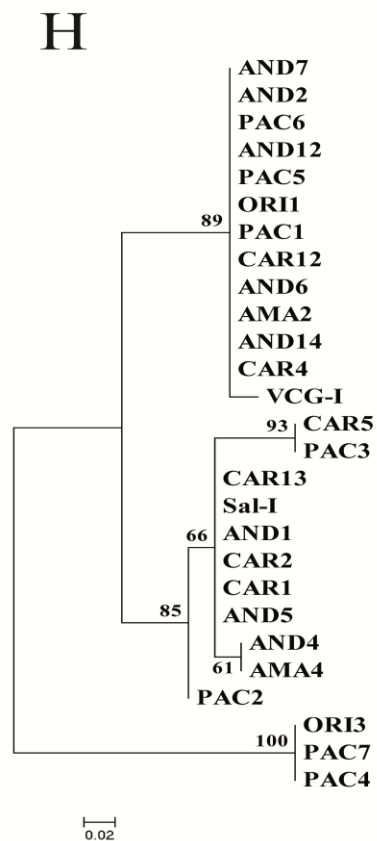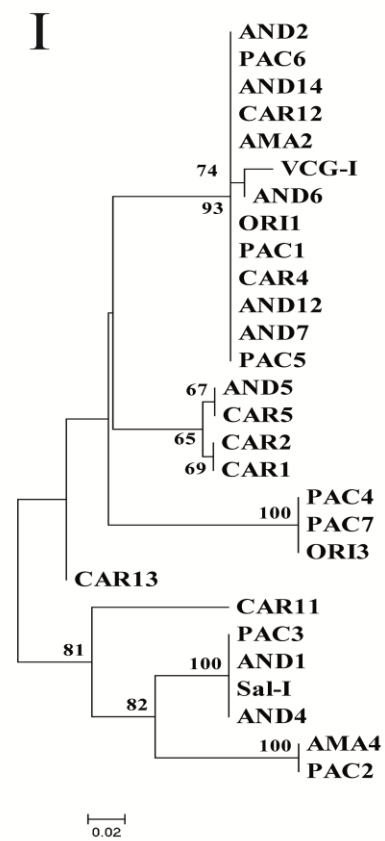

Supplement: Figure S9 — Schematic representation of gene conversion tracks identified by DnaSP and GENECONV for Pvmsp-7C (blue), Pvmsp-7H (red) and Pvmsp-7I (green) as a combined data set. Rectangles in a different color are graphical representations of sequence fragments that have potentially been originated by gene conversion and are localized at the conserved 5′- and 3′-ends. The black bars delimit the 5′-end, the central region and the 3′-end, [5′-end (Pvmsp-7C: nucleotides 1–390, Pvmsp-7H: nucleotides 1–471, Pvmsp-7I: nucleotides 1–525), central (Pvmsp-7C: nucleotides 391–717, Pvmsp-7H: nucleotides 472–771, Pvmsp-7I: nucleotides 526–789) and 3′-end (Pvmsp-7C: nucleotides 718–1,191, Pvmsp-7H: nucleotides 772–1,200, Pvmsp-7I: nucleotides 790–1,188)]. (PDF) [file pone.0045962.s009.pdf]

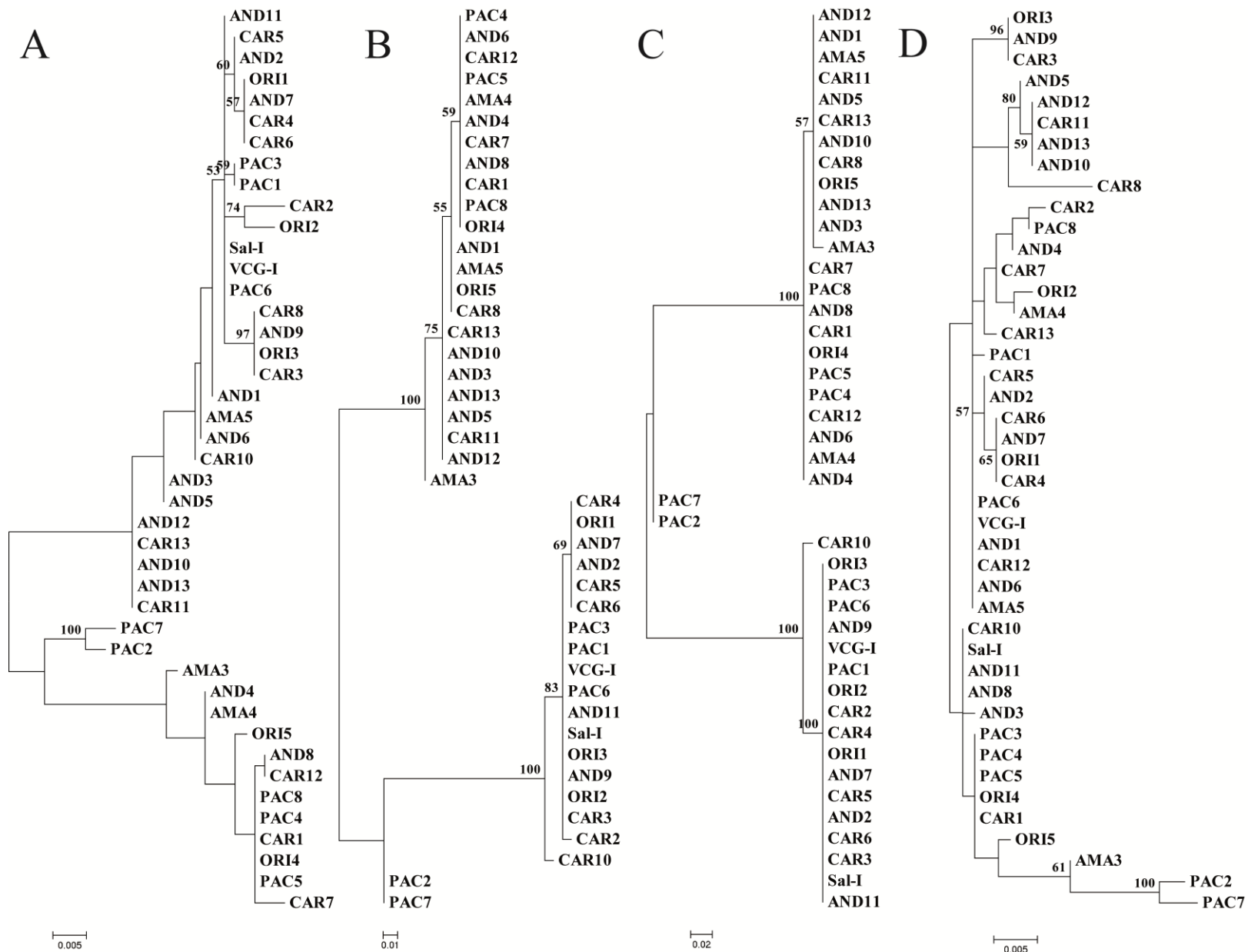

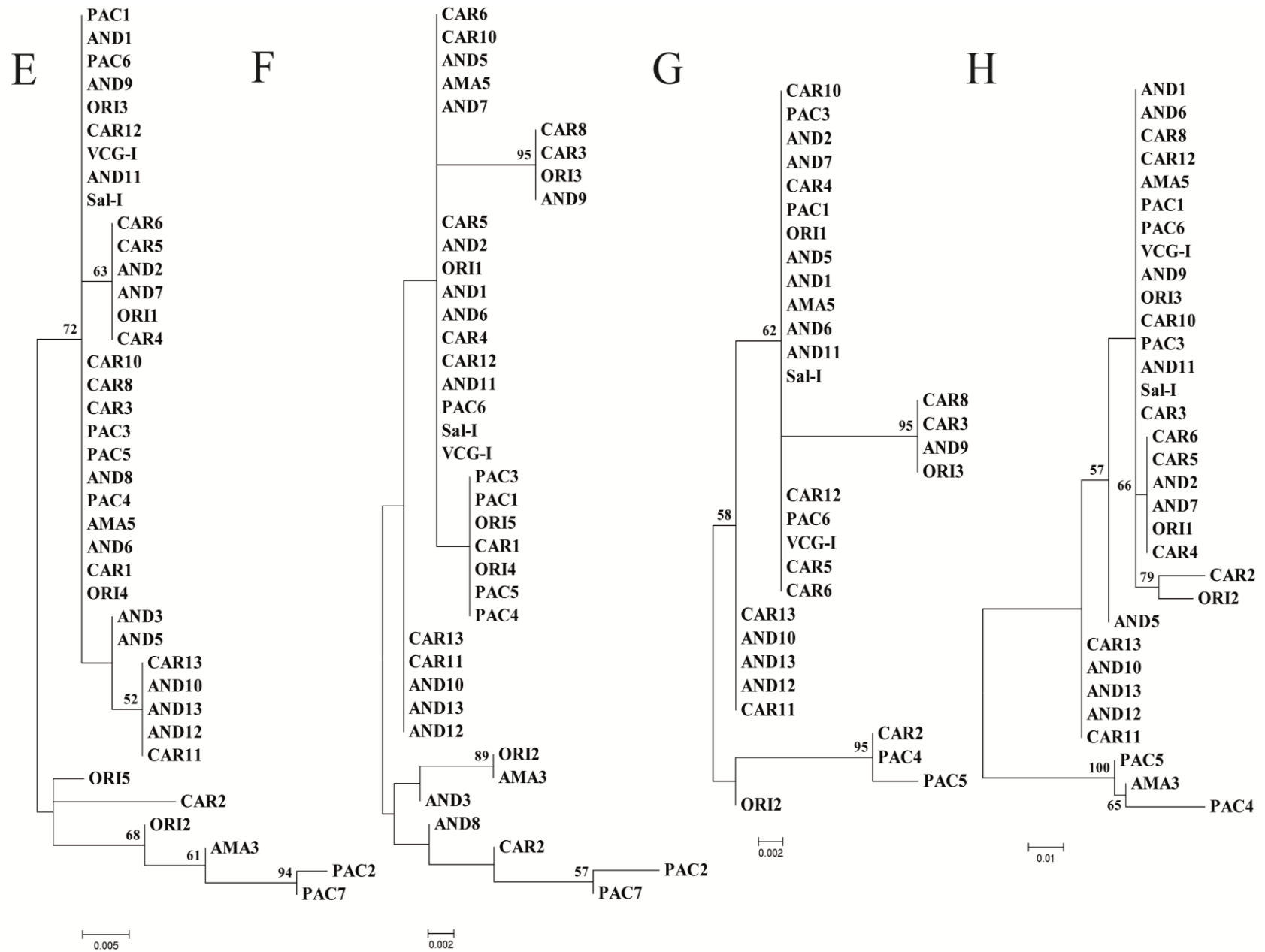

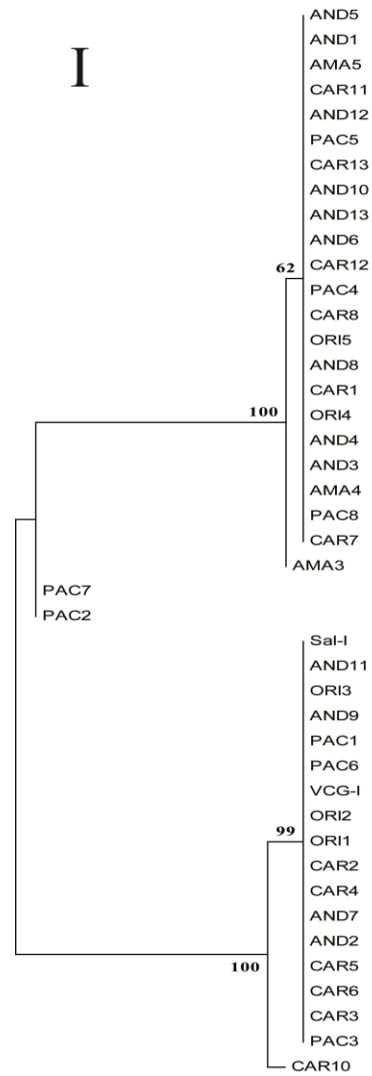

0.02

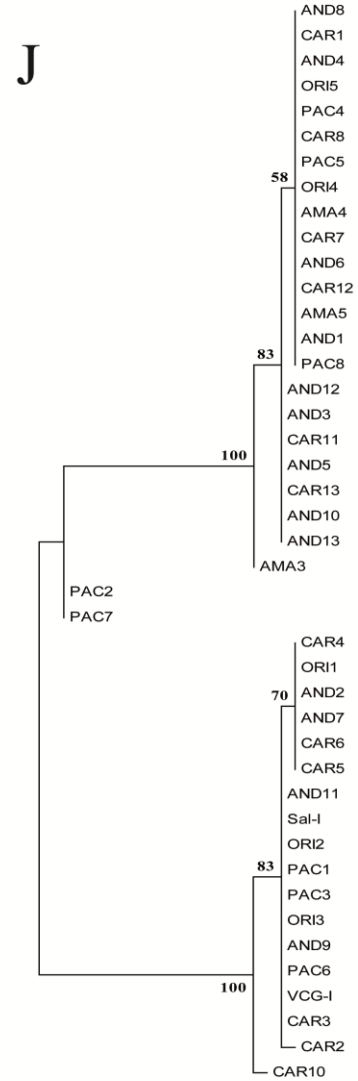

0.01

Supplement: Figure S10 — Alignment of deduced PvMSP-7C amino acid sequences. (PDF) [file pone.0045962.s010.pdf]

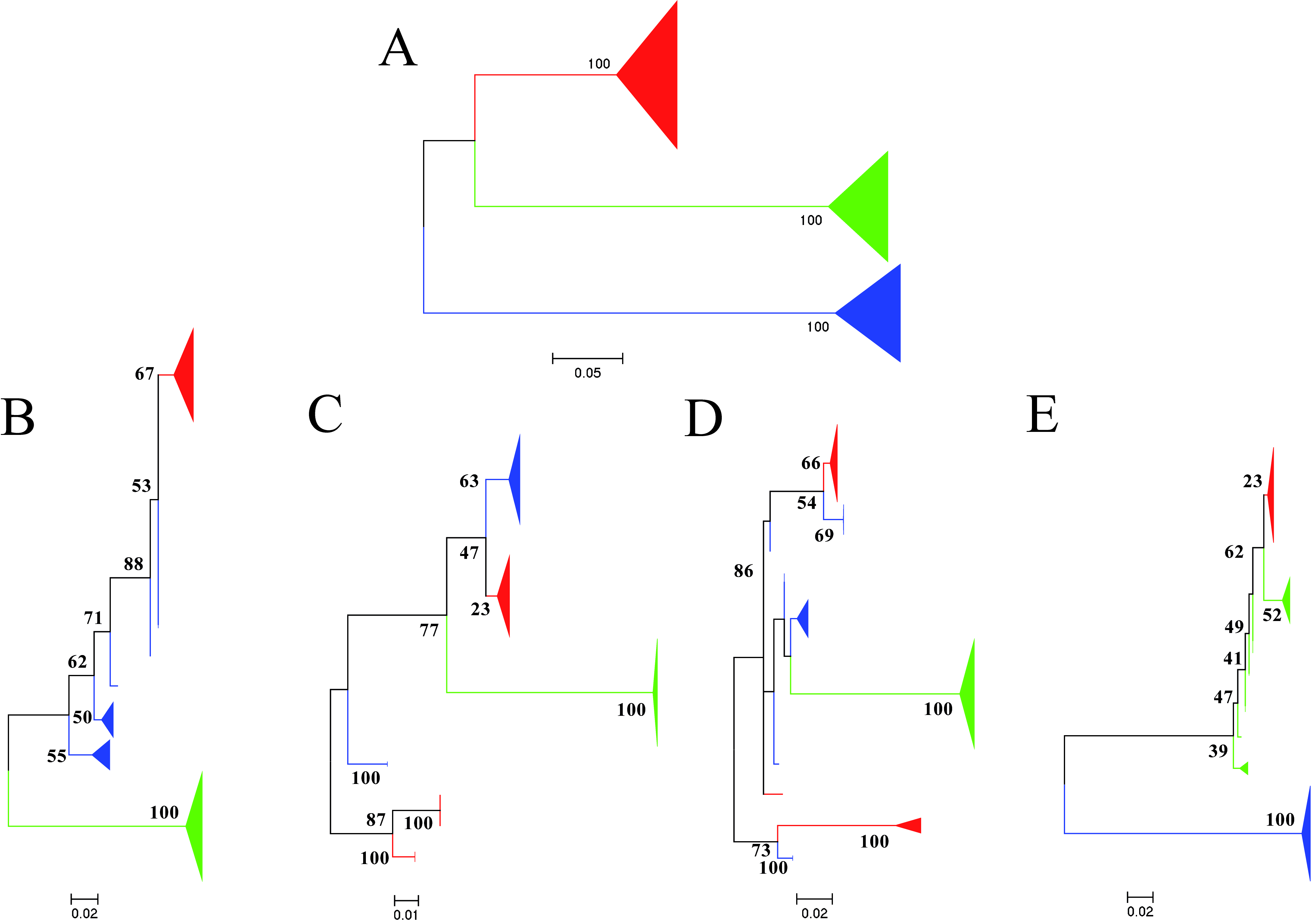

Supplement: Figure S11 — Alignment of deduced PvMSP-7H amino acid sequences. (TIF) [file pone.0045962.s011.tif]

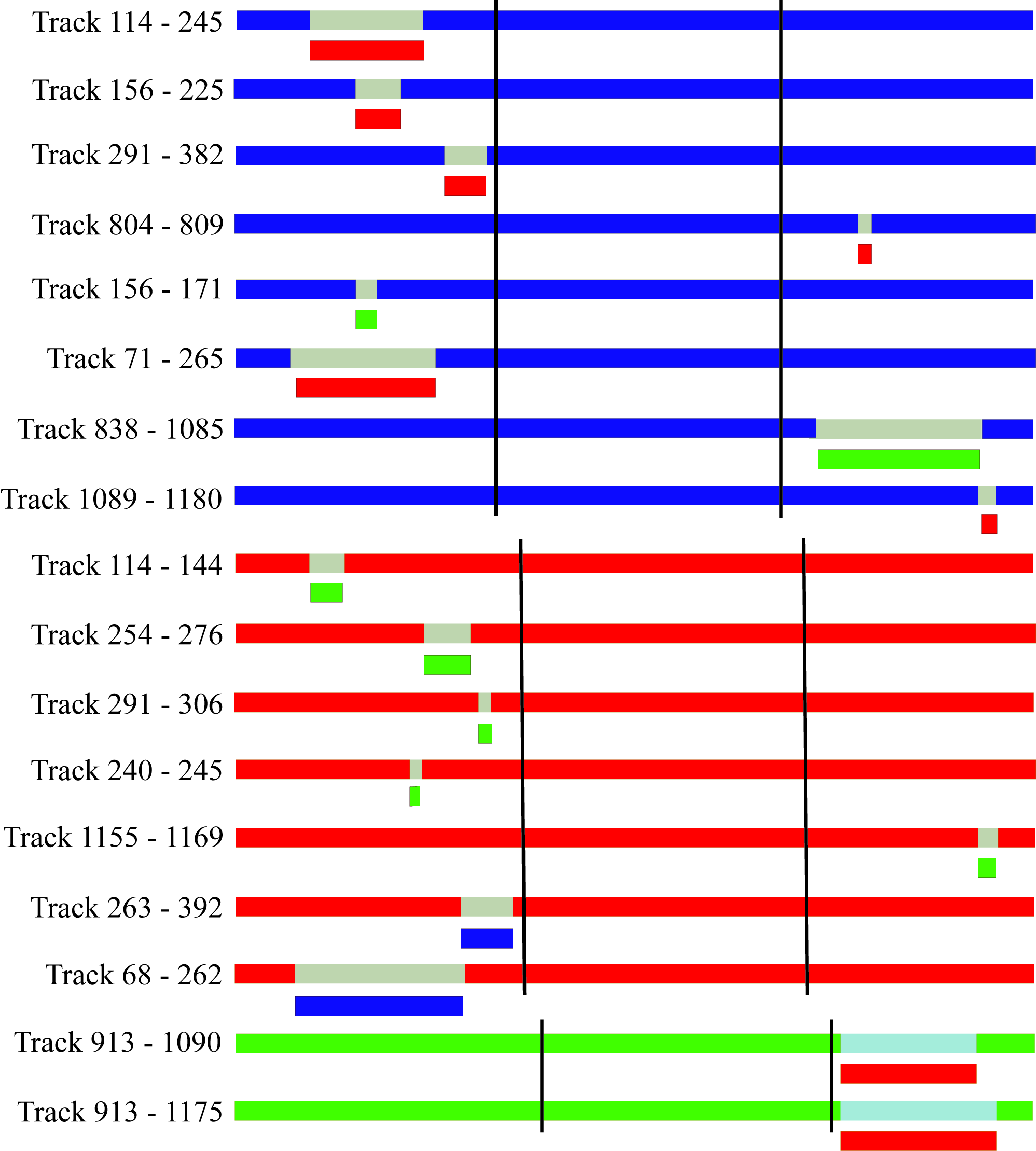

Supplement: Figure S12 — Alignment of deduced PvMSP-7C amino acid sequences. (TIF) [file pone.0045962.s012.tif]
