# Supplementary material for: Genetic Diversity and Selection in Three Plasmodium vivax Merozoite Surface Protein 7 (Pvmsp-7) Genes in a Colombian Population
Source: PLoS One. 2012 Sep 25;7(9):e45962. doi: 10.1371/journal.pone.0045962 (PMC3458108; doi:10.1371/journal.pone.0045962)
Supplement: Figure S2 — Sliding window analysis of Tajima D and Fu & Li D* and F* statistics along the Pvmsp-7C (A), Pvmsp-7H (B) and Pvmsp-7I (C) genes. Bars [Tajima’s D (blue), Fu & Li’s D* (red) and F* (green)] below each figure represent the regions where the tests showed a significant deviation from the neutral expectation. 5′-end (Pvmsp-7C: nucleotides 1–390, Pvmsp-7H: nucleotides 1–471, Pvmsp-7I: nucleotides 1–525), central (Pvmsp-7C: nucleotides 391–717, Pvmsp-7H: nucleotides 472–771, Pvmsp-7I: nucleotides 526–789) and 3′-end (Pvmsp-7C: nucleotides 718–1,191, Pvmsp-7H: nucleotides 772–1,200, Pvmsp-7I: nucleotides 790–1,188). (PDF) [file pone.0045962.s002.pdf]

```
                   *        20         *        40         *        60         *        80         *       100         *
Sal-I   : MNKTIVIFFLSFSFLISFHSVLSEKLGIQKKKKNLEQDAMHALMKKLESLYKLSATDNSEIFNKEMEALKKQIDQLQQHGGANEEESLGHLLESEAADDSSKKTIFGVDE : 110
AND6    : ------IFFLSFSFLISFHSVLSEKLGIQKKKKNLEQDAMHALMKKLESLYKLSATDNSEIFNKEMEALKKQIDQLQQHGGANEEESLGHLLESEAADDSSKKTIFGVDE : 104
ORI2    : ------IFFLSFSFLISFHSVLSEKLGIQKKKKNLEQDAMHALMKKLESLYKLSATDNSEIFNKEMEALKKQIDQLQQHGGANEEESLGHLLESEAADDSSKKTIFGVDE : 104
CAR8    : ------IFFLSFSFLISFHSVLSEKLGIQKKKKNLEQDAMHALMKKLESLYKLSATDNSEIFNKEMEALKKQIDQLQQHGGANEEESLGHLLESEAADDSSKKTIFGVDE : 104
AND7    : ------IFFLSFSFLISFHSVLSEKLGIQKKKKNLEQDAMHALMKKLESLYKLSATDNSEIFNKEMEALKKQIDQLQQHGGANEEESLGHLLESEAADDSSKKTIFGVDE : 104
CAR10   : ------IFFLSFSFLISFHSVLSEKLGIQKKKKNLEQDAMHALMKKLESLYKLSATDNSEIFNKEMEALKKQIDQLQQHGGANEEESLGHLLESEAADDSSKKTIFGVDE : 104
CAR11   : ------IFFLSFSFLISFHSVLSEKLGIQKKKKNLEQDAMHALMKKLESLYKLSATDNSEIFNKEMEALKKQIDQLQQHGGANEEESLGHLLESEAADDSSKKTIFGVDE : 104
AND4    : ------IFFLSFSFLISFHSVLSEKLGIQKKKKNLEQDAMHALMKKLESLYKLSATDNSEIFNKEMEALKKQIDHLQQHGGANEEESLGHLLESEAADDSSKKTIFGVDE : 104
AND5    : ------IFFLSFSFLISFHSVLSEKLGIQKKKKNLEQDAMHALMKKLESLYKLSATDNSEIFNKEMEALKKQIDHLQQHGGANEEESLGHLLESEAADDSSKKTIFGVDE : 104
PAC2    : ------IFFLSFSFLISFHSVLSEKLGIQKKKKNLEQDATHALMKKLESLYKLSATDNSEIFNKEIESLKKQIDQLHQHGGENEEESLGHLLESEAADDSGKKTIFGVDE : 104
PAC6    : ------IFFLSFSFLISFHSVLSEKLGIQKKKKNLEQDATHALMKKLESLYKLSATDNSEIFNKEIESLKKQIDQLHQHGGENEEESLGHLLESEAADDSGKKTIFGVDE : 104
AMA4    : ------IFFLSFSFLISFHSVLSEKLGIQKKKKNLEQDAMHALMKKLESLYKLSATDNSEIFNKEMEALKKQIDHLQQHGGANEEESLGHLLESEAADDSGKKTIFGVDE : 104
PAC3    : ------IFFLSFSFLISFHSVLSEKLGIQKKKKNLEQDAMHALMKKLESLYKLSATDNSEIFNKEMEALKKQIDQLQQHGGANEEESLGHLLESEAADDSGKKTIFGVDE : 104
AND1    : ------IFFLSFSFLISFHSVLSEKLGIQKKKKNLEQDAMHALMKKLESLYKLSATDNSEIFNKEMEALKKQIDQLQQHGGANEEESLGHLLESEAADDSGKKTIFGVDE : 104
CAR13   : ------IFFLSFSFLISFHSVLSEKLGIQKKKKNLEQDAMHALMKKLESLYKLSATDNSEIFNKEMEALKKQIDQLQQHGGANEEESLGHLLESEAADDSGKKTIFGVDE : 104
AMA5    : ------IFFLSFSFLISFHSVLSEKLGIQKKKKNLEQDAMHALMKKLESLYKLSATDNSEIFNKEMEALKKQIDQLQQHGGANEEESLGHLLESEAADDSGKKTIFGVDE : 104
CAR14   : ------IFFLSFSFLISFHSVLSEKLGIQKKKKNLEQDAMHALMKKLESLYKLSATDNSEIFNKEMEALKKQIDQLQQHGGANEEESLGHLLESEAADDSGKKTIFGVDE : 104
VCG-I   : ------IFFLSFSFLISFHSVLSEKLGIQKKKKNLEQDAMHALMKKLESLYKLSATDNSEIFNKEMEALKKQIDQLQQHGGANEEESLGHLLESEAADDSGKKTIFGVDE : 104
AMA2    : ------IFFLSFSFLISFHSVLSEKLGIQKKKKNLEQDAMHALMKKLESLYKLSATDNSEIFNKEMEALKKQIDQLQQHGGANEEESLGHLLESEAADDSGKKTIFGVDE : 104
CAR1    : ------IFFLSFSFLISFHSVLSEKLGIQKKKKNLEQDAMHALMKKLESLYKLSATDNSEIFNKEMEALKKQIDQLQQHGGANEEESLGHLLESEAADDSGKKTIFGVDE : 104
CAR9    : ------IFFLSFSFLISFHSVLSEKLGIQKKKKNLEQDAMHALMKKLESLYKLSATDNSEIFNKEMEALKKQIDQLQQHGGANEEESLGHLLESEAANESTKKTIFGVDE : 104
AND8    : ------IFFLSFSFLISFHSVLSEKLGIQKKKKNLEQDAMHALMKKLESLYKLSATDNSEIFNKEMEALKKQIDQLQQHGGANEEESLGHLLESEAANESTKKTIFGVDE : 104
ORI1    : ------IFFLSFSFLISFHSVLSEKLGIQKKKKNLEQDAMHALMKKLESLYKLSATDNSEIFNKEMEALKKQIDQLQQHGGANEEESLGHLLESEAANESTKKTIFGVDE : 104
AND2    : ------IFFLSFSFLISFHSVLSEKLGIQKKKKNLEQDAMHALMKKLESLYKLSATDNSEIFNKEMEALKKQIDQLQQHGGANEEESLGHLLESEAANESTKKTIFGVDE : 104
AND3    : ------IFFLSFSFLISFHSVLSEKLGIQKKKKNLEQDAMHALMKKLESLYKLSATDNSEIFNKEMEALKKQIDQLQQHGGANEEESLGHLLESEAANESTKKTIFGVDE : 104
CAR15   : ------IFFLSFSFLISFHSVLSEKLGIQKKKKNLEQDAMHALMKKLESLYKLSATDNSEIFNKEMEALKKQIDQLQQHGGANEEESLGHLLESEAANESTKKTIFGVDE : 104
CAR2    : ------IFFLSFSFLISFHSVLSEKLGIQKKKKNLEQDAMHALMKKLESLYKLSATDNSEIFNKEMEALKKQIDQLQQHGGANEEESLGHLLESEAANESTKKTIFGVDE : 104
CAR4    : ------IFFLSFSFLISFHSVLSEKLGIQKKKKNLEQDAMHALMKKLESLYKLSATDNSEIFNKEMEALKKQIDQLQQHGGANEEESLGHLLESEAANESTKKTIFGVDE : 104
CAR7    : ------IFFLSFSFLISFHSVLSEKLGIQKKKKNLEQDAMHALMKKLESLYKLSATDNSEIFNKEMEALKKQIDQLQQHGGANEEESLGHLLESEAANESTKKTIFGVDE : 104
CAR6    : ------IFFLSFSFLISFHSVLSEKLGIQKKKKNLEQDAMHALMKKLESLYKLSATDNSEIFNKEMEALKKQIDQLQQHGGANEEESLGHLLESEAANESTKKTIFGVDE : 104
CAR5    : ------IFFLSFSFLISFHSVLSEKLGIQKKKKNLEQDAMHALMKKLESLYKLSATDNSEIFNKEMEALKKQIDQLQQHGGANEEESLGHLLESEAANESTKKTIFGVDE : 104
ORI3    : ------IFFLSFSFLISFHSVLSEKLGIQKKKKNLEQDAMHALMKKLESLYKLSATDNSEIFNKEMEALKKQIDQLQQHGGANEEESLGHLLESEAANESTKKTIFGVDE : 104
CAR12   : ------IFFLSFSFLISFHSVLSEKLGIQKKKKNLEQDAMHALMKKLESLYKLSATDNSEIFNKEMEALKKQIDQLQQHGGANEEESLGHLLESEAADDSSKKTIFGVDE : 104
CAR3    : ------IFFLSFSFLISFHSVLSEKLGIQKKKKNLEQDAMHALMKKLESLYKLSATDNSEIFNKEMEALKKQIDQLQQHGGANEEESLGHLLESEAADDSSKKTIFGVDE : 104
PAC4    : ------IFFLSFSFLISFHSVLSEKLGIQKKKKNLEQDAMHALMKKLESLYKLSATDNSEIFNKEMEALKKQIDQLQQHGGANEEESLGHLLESEAADDSSKKTIFGVDE : 104
PAC8    : ------IFFLSFSFLISFHSVLSEKLGIQKKKKNLEQDAMHALMKKLESLYKLSATDNSEIFNKEMEALKKQIDQLQQHGGANEEESLGHLLESEAADDSSKKTIFGVDE : 104
ORI4    : ------IFFLSFSFLISFHSVLSEKLGIQKKKKNLEQDAMHALMKKLESLYKLSATDNSEIFNKEMEALKKQIDQLQQHGGANEEESLGHLLESEAANDSSKKTIFGVDE : 104
PAC5    : ------IFFLSFSFLISFHSVLSEKLGIQKKKKNLEQDAMHALMKKLESLYKLSATDNSEIFNKEMEALKKQIDQLQQHGGANEEESLGHLLESEAADDSSKKTIFGVDE : 104
                ifflsfsflisfhsvlseKLGIQKKKKNLEQDAmHALMKKLESLYKLSATDNsEIFNKE6EaLKKQIDqLqQHGGaNEEESLGHLLESEAA1 S KKTIFGVDE
```

```
                 120         *        140         *        160         *        180         *        200         *        220
Sal-I  : DDLDNYDGDFTGQSKGKIKGQADTAPSVKGDVSPPPNLPAAAASSPKETVPAGTSNGLVEADYVVLNTPDGNPRPVGPGGGSRPSASGPDAASNL-QN-Q--ATAEAGG- : 215
AND6   : DDLDNYDGDFTGQSKGKIKGQADTAPSVKGDVSPPPNLPAAAASSPKETVPAGTSNGLVEADYVVLNTPDGNPRPVGPGGGSRPSASGPDAASNL-QN-Q--ATAEAGG- : 209
ORI2   : DDLDNYDGDFTGQSKGKIKGQADTAPSVKGDVSPPPNLPAAAASSPKETVPAGTSNGLVEADYVVLNTPDGNPRPVGPGGGSRPSASGPDAASNL-QN-Q--ATAEAGG- : 209
CAR8   : DDLDNYDGDFTGQSKGKIKGQADTAPSVKGDVSPPPNLPAAAASSPKETVPAGTSNGLVEADYVVLNTPDGNPRPVGPGGGSRPSASGPDAASNL-QN-Q--ATAEAGG- : 209
AND7   : DDLDNYDGDFTGQSKGKIKGQADTAPSVKGDVSPPPNLPAAAASSPKETVPAGTSNGLVEADYVVLNTPDGNPRPVGPGGGSRPSASGPDAASNL-QN-Q--ATAEAGG- : 209
CAR10  : DDLDNYDGDFTGQSKGKIKGQADTAPSVKGDVSPPPNLPAAAASSPKETVPAGTSNGLVEADYVVLNTPDGNPRPVGPGGGSRPSASGPDAASNL-QN-Q--ATAEAGG- : 209
CAR11  : DDLDNYDGDFTGQSKGKIKGQADTAPSVKGDVSPPPNLPAAAASSPKETVPAGTSNGLVEADYVVLNTPDGNPRPVGPGGGSRPSASGPDAASNL-QN-Q--ATAEAGG- : 209
AND4   : DDLDNYDGDFTGQSKGKIKGQADTAPSVKGDVSPPPNLPAAAASSPKETVPAGTSNGLVEADYVVLNTPDGNPGPVGPGGGSRPSASGPDAASNL-QN-P--ATAEAGG- : 209
AND5   : DDLDNYDGDFTGQSKGKIKGQADTAPSVKGDVSPPPNLPAAAASSPKETVPAGTSNGLVEADYVVLNTPDGNPGPVGPGGGSRPSASGPDAASNL-QN-P--ATAEAGG- : 209
PAC2   : DDLDNYDADFTGQSKGKIKGQADAGQTVESNVSPRPASSAADNSLPKKTTPAGTSSGVVEVRYVNPNSPDS-PSDALSGGGSRPSSQGPGTSPNV-QN-Q------SG-- : 203
PAC6   : DDLDNYDADFTGQSKGKIKGQADAGQTVESNVSPRPASSAADNSLPKKTTPAGTSSGVVEVRYVNPNSPDS-PSDALSGGGSRPSSQGPGTSPNV-QN-Q------SG-- : 203
AMA4   : DDLDNYDADFTGQSKGKIKGQADAGQTVESNVSPRPASSAADNSLPKKTTPAGTSSGVVEVRYVNPNSPDS-PSDALSGGGSRPSSQGPGTSPNV-QN-Q------SG-- : 203
PAC3   : DDLDNYDADFTGQSKGKIKGQADAGQTVESNVSPRPASSAADNSLPKETTPAGTSSGVVEVRYVNPNSPDS-PSDALSGGGSRPSSQGPGTSPNV-QN-Q------SG-- : 203
AND1   : DDLDNYDADFTGQSKGKIKGQADAGQTVESNVSPRPASSAADNSLPKETTPAGTSSGVVEVRYVNPNSPDS-PSDALSGGGSRPSSQGPGTSPNV-QN-Q------SG-- : 203
CAR13  : DDLDNYDADFTGQSKGKIKGQADAGQTVESNVSPRPASSAADNSLPKETTPAGTSSGVVEVRYVNPNSPDS-PSDALSGGGSRPSSQGPGTSPNV-QN-Q------SG-- : 203
AMA5   : DDLDNYDADFTGQSKGKIKGQADAGQTVESNVSPRPASSAADNSLPKETTPAGTSSGVVEVRYVNPNSPDS-PSDALSGGGSRPSSQGPGTSPNV-QN-Q------SG-- : 203
CAR14  : DDLDNYDADFTGQSKGKIKGQADAGQTVESNVSPRPASSAADNSLPKETTPAGTSSGVVEVRYVNPNSPDS-PSDALSGGGSRPSSQGPGTSPNV-QN-Q------SG-- : 203
VCG-I  : DDLDNYDADFTGQSKGKIKGQADAGQTVESNVSPRAASSAAGNSLPKETTPAGTSSGVVEVRYVNPNSPDS-PSDALSGGGSRPSSQGPGTSPNV-QN-Q------SG-- : 203
AMA2   : DDLDNYDADFTGQSKGKIKGQADAGQTVESNVSPRPASSAADNSLPKETTPAGTSSGVVEVRYVNPNSPDS-PSDALSGGGSRPSSQGPGTSPNV-QN-Q------SG-- : 203
CAR1   : DDLDNYDADFTGQSKGKIKGQADAGQTVESNVSPRPASSAADNSLPKETTPAGTSSGVVEVRYVNPNSPDS-PSDALSGGGSRPSSQGPGTSPNV-QN-Q------SG-- : 203
CAR9   : DDLDNYDGDFTGQSKGKIKGQADASQSVKGADTPGSKLPAAADSPPRGTAADGRNSHVVEIGYINRNSADSSPLAAGSGGDSTLSASGPGSASQITQPSPSSPGGVPGNT : 214
AND8   : DDLDNYDGDFTGQSKGKIKGQADASQSVKGADTPGSKLPAAADSPPRGTAADGRNSHVVEIGYINRNSADSSPLAAGSGGDSTLSASGPGSASQITQPSPSSPGGVPGNT : 214
ORI1   : DDLDNYDGDFTGQSKGKIKGQADASQSVKGADTPGSKLPAAADSPPRGTAADGRNSHVVEIGYINRNSADSSPLAAGSGGDSTLSASGPGSASQITQPSPSSPGGVPGNT : 214
AND2   : DDLDNYDGDFTGQSKGKIKGQADASQSVKGADTPGSKLPAAADSPPRGTAADGRNSHVVEIGYINRNSADSSPLAAGSGGDSTLSASGPGSASQITQPSPSSPGGVPGNT : 214
AND3   : DDLDNYDGDFTGQSKGKIKGQADASQSVKGADTPGSKLPAAADSPPRGTAADGRNSHVVEIGYINRNSADSSPLAAGSGGDSTLSASGPGSASQITQPSPSSPGGVPGNT : 214
CAR15  : DDLDNYDGDFTGQSKGKIKGQADASQSVKGADTPGSKLPAAADSPPRGTAADGRNSHVVEIGYINRNSADSSPLAAGSGGDSTLSASGPGSASQITQPSPSSPGGVPGNT : 214
CAR2   : DDLDNYDGDFTGQSKGKIKGQADASQSVKGADTPGSKLPAAADSPPRGTAADGRNSHVVEIGYINRNSADSSPLAAGSGGDSTLSASGPGSASQITQPSPSSPGGVPGNT : 214
CAR4   : DDLDNYDGDFTGQSKGKIKGQADASQSVKGADTPGSKLPAAADSPPRGTAADGRNSHVVEIGYINRNSADSSPLAAGSGGDSTLSASGPGSASQITQPSPSSPGGVPGNT : 214
CAR7   : DDLDNYDGDFTGQSKGKIKGQADASQSVKGADTPGSKLPAAADSPPRGTAADGRNSHVVEIGYINRNSADSSPLAAGSGGDSTLSASGPGSASQITQPSPSSPGGVPGNT : 214
CAR6   : DDLDNYDGDFTGQSKGKIKGQADASQSVKGADTPGSKLPAAADSPPRGTAADGRNSHVVEIGYINRNSADSSPLAAGSGGDSTLSASGPGSASQITQPSPSSPGGVPGNT : 214
CAR5   : DDLDNYDGDFTGQSKGKIKGQADASQSVKGADTPGSKLPAAADSPPRGTAADGRNSHVVEIGYINRNSADSSPLAAGSGGDSTLSASGPGSASQITQPSPSSPGGVPGNT : 214
ORI3   : DDLDNYDGDFTGQSKGKIKGQADATQSVKGADTPGSKLPAAADSPPKGTAADGRNSHVVEIGYINRNSPDSSPLAAGSGGDSTLSASGPGSASQITQPSPSSPGGVPGNT : 214
CAR12  : DDLDNYDGDFTGQSKGKIKGQADASQSVKGADTPGSKLPAAADSPPRGTAADGRNSHVVEIGYINRNSADSSPLAAGSGGDSTLSASGPGSASQITQPSPSSPGGVPGNT : 214
CAR3   : DDLDNYDGDFTGQSKGKIKGQADAAPSVKGAVTPASKLPAAADSPPRGTAADGRNSHVVEIGYINRNSADSSPLAAGSGGDSTLSASGPGSASQITQPSPSSPGGVPGNT : 214
PAC4   : DDLDNYDGDFTGQSKGKIKGQADASQSVKGADTPGSKLPAAADSPPRGTAADGRNSHVVEIGYINRNSADSSPLAAGSGGDSTLSASGPGSASQITQPSPSSPGGVPGNT : 214
PAC8   : DDLDNYDGDFTGQSKGKIKGQADASQSVKGADTPGSKLPAAADSPPKETAPEGTNSHVVEIGYINRNSPDSSPLAAGSGGDSTLSASGPGSASQITQPSPSSPAGVPGNT : 214
ORI4   : DDLDNYDGDFTGQSKGKIKGQADASQSVKGDDTPRSNLPAAADSSPKETAAAGTSSHVVEIGYVNRNSADSSPRAVGSGGDSRPSASGPGSASQITQPTPSSPGGVPGNT : 214
PAC5   : DDLDNYDGDFTGQSKGKIKGQADAGQSVKGDVSPRPNLPAAADSSPKETAPAGTSSGVVEVRYINPNSPDSSPLAAGSGGDSTLSASGPGSASQITQPSPSSPGGVPGNT : 214
         DDLDNYD DFTGQSKGKIKGQAD   3V    3P      AA   S P4 T    G     6VE   Y6   N3 D   P      GG S   S   GP      6 Q          G
```

```
                         *        240         *        260         *        280         *        300         *        320         *
Sal-I  : --STN--TQGSQTGGVSTTPGANQQAGSTPPGAATAQTESEREPNVKYLDKLYDDVLATQDSANGIDVPPFHSKYNDFRKKYEFKMNDSEYKIMKNLFDVSFKKEGQQSS : 321
AND6   : --STN--TQGSQTGGVSTTPGANQQAGSTPPGAATAQTEGEREPNVKYLDKLYDDVLATQDSANGIDVPPFHSKYNDFRKKYEFKMNDSEYKIMKNLFDVSFKKEGQQSS : 315
ORI2   : --STN--TQGSQTGGVSTTPGANQQAGSTPPGAATAQTEGEREPNVKYLDKLYDDVLATQDSANGIDVPPFHSKYNDFRKKYEFKMNDSEYKIMKNLFDVSFKKEGQQSS : 315
CAR8   : --STN--TQGSQTGGVSTTPGANQQAGSTPPGAATAQTEGEREPNVKYLDKLYDDVLATQDSANGIDVPPFHSKYNDFRKKYEFKMNDSEYKIMKNLFDVSFKKEGQQSS : 315
AND7   : --STN--TQGSQTGGVSTTPGANQQAGSTPPGAATAQTEGEREPNVKYLDKLYDDVLATQDSANGIDVPPFHSKYNDFRKKYEFKMNDSEYKIMKNLFDVSFKKEGQQSS : 315
CAR10  : --STN--TQGSQTGGVSTTPGANQQAGSTPPGASTAQTEGEREPNVKYLDKLYDDVLATQDSANGIDVPPFHSKYNDFRKKYEFKMNDSEYKIMKNLFDVSFKKEGQQSS : 315
CAR11  : --STN--TQGSQTGGVSTTPGANQQAGSTPPGASTAQTEGEREPNVKYLDKLYDDVLATQDSANGIDVPPFHSKYNDFRKKYEFKMNDSEYKIMKNLFDVSFKKEGQQSS : 315
AND4   : --STN--TQGSQTGGVSTTPGANQQAGSTPPGAATAQTEGEREPNVKYLDKLYDDVLATQDSANGIDVPPFHSKYNDFRKKYEFKMNDSEYKIMKNLFDVSFKKEGQQSS : 315
AND5   : --STN--TQGSQTGGVSTTPGANQQAGSTPPGAATAQTEGEREPNVKYLDKLYDDVLATQDSANGIDVPPFHSKYNDFRKKYEFKMNDSEYKIMKNLFDVSFKKEGQQSS : 315
PAC2   : --NSQ---AAAEAGGSTNTPGANQQAGSTPPGAATAQTEGEREPNVKYLDKLYDDVLATQDSANGIDVPPFHSKYNDFRKKYEFKMNDSEYKIMKNLFDVSFKKEGQQSS : 308
PAC6   : --NSQ---AAAEAGGSTNTPGANQQAGSTPPGAATAQTEGEREPNVKYLDKLYDDVLATQDSANGIDVPPFHSKYNDFRKKYEFKMNDSEYKIMKNLFDVSFKKEGQQSS : 308
AMA4   : --NSQ---AAAEAGGSTNTPGANQQAGSTPPGAATAQTEGEREPNVKYLDKLYDDVLATQDSANGIDVPPFHSKYNDFRKKYEFKMNDSEYKIMKNLFDVSFKKEGQQSS : 308
PAC3   : --NSQ---AAAEAGGSTNTPGANQQAGSTPPGAATAQTEGEREPNVKYLDKLYDDVLATQDSANGIDVPPFHSKYNDFRKKYEFKMNDSEYKIMKNLFDVSFKKEGQQSS : 308
AND1   : --NSQ---AATEAGGSTNTPGANQQAGSTPPGAATAQTEGEREPNVKYLDKLYDDVLATQDSANGIDVPPFHSKYNDFRKKYEFKMNDSEYKIMKNLFDVSFKKEGQQSS : 308
CAR13  : --NSQ---AAAEAGGSTNTPGANQQAGSTPPGAATAQTEGEREPNVKYLDKLYDDVLATQDSANGIDVPPFHSKYNDFRKKYEFKMNDSEYKIMKNLFDVSFKKEGQQSS : 308
AMA5   : --NSQ---AAAEAGGSTNTPGANQQAGSTPPGAATAQTEGEREPNVKYLDKLYDDVLATQDSANGIDVPPFHSKYNDFRKKYEFKMNDSEYKIMKNLFDVSFKKEGQQSS : 308
CAR14  : --NSQ---AAAEAGGSTNTPGANQQAGSTPPGAATAQTEGEREPNVKYLDKLYDDVLATQDSANGIDVPPFHSKYNDFRKKYEFKMNDSEYKIMKNLFDVSFKKEGQQSS : 308
VCG-I  : --NSQ---AAAEAGGSTNTPGANQQAGSTPPGAATAQTEGEREPNVKYLDKLYDDVLATQDSANGIDVPPFHSKYNDFRKKYEFKMNDSEYKIMKNLFDVSFKKEGQQSS : 308
AMA2   : --NSQ---AAAEAGGSTNTPGANQQAGSTPPGAATAQTEGEREPNVKYLDKLYDDVLATQDSANGIDVPPFHSKYNDFRKKYEFKMNDSEYKIMKNLFDVSFKKEGQQSS : 308
CAR1   : --NSQ---AAAEAGGSTNTPGANQQAGSTPPGAATAQTEGEREPNVKYLDKLYDDVLATQDSANGIDVPPFHSKYNDFRKKYEFKMNDSEYKIMKNLFDVSFKKEGQQSS : 308
CAR9   : LTNVQPQTPVGAGGG-TNTPGANQQAGSTPPGAATAQTEGEREPNVKYLDKLYDDVLATQDSANGIDVPPFHSKYNDFRKKYEFKMNDSEYKIMKNLFDVSFKKEGQQSS : 323
AND8   : LTNVQPQTPVGAGGG-TNTPGANQQAGSTPPGAATAQTEGEREPNVKYLDKLYDDVLATQDSANGIDVPPFHSKYNDFRKKYEFKMNDSEYKIMKNLFDVSFKKEGQQSS : 323
ORI1   : LTNVQPQTPVGAGGG-TNTPGANQQAGSTPPGAATAQTEGEREPNVKYLDKLYDDVLATQDSANGIDVPPFHSKYNDFRKKYEFKMNDSEYKIMKNLFDVSFKKEGQQSS : 323
AND2   : LTNVQPQTPVGAGGG-TNTPGANQQAGSTPPGAATAQTEGEREPNVKYLDKLYDDVLATQDSANGIDVPPFHSKYNDFRKKYEFKMNDSEYKIMKNLFDVSFKKEGQQSS : 323
AND3   : LTNVQPQTPVGAGGG-TNTPGANQQAGSTPPGAATAQTEGEREPNVKYLDKLYDDVLATQDSANGIDVPPFHSKYNDFRKKYEFKMNDSEYKIMKNLFDVSFKKEGQQSS : 323
CAR15  : LTNVQPQTPVGAGGG-TNTPGANQQAGSTPPGAATAQTEGEREPNVKYLDKLYDDVLATQDSANGIDVPPFHSKYNDFRKKYEFKMNDSEYKIMKNLFDVSFKKEGQQSS : 323
CAR2   : LTNVQPQTPVGAGGG-TNTPGANQQAGSTPPGAATAQTEGEREPNVKYLDKLYDDVLATQDSANGIDVPPFHSKYNDFRKKYEFKMNDSEYKIMKNLFDVSFKKEGQQSS : 323
CAR4   : LTNVQPQTPVGAGGG-TNTPGANQQAGSTPPGAATAQTEGEREPNVKYLDKLYDDVLATQDSANGIDVPPFHSKYNDFRKKYEFKMNDSEYKIMKNLFDVSFKKEGQQSS : 323
CAR7   : LTNVQPQTPVGAGGG-TNTPGANQQAGSTPPGAATAQTEGEREPNVKYLDKLYDDVLATQDSANGIDVPPFHSKYNDFRKKYEFKMNDSEYKIMKNLFDVSFKKEGQQSS : 323
CAR6   : LTNVQPQTPVGAGGG-TNTPGANQQAGSTPPGAATAQTEGEREPNVKYLDKLYDDVLATQDSANGIDVPPFHSKYNDFRKKYEFKMNDSEYKIMKNLFDVSFKKEGQQSS : 323
CAR5   : LTNVQPQTPVGAGGG-TNTPGANQQAGSTPPGAATAQTEGEREPNVKYLDKLYDDVLATQDSANGIDVPPFHSKYNDFRKKYEFKMNDSEYKIMKNLFDVSFKKEGQQSS : 323
ORI3   : LTNVQPQTPVGAGGG-TNTPGANQQAGSTPPGAATAQTEGEREPNVKYLDKLYDDVLATQDSANGIDVPPFHSKYNDFRKKYEFKMNDSEYKIMKNLFDVSFKKEGQQSS : 323
CAR12  : LTNVQPQTPVGAGGS-TNTPGANQQAGSTPPGAATAQTEGEREPNVKYLDKLYDDVLATQDSANGIDVPPFHSKYNDFRKKYEFKMNDSEYKIMKNLFDVSFKKEGQQSS : 323
CAR3   : LTNVQPQTPVGAGGS-TNTPGANQQAGSTPPGAATAQTEGEREPNVKYLDKLYDDVLATQDSANGIDVPPFHSKYNDFRKKYEFKMNDSEYKIMKNLFDVSFKKEGQQSS : 323
PAC4   : LTNVQPQTPVGAGGG-TNTPGANQQAGSTPPGAATAQTEGEREPNVKYLDKLYDDVLATQDSANGIDVPPFHSKYNDFRKKYEFKMNDSEYKIMKNLFDVSFKKEGQQSS : 323
PAC8   : LTNVQPQTPVGAGGG-TNTPGANQQAGSTPPGAATAQTEGEREPNVKYLDKLYDDVLATQDSANGIDVPPFHSKYNDFRKKYEFKMNDSEYKIMKNLFDVSFKKEGQQSS : 323
ORI4   : LTNVQPQTPVGAGGG-TNTPGANQQAGSTPPGAATAQTEGEREPNVKYLDKLYDDVLATQDSANGIDVPPFHSKYNDFRKKYEFKMNDSEYKIMKNLFDVSFKKEGQQSS : 323
PAC5   : LTNVQPQTPVGAGGG-TNTPGANQQAGSTPPGAATAQTEGEREPNVKYLDKLYDDVLATQDSANGIDVPPFHSKYNDFRKKYEFKMNDSEYKIMKNLFDVSFKKEGQQSS : 323
                    Gg 3 TPGANQQAGSTPPGAaTAQTEgEREPNVKYLDKLYDDVLATQDSANGIDVPPFHSKYNDFRKKYEFKMNDSEYKIMKNLFDVSFKKEGQQSS
```

```
                    340         *        360         *        380         *
Sal-I   : AACLVNVFKKVLDDEHLQKEFDNFVHGFYGFAKRHNYL-RGERMANENLYKDIFKNVVNLLNTIEVV : 387
AND6    : AACLVNVFKKVLDDEHLQKEFDNFVHGFYGFAKRHNYL-RGERMANENLYKDIFKNVVN-------- : 373
ORI2    : AACLVNVFKKVLDDEHLQKEFDNFVHGFYGFAKRHNYL-RGERMANENLYKDIFKNVVN-------- : 373
CAR8    : AACLVNVFKKVLDDEHLQKEFDNFVHGFYGFAKRHNYL-RGERMANENLYKDIFKNVVN-------- : 373
AND7    : AACLVNVFKKVLDDEHLQKEFDNFVHGFYGFAKRHNYL-RGERMANENLYKDIFKNVVN-------- : 373
CAR10   : AACLVNVFKKVLDDEHLQKEFDNFVHGFYGFAKRHNYL-RGERMANENLYKDIFKNVVN-------- : 373
CAR11   : AACLVNVFKKVLDDEHLQKEFDNFVHGFYGFAKRHNYL-RGERMANENLYKDIFKNVVN-------- : 373
AND4    : AACLVNVFKKVLDDEHLQKEFDNFVHGFYGFAKRHNYL-RGERMANENLYKDIFKNVVN-------- : 373
AND5    : AACLVNVFKKVLDDEHLQKEFDNFVHGFYGFAKRHNYL-RGERMANENLYKDIFKNVVN-------- : 373
PAC2    : AACLVNVFKKVLDDEHLQKEFDNFVHGFYGFAKRHNYL-RGERMANENLYKDIFKNVVN-------- : 366
PAC6    : AACLVNVFKKVLDDEHLQKEFDNFVHGFYGFAKRHNYL-RGERMANENLYKDIFKNVVN-------- : 366
AMA4    : AACLVNVFKKVLDDEHLQKEFDNFVHGFYGFAKRHNYLERGERMANENLYKDIFKNVVN-------- : 367
PAC3    : AACLVNVFKKVLDDEHLQKEFDNFVHGFYGFAKRHNYL-RGERMANENLYKDIFKNVVN-------- : 366
AND1    : AACLVNVFKKVLDDEHLQKEFDNFVHGFYGFAKRHNYL-RGERMANENLYKDIFKNVVN-------- : 366
CAR13   : AACLVNVFKKVLDDEHLQKEFDNFVHGFYGFAKRHNYL-RGERMANENLYKDIFKNVVN-------- : 366
AMA5    : AACLVNVFKKVLDDEHLQKEFDNFVHGFYGFAKRHNYL-RGERMANENLYKDIFKNVVN-------- : 366
CAR14   : AACLVNVFKKVLDDEHLQKEFDNFVHGFYGFAKRHNYL-RGERMANENLYKDIFKNVVN-------- : 366
VCG-I   : AACLVNVFKKVLDDEHLQKEFDNFVHGFYGFAKRHNYL-RGERMANENLYKDIFKNVVN-------- : 366
AMA2    : AACLVNVFKKVLDDEHLQKEFDNFVHGFYGFAKRHNYL-RGERMANENLYKDIFKNVVN-------- : 366
CAR1    : AACLVNVFKKVLDDEHLQKEFDNFVHGFYGFAKRHNYL-RGERMANENLYKDIFKNVVN-------- : 366
CAR9    : AACLVNVFKKVLDDEHLQKEFDNFVHGFYGFAKRHNYL-RGERMANENLYKDIFKNVVN-------- : 381
AND8    : AACLVNVFKKVLDDEHLQKEFDNFVHGFYGFAKRHNYL-RGERMANENLYKDIFKNVVN-------- : 381
ORI1    : AACLVNVFKKVLDDEHLQKEFDNFVHGFYGFAKRHNYL-RGERMANENLYKDIFKNVVN-------- : 381
AND2    : AACLVNVFKKVLDDEHLQKEFDNFVHGFYGFAKRHNYL-RGERMANENLYKDIFKNVVN-------- : 381
AND3    : AACLVNVFKKVLDDEHLQKEFDNFVHGFYGFAKRHNYL-RGERMANENLYKDIFKNVVN-------- : 381
CAR15   : AACLVNVFKKVLDDEHLQKEFDNFVHGFYGFAKRHNYL-RGERMANENLYKDIFKNVVN-------- : 381
CAR2    : AACLVNVFKKVLDDEHLQKEFDNFVHGFYGFAKRHNYL-RGERMANENLYKDIFKNVVN-------- : 381
CAR4    : AACLVNVFKKVLDDEHLQKEFDNFVHGFYGFAKRHNYL-RGERMANENLYKDIFKNVVN-------- : 381
CAR7    : AACLVNVFKKVLDDEHLQKEFDNFVHGFYGFAKRHNYL-RGERMANENLYKDIFKNVVN-------- : 381
CAR6    : AACLVNVFKKVLDDEHLQKEFDNFVHGFYGFAKRHNYL-RGERMANENLYKDIFKNVVN-------- : 381
CAR5    : AACLVNVFKKVLDDEHLQKEFDNFVHGFYGFAKRHNYL-RGERMANENLYKDIFKNVVN-------- : 381
ORI3    : AACLVNVFKKVLDDEHLQKEFDNFVHGFYGFAKRHNYL-RGERMANENLYKDIFKNVVN-------- : 381
CAR12   : AACLVNVFKKVLDDEHLQKEFDNFVHGFYGFAKRHNYL-RGERMANENLYKDIFKNVVN-------- : 381
CAR3    : AACLVNVFKKVLDDEHLQKEFDNFVHGFYGFAKRHNYL-RGERMANENLYKDIFKNVVN-------- : 381
PAC4    : AACLVNVFKKVLDDEHLQKEFDNFVHGFYGFAKRHNYL-RGERMANENLYKDIFKNVVN-------- : 381
PAC8    : AACLVNVFKKVLDDEHLQKEFDNFVHGFYGFAKRHNYL-RGERMANENLYKDIFKNVVN-------- : 381
ORI4    : AACLVNVFKKVLDDEHLQKEFDNFVHGFYGFAKRHNYL-RGERMANENLYKDIFKNVVN-------- : 381
PAC5    : AACLVNVFKKVLDDEHLQKEFDNFVHGFYGFAKRHNYL-RGERMANENLYKDIFKNVVN-------- : 381
          AACLVNVFKKVlDDEHLQKEFDNFVHGFYGFAKRHNYL RGERMANENLYKDIFKNVVN
```
