# Supplementary material for: Genetic Diversity and Selection in Three Plasmodium vivax Merozoite Surface Protein 7 (Pvmsp-7) Genes in a Colombian Population
Source: PLoS One. 2012 Sep 25;7(9):e45962. doi: 10.1371/journal.pone.0045962 (PMC3458108; doi:10.1371/journal.pone.0045962)
Supplement: Figure S3 — Sliding window analysis for ω rates (dN/dS) throughout the Pvmsp-7C (Blue), Pvmsp-7H (Red) and Pvmsp-7I (Green) genes. Discontinuity of the curves is due to gaps within the alignments which were not considered for the analysis. 5′-end (Pvmsp-7C: nucleotides 1–390, Pvmsp-7H: nucleotides 1–471, Pvmsp-7I: nucleotides 1–525), central (Pvmsp-7C: nucleotides 391–717, Pvmsp-7H: nucleotides 472–771, Pvmsp-7I: nucleotides 526–789) and 3′-end (Pvmsp-7C: nucleotides 718–1,191, Pvmsp-7H: nucleotides 772–1,200, Pvmsp-7I: nucleotides 790–1,188). (PDF) [file pone.0045962.s003.pdf]

```
                   *        20         *        40         *        60         *        80         *       100
Sal-I  : MKKKIVLFGSLFVLLSCSTVSSEKLGIQKKKKNLEQDATHALMKKLESLYKLSATDNSEIFNKEIESLKKQIDQLHQHGGENEGESLGHLLESEAANESTKKTIFGVD : 108
PAC2   : MKKKIVLFGSLFVLLSCSTVSSEKLGIQKKKKNLEQDAMHALMKKLESLYKLSATDNSEIFNKEMEALKKQIDQLQQHGGANEEESLGHLLESEDADDSGKKTIFGVD : 108
AND4   : MKKKIVLFGSLFVLLSCSTVSSEKLGIQKKKKNLEQDAMHALMKKLESLYKLSATDNSEIFNKEMEALKKQIDQLQQHGGANEEESLGHLLESEAANESTKKTIFGVD : 108
AND1   : MKKKIVLFGSLFVLLSCSTVSSEKLGIQKKKKNLEQDAMHALMKKLESLYKLSATDNSEIFNKEMEALKKQIDQLHQHGGANEEESLGHLLESEAANESTKKTIFGVD : 108
PAC3   : MKKKIVLFGSLFVLLSCSTVSSEKLGIQKKKKNLEQDATHALMKKLESLYKLSATDNSEIFNKEIESLKKQIDQLHQHGGENEGESLGHLLESEAANESTKKTIFGVD : 108
CAR5   : MKKKIVLFGSLFVLLSCSTVSSEKLGIQKKKKNLEQDATHALMKKLESLYKLSATDNSEIFNKEIESLKKQIDQLHQHGGENEGESLGHLLESEAANESAKKTIFGVD : 108
AND5   : MKKKIVLFGSLFVLLSCSTVSSEKLGIQKKKKNLEQDATHALMKKLESLYKLSATDNSEIFNKEIESLKKQIDQLHQHGGENEGESLGHLLESEAANESTKKTIFGVD : 108
CAR1   : MKKKIVLFGSLFVLLSCSTVSSEKLGIQKKKKNLEQDATHALMKKLESLYKLSATDNSEIFNKEIESLKKQIDQLHQHGGENEGESLGHLLESEAANESTKKTIFGVD : 108
CAR2   : MKKKIVLFGSLFVLLSCSTVSSEKLGIQKKKKNLEQDATHALMKKLESLYKLSATDNSEIFNKEIESLKKQIDQLHQHGGENEGESLGHLLESEAANESTKKTIFGVD : 108
ORI3   : MKKKIVLFGSLFVLLSCSTVSSEKLGIQKKKKNLEQDAMHALMKKLESLYKLSATDNSEIFNKEMEALKKQIDQLQQHGGANEEESLGHLLESEAANESTKKTIFGVD : 108
PAC7   : MKKKIVLFGSLFVLLSCSTVSSEKLGIQKKKKNLEQDAMHALMKKLESLYKLSATDNSEIFNKEMEALKKQIDQLQQHGGANEEESLGHLLESEAANESTKKTIFGVD : 108
PAC4   : MKKKIVLFGSLFVLLSCSTVSSEKLGIQKKKKNLEQDAMHALMKKLESLYKLSATDNSEIFNKEMEALKKQIDQLQQHGGANEEESLGHLLESEAANESTKKTIFGVD : 108
PAC5   : MKKKIVLFGSLFVLLSCSTVSSEKLGIQKKKKNLEQDAMHALMKKLESLYKLSATDNSEIFNKEMEALKKQIDQLQQHGGANEEESLGHLLESEAANESTKKTIFGVD : 108
AMA2   : MKKKIVLFGSLFVLLSCSTVSSEKLGIQKKKKNLEQDAMHALMKKLESLYKLSATDNSEIFNKEMEALKKQIDQLQQHGGANEEESLGHLLESEAANESTKKTIFGVD : 108
AND6   : MKKKIVLFGSLFVLLSCSTVSSEKLGIQKKKKNLEQDAMHALMKKLESLYKLSATDNSEIFNKEMEALKKQIDQLQQHGGANEEESLGHLLESEAANESTKKTIFGVD : 108
CAR12  : MKKKIVLFGSLFVLLSCSTVSSEKLGIQKKKKNLEQDAMHALMKKLESLYKLSATDNSEIFNKEMEALKKQIDQLQQHGGANEEESLGHLLESEAANESTKKTIFGVD : 108
VCG-I  : MKKKIVLFGSLFVLLSCSTVSSEKLGIQKKKKNLEQDAMHALMKKLESLYKLSATDNSEIFNKEMEALKKQIDQLQQHGGANEEESLGHLLESEAANESTKKTIFGVD : 108
PAC1   : MKKKIVLFGSLFVLLSCSTVSSEKLGIQKKKKNLEQDAMHALMKKLESLYKLSATDNSEIFNKEMEALKKQIDQLQQHGGANEEESLGHLLESEAANESTKKTIFGVD : 108
ORI1   : MKKKIVLFGSLFVLLSCSTVSSEKLGIQKKKKNLEQDAMHALMKKLESLYKLSATDNSEIFNKEMEALKKQIDQLQQHGGANEEESLGHLLESEAANESTKKTIFGVD : 108
CAR4   : MKKKIVLFGSLFVLLSCSTVSSEKLGIQKKKKNLEQDAMHALMKKLESLYKLSATDNSEIFNKEMEALKKQIDQLQQHGGANEEESLGHLLESEAANESTKKTIFGVD : 108
AND14  : MKKKIVLFGSLFVLLSCSTVSSEKLGIQKKKKNLEQDAMHALMKKLESLYKLSATDNSEIFNKEMEALKKQIDQLHQHGGANEEESLGHLLESEAANESTKKTIFGVD : 108
PAC6   : MKKKIVLFGSLFVLLSCSTVSSEKLGIQKKKKNLEQDAMHALMKKLESLYKLSATDNSEIFNKEMEALKKQIDQLHQHGGANEEESLGHLLESEAANESTKKTIFGVD : 108
AND2   : MKKKIVLFGSLFVLLSCSTVSSEKLGIQKKKKNLEQDAMHALMKKLESLYKLSATDNSEIFNKEMEALKKQIDQLQQHGGANEEESLGHLLESEAANESTKKTIFGVD : 108
AND7   : MKKKIVLFGSLFVLLSCSTVSSEKLGIQKKKKNLEQDAMHALMKKLESLYKLSATDNSEIFNKEMEALKKQIDQLQQHGGANEEESLGHLLESEAANESTKKTIFGVD : 108
AND12  : MKKKIVLFGSLFVLLSCSTVSSEKLGIQKKKKNLEQDAMHALMKKLESLYKLSATDNSEIFNKEMEALKKQIDQLQQHGGANEEESLGHLLESEAANESTKKTIFGVD : 108
CAR13  : MKKKIVLFGSLFVLLSCSTVSSEKLGIQKKKKNLEQDAMHALMKKLESLYKLSATDNSEIFNKEMEALKKQIDQLQQHGGANEEESLGHLLESEAANESTKKTIFGVD : 108
CAR11  : MKKKIVLFGSLFVLLSCSTVSSEKLGIQKKKKNLEQDAMHALMKKLESLYKLSATDNSEIFNKEMEALKKQIDQLQQHGGANEEESLGHLLESEAANESTKKTIFGVD : 108
AND13  : MKKKIVLFGSLFVLLSCSTVSSEKLGIQKKKKNLEQDAMHALMKKLESLYKLSATDNSEIFNKEMEALKKQIDQLQQHGGANEEESLGHLLESEAANESTKKTIFGVD : 108
AND10  : MKKKIVLFGSLFVLLSCSTVSSEKLGIQKKKKNLEQDAMHALMKKLESLYKLSATDNSEIFNKEMEALKKQIDQLQQHGGANEEESLGHLLESEAANESTKKTIFGVD : 108
CAR8   : MKKKIVLFGSLFVLLSCSTVSSEKLGIQKKKKNLEQDAMHALMKKLESLYKLSATDNSEIFNKEMEALKKQIDQLQQHGGANEEESLGHLLESEAANESTKKTIFGVD : 108
AND3   : MKKKIVLFGSLFVLLSCSTVSSEKLGIQKKKKNLEQDAMHALMKKLESLYKLSATDNSEIFNKEMEALKKQIDQLQQHGGANEEESLGHLLESEAANESTKKTIFGVD : 108
AMA3   : MKKKIVLFGSLFVLLSCSTVSSEKLGIQKKKKNLEQDAMHALMKKLESLYKLSATDNSEIFNKEMEALKKQIDQLQQHGGANEEESLGHLLESEAANESTKKTIFGVD : 108
AND8   : MKKKIVLFGSLFVLLSCSTVSSEKLGIQKKKKNLEQDAMHALMKKLESLYKLSATDNSEIFNKEMEALKKQIDQLQQHGGANEEESLGHLLESEAANESTKKTIFGVD : 108
AND9   : MKKKIVLFGSLFVLLSCSTVSSEKLGIQKKKKNLEQDAMHALMKKLESLYKLSATDNSEIFNKEMEALKKQIDQLQQHGGANEEESLGHLLESEAANESTKKTIFGVD : 108
CAR3   : MKKKIVLFGSLFVLLSCSTVSSEKLGIQKKKKNLEQDAMHALMKKLESLYKLSATDNSEIFNKEMEALKKQIDQLQQHGGANEEESLGHLLESEAANESTKKTIFGVD : 108
CAR6   : MKKKIVLFGSLFVLLSCSTVSSEKLGIQKKKKNLEQDAMHALMKKLESLYKLSATDNSEIFNKEMEALKKQIDQLQQHGGANEEESLGHLLESEAANESTKKTIFGVD : 108
AND11  : MKKKIVLFGSLFVLLSCSTVSSEKLGIQKKKKNLEQDAMHALMKKLESLYKLTATDNSEIFNKEMEALKKQIDQLQQHGGANEEESLGHLLESEAANESTKKTIFGVD : 108
         mkkkivlfgslfvllscstvsseKLGIQKKKKNLEQDAmHALMKKLESLYKL3ATDNsEIFNKE6eaLKKQIDqLqQHGGaNEeESLGHLLEsEaA1eStKKTIFGVD
```

```
              *       120         *       140         *       160         *       180         *       200         *
Sal-I      : EDDLDNYDGDFTGQSKGKFKGHSFKAQKKVEGNDENI-----GGVPVTGNSASNSQSTGGSGSQNASPPQGSPSDSAQGSQVTNST-----------GSTVTLN : 196
PAC2       : EDDLDNYDADFTGQSKGKIKGHSFKAQKKVEGNDENI-----GGVTVTGNSASNSQSTGGSGSQNASQPEGNPGGNPQGTQVTNSA-----------GSTVTLS : 196
AMA4       : EDDLDNYDGDFTGQSKGKFKGHSFKAQKKVEGNDENI-----GGVPVTGNSASNSQSTGGSGSQNASPTQGSPSDSAQGSQVTNSA-----------GSTVTLS : 196
AND4       : EDDLDNYDGDFTGQSKGKFKGHSFKAQKKVEGNDENI-----GGVPVTGNSASNSQSTGGSGSQNASPTQGSPSDSAQGSQVTNSA-----------GSTVTLS : 196
AND1       : EDDLDNYDGDFTGQSKGKFKGHSFKAQKKVEGNDENI-----GGVPVTGNSASNSQSTGGSGSQNASPPQGSPSDSAQGSQVTNSA-----------GSTVTLS : 196
PAC3       : EDDLDNYDGDFTGQSKGKFKGHSFKAQKKVEGNDENI-----GGVPVTGNSASNSQSTGGSGSQSDSSPQGSPGGNPQGTQVTNSA-----------GSTVTLS : 196
CAR5       : EDDLDNYDGDFTGQSKGKFKGHSFKAQKKVEGNDENFAEAFGGGVPVTGNSASNSQSTGGSGSQSDSSPQGSPGGNPQGTQVTNSA-----------GSTVTLS : 201
AND5       : EDDLDNYDGDFTGQSKGKFKGHSFKAQKKVEGNDENI-----GGVPVTGNSASNSQSTGGSGSQNASPPQGSPSDSAQGSQVTNST-----------GSTVTLN : 196
CAR1       : EDDLDNYDGDFTGQSKGKFKGHSFKAQKKVEGNDENI-----GGVPVTGNSASNSQSTGGSGSQNASPPQGSPSDSAQGSQVTNST-----------GSTVTLN : 196
CAR2       : EDDLDNYDGDFTGQSKGKFKGHSFKAQKKVEGNDENI-----GGVPVTGNSASNSQSTGGSGSQNASPPQGSPSDSAQGSQVTNST-----------GSTVTLN : 196
ORI3       : EDDLDNYDGDFTGQSKGKFKGHSFKAQKKVEGNDENI-----GGVTVTGNSAT-SPPSGGGGSKSPSQPQDNPSDSAQGSP--------GTGGTGGTGGTETLS : 198
PAC7       : EDDLDNYDGDFTGQSKGKFKGHSFKAQKKVEGNDENI-----GGVTVTGNSAT-SPPSGGGGSESPSQPQDNPSDSAQGSP--------GTGGTGGTGGTETLS : 198
PAC4       : EDDLDNYDGDFTGQSKGKFKGHSFKAQKKVEGNDENI-----GGVTVTGNSAT-SPPSGGGGSESPSQPQDNPSDSAQGSP-----GTGGTGGTGGTGGTETLS : 201
PAC5       : EDDLDNYDGDFTGQSKGKFKGHSFKAQKKVEGNDENI-----GGVTVTGNSAT-SPPSGGGGSKSPSQPQDNPSDSAQGSP-----GTGGTGGTGGTGGTETLS : 201
AMA2       : EDDLDNYDGDFTGQSKGKFKGHSFKAQKKVEGNDENI-----GGVTVTGNSAT-SPPSGGGGSESPSQPQDNPGGGAQGSP---ST-----GGTGGTGGTVTLS : 198
AND6       : EDDLDNYDGDFTGQSKGKFKGHSFKAQKKVEGNDENI-----GGVTVTGNSAT-SPPSGGGGSESPSQPQDNPGGGAQGSP---ST-----GGTGGTGGTVTLS : 198
CAR12      : EDDLDNYDGDFTGQSKGKFKGHSFKAQKKVEGNDENI-----GGVTVTGNSAT-SPPSGGGGSESPSQPQDNPGGGAQGSP---ST-----GGTGGTGGTVTLS : 198
VCG-I      : EDDLDNYDGDFTGQSKGKIKGHSFKAQKKVEGNDENI-----GGVTVTGNSAS-SQSTGDLNSQNPSQPEGNNGGGAQGSP--------GTGGTGGTGGTVTLS : 198
PAC1       : EDDLDNYDGDFTGQSKGKIKGHSFKAQKKVEGNDENI-----GGVTVTGNSAS-SQSTGDLNSQNPSQPEGNNGGGAQGSP--------GTGGTGGTGGTVTLS : 198
ORI1       : EDDLDNYDGDFTGQSKGKFKGHSFKAQKKVEGNDENI-----GGVTVTGNSAS-SQSTGDLNSQNPSQPEGNNGGGAQGSP-----------GTGGTGGTVTLS : 195
CAR4       : EDDLDNYDGDFTGQSKGKFKGHSFKAQKKVEGNDENI-----GGVTVTGNSAS-SQSTGDLNSQNPSQPEGNNGGGAQGSP--------GTGGTGGTGGTVTLS : 198
AND14      : EDDLDNYDGDFTGQSKGKFKGHSFKAQKKVEGNDENI-----GGVPVTGNSAS-SQSTGDLNSQNPSPPEGNNGGGAQGSP-----------GTGGTGSTVTLS : 195
PAC6       : EDDLDNYDGDFTGQSKGKFKGHSFKAQKKVEGNDENI-----GGVPVTGNSAS-SQSTGDLNSQNPSQPEGNNGGGAQGSP--------GTGGTGGTGGTVTLS : 198
AND2       : EDDLDNYDGDFTGQSKGKFKGHSFKAQKKVEGNDENI-----GGVTVTGNSAS-SQSTGDLNSQNPSQPEGNNGGGAQGSP--------GTGGTGGTGGTVTLS : 198
AND7       : EDDLDNYDGDFTGQSKGKFKGHSFKAQKKVEGNDENI-----GGVTVTGNSAS-SQSTGDLNSQNPSQPEGNNGGGAQGSP--------GTGGTGGTGGTVTLS : 198
AND12      : EDDLDNYDGDFTGQSKGKFKGHSFKAQKKVEGNDENI-----GGVTVTGNSAS-SQSTGDLNSQNPSQPEGNNGGGAQGSP--------GTGGTGGTGGTVTLS : 198
CAR13      : EDDLDNYDGDFTGQSKGKFKGHSFKAQKKVEGNDENI-----GGVTVTGNSAS-SQSTGDLNSQNPSQPEGNNGGGAQGSP--------GTGGTGGTGGTVTLS : 198
CAR11      : EDDLDNYDGDFTGQSKGKFKGHSFKAQKKVEGNDENI-----GGVTVTGNSAS-SQSTGDLNSQNPSQPEGNNGGGAQGSP--------GTGGTGGTGGTVTLS : 198
AND13      : EDDLDNYDGDFTGQSKGKFKGHSFKAQKKVEGNDENI-----GGVTVTGNSAS-SQSTGDLNSQNPSQPEGNNGGGAQGSP--------GTGGTGGTGGTVTLS : 198
AND10      : EDDLDNYDGDFTGQSKGKFKGHSFKAQKKVEGNDENI-----GGVTVTGNSAS-SQSTGDLNSQNPSQPEGNNGGGAQGSP--------GTGGTGGTGGTVTLS : 198
CAR8       : EDDLDNYDGDFTGQSKGKFKGHSFKAQKKVEGNDENI-----GGVTVTGNSASNSQSTGGSGSQNASPPQDNPGGGAQGSP--------GTGGTGGTGGTVTLS : 199
AND3       : EDDLDNYDGDFTGQSKGKFKGHSFKAQKKVEGNDENI-----GGVPVTGNSASNSQSTGGSGSQNASPPEGNNGGSEQGPQV--------------TEGTVTLN : 193
AMA3       : EDDLDNYDGDFTGQSKGKFKGHSFKAQKKVEGNDENI-----GGVPVTGNSASNSQSTGGSGSQNASPTQGSPGGNPQGTQVTNSA-----------GSTGTLS : 196
AND8       : EDDLDNYDGDFTGQSKGKFKGHSFKAQKKVEGNDENI-----GGVPVTGNSASNSQSTGGSGSQNASPTQGSPGGNPQGTQVTNSA-----------GSTGTLS : 196
AND9       : EDDLDNYDGDFTGQSKGKFKGHSFKAQKKVEGNDENFAEAFGGGAPVTGNSAS-SQPAGVSGSQSDSPPQGSPGGNPQGTQVTNSA-----------GSTGTLS : 200
CAR3       : EDDLDNYDGDFTGQSKGKFKGHSFKAQKKVEGNDENFAEAFGGGAPVTGNSAS-SQPAGVSGSQSDSPPQGSPGGNPQGTQVTNSA-----------GSTGTLS : 200
CAR6       : EDDLDNYDGDFTGQSKGKFKGHSFKAQKKVEGNDENFAEAFGGGAPVTGNSAS-SQPAGVSGSQSDSPPQGSPGGNPQGTQVTNSA-----------GSTGTLS : 200
AND11      : EDDLDNYDGDFTGQSKGKFKGHSFKAQKKVEGNDENF-----GGAPVTGNSAS-SQPAGVWGSQSDSPPQGSPGGDAQGTQVTGSA-----------GSTGTLS : 195
             EDDLDNYDgDFtGQSKGKfKGHSFKAQKKVEGNDENi     GGv VTGNSA3 Sq  G   S   S p2       QG   T      gtggtggtG T TLs
```

```
                        220         *        240         *        260         *        280         *        300         *        320
Sal-I   : APSSSHSTGQPQQSAGVSLPTGTAETVASNTAQTSPPAGSPGGQAATSGQPESGRVPNVKYLDKLYDEVLKTTDAKDEIHVPPFHSKYNDFRKKYEFTMNEREYQIVK : 304
PAC2    : TSSSSQSTGQSQQSNGAEPPAGTTQEVNTNAGQPPPPAGST-GPAATPGQPESGRVPNVKYLDKLYDEVLKTTDAKDEIHVPPFHSKYNDFRKKYEFTMNEREYQIVK : 303
AMA4    : TSSSSQSTGQSQQSNGAEPPAGTTQEVNPNAGQPPPPAGST-GPAATPGQPESGRVPNVKYLDKLYDEVLKTTDAKDEIHVPPFHSKYNDFRKKYEFTMNEREYQIVK : 303
AND4    : TSSSSQSTGQSQQSNGAEPPAGTTQEVNPNAGQPPPPAGST-GPAATPGQPESGRVPNVKYLDKLYDEVLKTTDAKDEIHVPPFHSKYNDFRKKYEFTMNEREYQIVK : 303
AND1    : TSSSSQSTGQSQQSNGAEPPAGTTQEVNPNAGQPPPPAGST-GPAATPGQPESGRVPNVKYLDKLYDEVLKTTDAKDEIHVPPFHSKYNDFRKKYEFTMNEREYQIVK : 303
PAC3    : TSSSSQSTGQSQQSNGAEPPAGTTQEVNPNTAQTSPPAGSPGGQAATSGQPESGRVPNVKYLDKLYDEVLKTTDAKDEIHVPPFHSKYNDFRKKYEFTMNEREYQIVK : 304
CAR5    : TSSSSQSTGQSQQSNGAEPPAGTTQEVNPNAGQTSPPAGSPGGQAATSGQPESGRVPNVKYLDKLYDEVLKTTDAKDEIHVPPFHSKYNDFRKKYEFTMNEREYQIVK : 309
AND5    : APSSSHSTGQPQQSAGVSLPTGTAETVASNTAQTSPPAGSPGGQAATSGQPESGRVPNVKYLDKLYDEVLKTTDAKDEIHVPPFHSKYNDFRKKYEFTMNEREYQIVK : 304
CAR1    : APSSSHSTGQPQQSAGVSLPTGTAETVASNTAQTSPPAGSPGGQAATSGQPESGRVPNVKYLDKLYDEVLKTTDAKDEIHVPPFHSKYNDFRKKYEFTMNEREYQIVK : 304
CAR2    : APSSSHSTGQPQQSAGVSLPTGTAETVASNTAQTSPPAGSPGGQAATSGQPESGRVPNVKYLDKLYDEVLKTTDAKDEIHVPPFHSKYNDFRKKYEFTMNEREYQIVK : 304
ORI3    : TSSSSHSTGQPQQSAGVSLPTGTAETVASNTAQTSPPAGSPGGQAATSGQPESGRVPNVKYLDKLYDEVLKTTDAKDEIHVPPFHSKYNDFRKKYEFTMNEREYQIVK : 306
PAC7    : TSSSSHSTGQPQQSAGVSLPTGTAETVASNTAQTSPPAGSPGGQAATSGQPESGRVPNVKYLDKLYDEVLKTTDAKDEIHVPPFHSKYNDFRKKYEFTMNEREYQIVK : 306
PAC4    : TSSSSHSTGQPQQSAGVSLPTGTAETVASNTAQTSPPAGSPGGQAATSGQPESGRVPNVKYLDKLYDEVLKTTDAKDEIHVPPFHSKYNDFRKKYEFTMNEREYQIVK : 309
PAC5    : TSSSSHSTGQPQQSAGVSLPTGTAETVASNTAQTSPPAGSPGGQAATSGQPESGRVPNVKYLDKLYDEVLKTTDAKDEIHVPPFHSKYNDFRKKYEFTMNEREYQIVK : 309
AMA2    : TSSSGPSTGGSQPSAGVAPSVGNAEAVVTNTEHSSPPAGLPGGQAAPPGQPESGRVPNVKYLDKLYDEVLKTTDAKDEIHVPPFHSKYNDFRKKYEFTMNEREYQIVK : 306
AND6    : TSSSGPSTGGSQPSAGVAPSVGNAEAVVTNTEHSSPPAGLPGGQAAPPGQPESGRVPNVKYLDKLYDEVLKTTDAKDEIHVPPFHSKYNDFRKKYEFTMNEREYQIVK : 306
CAR12   : TSSSGPSTGGSQPSAGVAPSVGNAEAVVTNTEHSSPPAGLPGGQAAPPGQPESGRVPNVKYLDKLYDEVLKTTDAKDEIHVPPFHSKYNDFRKKYEFTMNEREYQIVK : 306
VCG-I   : TSSSSPSTGGSQPSAGVSLPTGTAETVASNTAQTSPPAGLPGGQAATPGQPESGRVPNVKYLDKLYDEVLKTTDAKDEIHVPPFHSKYNDFRKKYEFTMNEREYQIVK : 306
PAC1    : TSSSSPSTGGSQPSAGVSLPTGTAETVASNTAQTSPPAGLPGGQAATPGQPESGRVPNVKYLDKLYDEVLKTTDAKDEIHVPPFHSKYNDFRKKYEFTMNEREYQIVK : 306
ORI1    : TSSSSHSTGQPQQSAGVSLPTGTAETVASNTAQTSPPAGSPGGQAATSGQPESGRVPNVKYLDKLYDEVLKTTDAKDEIHVPPFHSKYNDFRKKYEFTMNEREYQIVK : 303
CAR4    : TSSSSHSTGQPQQSAGVSLPTGTAETVASNTAQTSPPAGSPGGQAATSGQPESGRVPNVKYLDKLYDEVLKTTDAKDEIHVPPFHSKYNDFRKKYEFTMNEREYQIVK : 306
AND14   : TSSSSHSTGQPQQSAGVSLPTGTAETVASNTAQTSPPAGSPGGQAATSGQPESGRVPNVKYLDKLYDEVLKTTDAKDEIHVPPFHSKYNDFRKKYEFTMNEREYQIVK : 303
PAC6    : TSSSSHSTGQPQQSAGVSLPTGTAETVASNTAQTSPPAGSPGGQAATSGQPESGRVPNVKYLDKLYDEVLKTTDAKDEIHVPPFHSKYNDFRKKYEFTMNEREYQIVK : 306
AND2    : TSSSSHSTGQPQQSAGVSLPTGTAETVASNTAQTSPPAGSPGGQAATSGQPESGRVPNVKYLDKLYDEVLKTTDAKDEIHVPPFHSKYNDFRKKYEFTMNEREYQIVK : 306
AND7    : TSSSSHSTGQPQQSAGVSLPTGTAETVASNTAQTSPPAGSPGGQAATSGQPESGRVPNVKYLDKLYDEVLKTTDAKDEIHVPPFHSKYNDFRKKYEFTMNEREYQIVK : 306
AND12   : TSSSSHSTGQPQQSAGVSLPTGTAETVASNTAQTSPPAGSPGGQAATSGQPESGRVPNVKYLDKLYDEVLKTTDAKDEIHVPPFHSKYNDFRKKYEFTMNEREYQIVK : 306
CAR13   : TSSSSHSTGQPQQSAGVSLPTGTAETVASNTAQTSPPAGSPGGQAATSGQPESGRVPNVKYLDKLYDEVLKTTDAKDEIHVPPFHSKYNDFRKKYEFTMNEREYQIVK : 306
CAR11   : TSSSSHSTGQPQQSAGVSLPTGTAETVASNTAQTSPPAGSPGGQAATSGQPESGRVPNVKYLDKLYDEVLKTTDAKDEIHVPPFHSKYNDFRKKYEFTMNEREYQIVK : 306
AND13   : TSSSSHSTGQPQQSAGVSLPTGTAETVASNTAQTSPPAGSPGGQAATSGQPESGRVPNVKYLDKLYDEVLKTTDAKDEIHVPPFHSKYNDFRKKYEFTMNEREYQIVK : 306
AND10   : TSSSSHSTGQPQQSAGVSLPTGTAETVASNTAQTSPPAGSPGGQAATSGQPESGRVPNVKYLDKLYDEVLKTTDAKDEIHVPPFHSKYNDFRKKYEFTMNEREYQIVK : 306
CAR8    : TSSSSHSTGQPQQSAGVSLPTGTAETVASNTAQTSPPAGSPGGQAATSGQPESGRVPNVKYLDKLYDEVLKTTDAKDEIHVPPFHSKYNDFRKKYEFTMNEREYQIVK : 307
AND3    : TPSSGPSTGGSQPSAGVSLPTGTAETVASNTAQTSPPAGSPGGQAATSGQPESGRVPNVKYLDKLYDEVLKTTDAKDEIHVPPFHSKYNDFRKKYEFTMNEREYQIVK : 301
AMA3    : TSSSSQSTGQSQPSAGVAPSVGNAEAVVTNTEHSSPPAGLPGGQAATSGQPESGRVPNVKYLDKLYDEVLKTTDAKDEIHVPPFHSKYNDFRKKYEFTMNEREYQIVK : 304
AND8    : TSSSSQSTGQSQPSAGVAPSVGNAEAVVTNTEHSSPPAGLPGGQAATSGQPESGRVPNVKYLDKLYDEVLKTTDAKDEIHVPPFHSKYNDFRKKYEFTMNEREYQIVK : 304
AND9    : TSSSSQSTGGSQPSAGVALPTGTAETVASNTAQTSPPAGLPGGQAATPGQPESGRVPNVKYLDKLYDEVLKTTDAKDEIHVPPFHSKYNDFRKKYEFTMNEREYQIVK : 308
CAR3    : TSSSSQSTGGSQPSAGVALPTGTAETVASNTAQTSPPAGLPGGQAATPGQPESGRVPNVKYLDKLYDEVLKTTDAKDEIHVPPFHSKYNDFRKKYEFTMNEREYQIVK : 308
CAR6    : TSSSSQSTGGSQPSAGVALPTGTAETVASNTAQTSPPAGLPGGQAATPGQPESGRVPNVKYLDKLYDEVLKTTDAKDEIHVPPFHSKYNDFRKKYEFTMNEREYQIVK : 308
AND11   : TSSSSQSTGGSQPSAGVAPSVGNAEAVVTNTEHSSPPAGLPGGQAATPGQPESGRVPNVKYLDKLYDEVLKTTDAKDEIHVPPFHSKYNDFRKKYEFTMNEREYQIVK : 303
          tsSss STGq Q S g   p Gt 2 V  n  q PPAG   G AAt GQPESGRVPNVkYLDKLYDEVLKTTDak1eIHVPPFHSKYNDFRKkYEFTMNEREYQIVK
```

```
                     *       340         *       360         *       380         *       400
Sal-I     : NLFDAFFKKDGNPSPADAVSFFKKMLNDPNVQKEFDNFVHGLYGFAKRHNYLRGERMTDTKLYDELLKNVVNLLNTIEVK : 384
PAC2      : NLFDAFFKKDGNPSPADAVSFFKKMLNDPNVQKEFDNFVHGLYGFAKRHNYLRGERMTDTNLYDKLLKNVVNLLNTIEVK : 383
AMA4      : NLFDAFFKKDGNPSPADAVSFFKKMLNDPNVQKEFDNFVHGLYGFAKRHNYLRGERMTDTKLYDKLLKNVVNLLNTIEVK : 383
AND4      : NLFDAFFKKDGNPSPADAVSFFKKMLNDPNVQKEFDNFVHGLYGFAKRHNYLRGERMTDTKLYDELLKNVVNLLNTIEVK : 383
AND1      : NLFDAFFKKDGNPSPADAVSFFKKMLNDPNVQKEFDNFVHGLYGFAKRHNYLRGERMTDTKLYDKLLKNVVNLLNTIEVK : 383
PAC3      : NLFDAFFKKDGNPSPADAVSFFKKMLNDPNVQKEFDNFVHGLYGFAKRHNYLRGERMTDTKLYDELLKNVVNLLNTIEVK : 384
CAR5      : NLFDAFFKKDGNPSPADAVSFFKKMLNDPNVQKEFDNFVHGLYGFAKRHNYLRGERMTDTKLYDELLKNVVNLLNTIEVK : 389
AND5      : NLFDAFFKKDGNPSPADAVSFFKKMLNDPNVQKEFDNFVHGLYGFAKRHNYLRGERMTDTKLYDELLKNVVNLLNTIEVK : 384
CAR1      : NLFDAFFKKDGNPSPADAVSFFKKMLNDPNVQKEFDNFVHGLYGFAKRHNYLRGERMTDTKLYDELLKNVVNLLNTIEVK : 384
CAR2      : NLFDAFFKKDGNPSPADAVSFFKKMLNDPNVQKEFDNFVHGLYGFAKRHNYLRGERMTDTKLYDELLKNVVNLLNTIEVK : 384
ORI3      : NLFDAFFKKDGNPSPADAVSFFKKMLNDPNVQKEFDNFVHGLYGFAKRHNYLRGERMTDTQLYDELLKNVVNLLNTIEVK : 386
PAC7      : NLFDAFFKKDGNPSPADAVSFFKKMLNDPNVQKEFDNFVHGLYGFAKRHNYLRGERMTDTQLYDELLKNVVNLLNTIEVK : 386
PAC4      : NLFDAFFKKDGNPSPADAVSFFKKMLNDPNVQKEFDNFVHGLYGFAKRHNYLRGERMTDTQLYDELLKNVVNLLNTIEVK : 389
PAC5      : NLFDAFFKKDGNPSPADAVSFFKKMLNDPNVQKEFDNFVHGLYGFAKRHNYLRGERMTDTQLYDELLKNVVNLLNTIEVK : 389
AMA2      : NLFDAFFKKDGNPSPADAVSFFKKMLNDPNVQKEFDNFVHGLYGFAKRHNYLRGERMTDTNLYDELLKNVVNLLNTIEVK : 386
AND6      : NLFDAFFKKDGNPSPADAVSFFKKMLNDPNVQKEFDNFVHGLYGFAKRHNYLRGERMTDTNLYDELLKNVVNLLNTIEVK : 386
CAR12     : NLFDAFFKKDGNPSPADAVSFFKKMLNDPNVQKEFDNFVHGLYGFAKRHNYLRGERMTDTKLYDKLLKNVVNLLNTIEVK : 386
VCG-I     : NLFGAFFKKDGNPSPADAVSFFKKMLNDPNVQKEFDNFVHGLYGFAKRHNYLRGERMTDTQLYDELLKNVVNLLNTIEVK : 386
PAC1      : NLFGAFFKKDGNPSPADAVSFFKKMLNDPNVQKEFDNFVHGLYGFAKRHNYLRGERMTDTQLYDELLKNVVNLLNTIEVK : 386
ORI1      : NLFDAFFKKDGNPSPADAVSFFKKMLNDPNVQKEFDNFVHGLYGFAKRHNYLRGERMTDTKLYDELLKNVVNLLNTIEVK : 383
CAR4      : NLFDAFFKKDGNPSPADAVSFFKKMLNDPNVQKEFDNFVHGLYGFAKRHNYLRGERMTDTQLYDELLKNVVNLLNTIEVK : 386
AND14     : NLFDAFFKKDGNPSPADAVSFFKKMLNDPNVQKEFDNFVHGLYGFAKRHNYLRGERMTDTKLYDELLKNVVNLLNTIEVK : 383
PAC6      : NLFDAFFKKDGNPSPADAVSFFKKMLNDPNVQKEFDNFVHGLYGFAKRHNYLRGERMTDTKLYDELLKNVVNLLNTIEVK : 386
AND2      : NLFDAFFKKDGNPSPADAVSFFKKMLNDPNVQKEFDNFVHGLYGFAKRHNYLRGERMTDTKLYDELLKNVVNLLNTIEVK : 386
AND7      : NLFDAFFKKDGNPSPADAVSFFKKMLNDPNVQKEFDNFVHGLYGFAKRHNYLRGERMTDTKLYDELLKNVVNLLNTIEVK : 386
AND12     : NLFDAFFKKDGNPSPADAVSFFKKMLNDPNVQKEFDNFVHGLYGFAKRHNYLRGERMTDTKLYDELLKNVVNLLNTIEVK : 386
CAR13     : NLFDAFFKKDGNPSPADAVSFFKKMLNDPNVQKEFDNFVHGLYGFAKRHNYLRGERMTDTKLYDELLKNVVNLLNTIEVK : 386
CAR11     : NLFDAFFKKDGNPSPADAVSFFKKMLNDPNVQKEFDNFVHGLYGFAKRHNYLRGERMTDTKLYDELLKNVVNLLNTIEVK : 386
AND13     : NLFDAFFKKDGNPSPADAVSFFKKMLNDPNVQKEFDNFVHGLYGFAKRHNYLRGERMTDTKLYDELLKNVVNLLNTIEVK : 386
AND10     : NLFDAFFKKDGNPSPADAVSFFKKMLNDPNVQKEFDNFVHGLYGFAKRHNYLRGERMTDTKLYDELLKNVVNLLNTIEVK : 386
CAR8      : NLFDAFFKKDGNPSPADAVSFFKKMLNDPNVQKEFDNFVHGLYGFAKRHNYLRGERMTDTKLYDKLLKNVVNLLNTIEVK : 387
AND3      : NLFDAFFKKDGNPSPADAVSFFKKMLNDPNVQKEFDNFVHGLYGFAKRHNYLRGERMTDTKLYDELLKNVVNLLNTIEVK : 381
AMA3      : NLFDAFFKKDGNPSPADAVSFFKKMLNDPNVQKEFDNFVHGLYGFAKRHNYLRGERMTDTKLYDELLKNVVNLLNTIEVK : 384
AND8      : NLFDAFFKKDGNPSPADAVSFFKKMLNDPNVQKEFDNFVHGLYGFAKRHNYLRGERMTDTKLYDELLKNVVNLLNTIEVK : 384
AND9      : NLFDAFFKKDGNPSPADAVSFFKKMLNDPNVQKEFDNFVHGLYGFAKRHNYLRGERMTDTKLYDELLKNVVNLLNTIEVK : 388
CAR3      : NLFDAFFKKDGNPSPADAVSFFKKMLNDPNVQKEFDNFVHGLYGFAKRHNYLRGERMTDTKLYDELLKNVVNLLNTIEVK : 388
CAR6      : NLFDAFFKKDGNPSPADAVSFFKKMLNDPNVQKEFDNFVHGLYGFAKRHNYLRGERMTDTKLYDELLKNVVNLLNTIEVK : 388
AND11     : NLFDAFFKKDGNPSPADAVSFFKKMLNDPNVQKEFDNFVHGLYGFAKRHNYLRGERMTDTKLYDELLKNVVNLLNTIEVK : 383
            nLFdAfFkKDGNpsPADAVsFFKKMLNDPNVQKEFDNFVHGLYGFAKRHNYLRGERMTDT LYDeLLKNVVNLLNTIEVK
```
