# Supplementary material for: Genetic Diversity and Selection in Three Plasmodium vivax Merozoite Surface Protein 7 (Pvmsp-7) Genes in a Colombian Population
Source: PLoS One. 2012 Sep 25;7(9):e45962. doi: 10.1371/journal.pone.0045962 (PMC3458108; doi:10.1371/journal.pone.0045962)
Supplement: Figure S4 — The linkage disequilibrium (LD) plot for P. vivax Pvmsp-7C (A), Pvmsp-7H (B) and Pvmsp-7I (C). Trace line represents the regression line which declined as nucleotide distance increased suggesting that intragenic recombination was taking place in msp-7 genes. (PDF) [file pone.0045962.s004.pdf]

```
                 *        20         *        40         *        60         *        80         *        100        *
Sal-I  : MRGKYVMMSSFLFFLSLHTVPCEKLGIQKKKKNLEQDAMHALMKKLESLYKLSATDNGEIFNKEIDALKKQIDQLQQHGGDNEGESLGHLLENEAADDSGKKTIFGVDED : 110
AND11  : ------MMSSFLFFLSLHTVPCEKLGIQKKKKNLEQDAMHALMKKLESLYKLSATDNGEIFNKEIDALKKQIDQLQQHGGDNEGESLGHLLENEAADDSGKKTIFGVDED : 104
CAR12  : ------MMSSFLFFLSLHTVPCEKLGIQKKKKNLEQDAMHALMKKLESLYKLSATDNGEIFNKEIDALKKQIDQLQQHGGDNEGESLGHLLENEAADDSGKKTIFGVDED : 104
AND6   : ------MMSSFLFFLSLHTVPCEKLGIQKKKKNLEQDAMHALMKKLESLYKLSATDNGEIFNKEIDALKKQIDQLQQHGGDNEGESLGHLLENEAADDSGKKTIFGVDED : 104
AMA5   : ------MMSSFLFFLSLHTVPCEKLGIQKKKKNLEQDAMHALMKKLESLYKLSATDNGEIFNKEIDALKKQIDQLQQHGGDNEGESLGHLLENEAADDSGKKTIFGVDED : 104
AND1   : ------MMSSFLFFLSLHTVPCEKLGIQKKKKNLEQDAMHALMKKLESLYKLSATDNGEIFNKEIDALKKQIDQLQQHGGDNEGESLGHLLENEAADDSGKKTIFGVDED : 104
AND5   : ------MMSSFLFFLSLHTVPCEKLGIQKKKKNLEQDAMHALMKKLESLYKLSATDNGEIFNKEIDALKKQIDQLQQHGGDNEGESLGHLLENEAADDSGKKTIFGVDED : 104
CAR11  : ------MMSSFLFFLSLHTVPCEKLGIQKKKKNLEQDAMHALMKKLESLYKLSATDNGEIFNKEIDALKKQIDQLQQHGGDNEGESLGHLLENEAADDSGKKTIFGVDED : 104
AND12  : ------MMSSFLFFLSLHTVPCEKLGIQKKKKNLEQDAMHALMKKLESLYKLSATDNGEIFNKEIDALKKQIDQLQQHGGDNEGESLGHLLENEAADDSGKKTIFGVDED : 104
ORI3   : ------MMSSFLFFLSLHTVPCEKLGIQKKKKNLEQDAMHALMKKLESLYKLSATDNGEIFNKEIDALKKQIDQLQQHGGDNEGESLGHLLENEAADDSGKKTIFGVDED : 104
AND9   : ------MMSSFLFFLSLHTVPCEKLGIQKKKKNLEQDAMHALMKKLESLYKLSATDNGEIFNKEIDALKKQIDQLQQHGGDNEGESLGHLLENEAADDSGKKTIFGVDED : 104
PAC6   : ------MMSSFLFFLSLHTVPCEKLGIQKKKKNLEQDAMHALMKKLESLYKLSATDNGEIFNKEIDALKKQIDQLQQHGGDNEGESLGHLLENEAADDSGKKTIFGVDED : 104
VCG-I  : ------MMSSFLFFLSLHTVPCEKLGIQKKKKNLEQDAMHALMKKLESLYKLSATDNGEIFNKEIDALKKQIDQLQQHGGDNEGESLGHLLENEAADDSGKKTIFGVDED : 104
PAC1   : ------MMSSFLFFLSLHTVPCEKLGIQKKKKNLEQDAMHALMKKLESLYKLSATDNGEIFNKEIDALKKQIDQLQQHGGDNEGESLGHLLENEAADDSGKKTIFGVDED : 104
ORI2   : ------MMSSFLFFLSLHTVPCEKLGIQKKKKNLEQDAMHALMKKLESLYKLSATDNGEIFNKEIDALKKQIDQLQQHGGDNEGESLGHLLENEAADDSGKKTIFGVDED : 104
CAR2   : ------MMSSFLFFLSLHTVPCEKLGIQKKKKNLEQDAMHALMKKLESLYKLSATDNGEIFNKEIDALKKQIDQLQQHGGDNEGESLGHLLENEAADDSGKKTIFGVDED : 104
CAR4   : ------MMSSFLFFLSLHTVPCEKLGIQKKKKNLEQDAMHTLMKKLESLYKLSATDNGEIFNKEIDALKKQIDQLQQHGGDNEGESLGHLLENEAADDSGKKTIFGVDED : 104
ORI1   : ------MMSSFLFFLSLHTVPCEKLGIQKKKKNLEQDAMHTLMKKLESLYKLSATDNGEIFNKEIDALKKQIDQLQQHGGDNEGESLGHLLENEAADDSGKKTIFGVDED : 104
AND7   : ------MMSSFLFFLSLHTVPCEKLGIQKKKKNLEQDAMHTLMKKLESLYKLSATDNGEIFNKEIDALKKQIDQLQQHGGDNEGESLGHLLENEAADDSGKKTIFGVDED : 104
AND2   : ------MMSSFLFFLSLHTVPCEKLGIQKKKKNLEQDAMHALMKKLESLYKLSATDNGEIFNKEIDALKKQIDQLQQHGGDNEGESLGHLLENEAADDSGKKTIFGVDED : 104
CAR5   : ------MMSSFLFFLSLHTVPCEKLGIQKKKKNLEQDAMHALMKKLESLYKLSATDNGEIFNKEIDALKKQIDQLQQHGGDNEGESLGHLLENEAADDSGKKTIFGVDED : 104
CAR6   : ------MMSSFLFFLSLHTVPCEKLGIQKKKKNLEQDAMHTLMKKLESLYKLSATDNGEIFNKEIDALKKQIDQLQQHGGDNEGESLGHLLENEAADDSGKKTIFGVDED : 104
CAR3   : ------MMSSFLFFLSLHTVPCEKLGIQKKKKNLEQDAMHALMKKLESLYKLSATDNGEIFNKEIDALKKQIDQLQQHGGDNEGESLGHLLENEAADDSGKKTIFGVDED : 104
AND13  : ------MMSSFLFFLSLHTVPCEKLGIQKKKKNLEQDAMHALMKKLESLYKLSATDNGEIFNKEIDALKKQIDQLQQHGGDNEGESLGHLLENEAADDSGKKTIFGVDED : 104
AND10  : ------MMSSFLFFLSLHTVPCEKLGIQKKKKNLEQDAMHALMKKLESLYKLSATDNGEIFNKEIDALKKQIDQLQQHGGDNEGESLGHLLENEAADDSGKKTIFGVDED : 104
CAR13  : ------MMSSFLFFLSLHTVPCEKLGIQKKKKNLEQDAMHALMKKLESLYKLSATDNGEIFNKEIDALKKQIDQLQQHGGDNEGESLGHLLENEAADDSGKKTIFGVDED : 104
PAC3   : ------MMSSFLFFLSLHTVPCEKLGIQKKKKNLEQDAMHALMKKLESLYKLSATDNGEIFNKEIDALKKQIDQLQQHGGDNEGESLGHLLENEAADDSGKKTIFGVDED : 104
CAR10  : ------MMSSFLFFLSLHTVPCEKLGIQKKKKNLEQDAMHALMKKLESLYKLSATDNGEIFNKEIDALKKQIDQLQQHGGDNEGESLGHLLENEAADDSGKKTIFGVDED : 104
CAR8   : ------MMSSFLFFLSLHTVPCEKLGIQKKKKNLEQDAMHALMKKLESLYKLSATDNGEIFNKEIDALKKQIDQLQQHGGDNEGESLGHLLENEAADDSGKKTIFGVDED : 104
PAC4   : ------MMSSFLFFLSLHTVPCEKLGIQKKKKNLEQDAMHALMKKLESLYKLSATDNGEIFNKEIDALKKQIDQLQQHGGDNEGESLGHLLENEAADDSGKKTIFGVDED : 104
PAC5   : ------MMSSFLFFLSLHTVPCEKLGIQKKKKNLEQDAMHALMKKLESLYKLSATDNGEIFNKEIDALKKQIDQLQQHGGDNEGESLGHLLENEAADDSGKKTIFGVDED : 104
AMA3   : ------MMSSFLFFLSLHTVPCEKLGIQKKKKNLEQDAMHALMKKLESLYKLSATDNGEIFNKEIDALKKQIDQLQQHGGDNEGESLGHLLENEAADDSGKKTIFGVDED : 104
AND3   : ------MMSSFLFFLSLHTVPCEKLGIQKKKKNLEQDAMHALMKKLESLYKLSATDNGEIFNKEIDALKKQIDQLQQHGGDNEGESLGHLLENEAADDSGKKTIFGVDED : 104
ORI4   : ------MMSSFLFFLSLHTVPCEKLGIQKKKKNLEQDAMHALMKKLESLYKLSATDNGEIFNKEIDALKKQIDQLQQHGGDNEGESLGHLLENEAADDSGKKTIFGVDED : 104
CAR1   : ------MMSSFLFFLSLHTVPCEKLGIQKKKKNLEQDAMHALMKKLESLYKLSATDNGEIFNKEIDALKKQIDQLQQHGGDNEGESLGHLLENEAADDSGKKTIFGVDED : 104
AND8   : ------MMSSFLFFLSLHTVPCEKLGIQKKKKNLEQDAMHALMKKLESLYKLSATDNGEIFNKEIDALKKQIDQLQQHGGDNEGESLGHLLENEAADDSGKKTIFGVDED : 104
ORI5   : ------MMSSFLFFLSLHTVPCEKLGIQKKKKNLEQDAMHALMKKLESLYKLSATDNGEIFNKEIDALKKQIDQLQQHGGDNEGESLGHLLENEAADDSGKKTIFGVDED : 104
PAC7   : ------MMSSFLFFLSLHTVPCEKLGIQKKKKNLEQDAMHALMKKLESLYKLSATDNGEIFNKEIDALKKQIDQLQQHGGDNEGESLGHLLENEAADDSGKKTIFGVDED : 104
PAC2   : ------MMSSFLFFLSLHTVPCEKLGIQKKKKNLEQDAMHALMKKLESLYKLSATDNGEIFNKEIDALKKQIDQLQQHGGDNEGESLGHLLENEAADDSGKKTIFGVDED : 104
PAC8   : ------MMSSFLFFLSLHTVPCEKLGIQKKKKNLEQDAMHALMKKLESLYKLSATDNGEIFNKEIDALKKQIDQLQQHGGDNEGESLGHLLENEAADDSGKKTIFGVDED : 104
CAR7   : ------MMSSFLFFLSLHTVPCEKLGIQKKKKNLEQDAMHALMKKLESLYKLSATDNGEIFNKEIDALKKQIDQLQQHGGDNEGESLGHLLENEAADDSGKKTIFGVDED : 104
AMA4   : ------MMSSFLFFLSLHTVPCEKLGIQKKKKNLEQDAMHALMKKLESLYKLSATDNGEIFNKEIDALKKQIDQLQQHGGDNEGESLGHLLENEAADDSGKKTIFGVDED : 104
AND4   : ------MMSSFLFFLSLHTVPCEKLGIQKKKKNLEQDAMHALMKKLESLYKLSATDNGEIFNKEIDALKKQIDQLQQHGGDNEGESLGHLLENEAADDSGKKTIFGVDED : 104
               mmssflfflslhtvpceKLGIQKKKKNLEQDAMHaLMKKLESLYKLSATDNGEIFNKEIDALKKQIDQLQQHGGDNEGESLGHLLENEAADDSGKKTIFGVDED
```

```
                120         *       140         *       160         *       180         *       200         *       220
Sal-I  : DLDNYDADFIGQGKKIITKGAADKEEEEEDDEDEEAVEGD-------EEAGERD----EAEAEAVRGDTSHQSGSGDLTPSPPGSSERAPAVAPGETPRVTATSQGPSQT : 209
AND11  : DLDNYDADFIGQGKKIITKGAADKEEEEEDDEDEEAVEGD-------EEAGERD----EAEAEAVRGDTSHQSGSGDLTPSPPGSSERAPAVAPGETPRDTATSQGPSQT : 203
CAR12  : DLDNYDADFIGQGKKIITKGAADKEEEEEDDEDEEAVEGD-------EEAGERDEA--EAEAEAVRGDTSHQSGSGDLTPSPPGSSERAPAVAPGETPRVTATSQGPSQT : 205
AND6   : DLDNYDADFIGQGKKIITKGAADKEEEEEDDEDEEAVEGD-------EEAGERDEA--EAEAEAVRGDTSHQSGSGDLTPSPPGSSERAPAVAPGETPRVTATSQGPSQT : 205
AMA5   : DLDNYDADFIGQGKKIITKGAADKEEEEEDDEDEEAVEGD-------EEAGERDEAEAEAEAEAVRGDTSHQSGSGDLTPSPPGSSERAPAVAPGETPRVTATSQGPSQT : 207
AND1   : DLDNYDADFIGQGKKIITKGAADKEEEEEDDEDEEAVEGD-------EEAGERDEA--EAEAEAVRGDTSHQSGSGDLTPSPPGSSERAPAVAPGETPRVTATSQGPSQT : 205
AND5   : DLDNYDADFIGQGKKIITKGAADKEEEEEDDEDEEAVEGD-------EEAGERD----EAEAEAVRGDTSHQSGSGDLTPSPPGSSERAPAVAPGETPRVTATSQGPSQT : 203
CAR11  : DLDNYDADFIGQGKKIITKGAADKEEEEEDDEDEEAVEGD-------EEAGERD----EAEAEAVRGDTSHQSGSGDLTPSPPGSSERAPAVAPGETPRVTATSQGPSQT : 203
AND12  : DLDNYDADFIGQGKKIITKGAADKEEEEEDDEDEEAVEGD-------EEAGERD----EAEAEAVRGDTSHQSGSGDLTPSPPGSSERAPAVAPGETPRVTATSQGPSQT : 203
ORI3   : DLDNYDADFIGQGKKIITKGAADKEEEEEDDEDEEAVEGD-------EEAGERD----EAEAEAVRGDTSHQSGSGDLTPSPPGSSERAPAVAPGETPRDTATSQGPSQT : 203
AND9   : DLDNYDADFIGQGKKIITKGAADKEEEEEDDEDEEAVEGD-------EEAGERD----EAEAEAVRGDTSHQSGSGDLTPSPPGSSERAPAVAPGETPRDTATSQGPSQT : 203
PAC6   : DLDNYDADFIGQGKKIITKGAADKEEEEEDDEDEEAVEGD-------EEAGERD----EAEAEAVRGDTSHQSGSGDLTPSPPGSSERAPAVAPGETPRDTATSQGPSQT : 203
VCG-I  : DLDNYDADFIGQGKKIITKGAADKEEEEEDDEDEEAVEGD-------EEAGERD----EAEAEAVRGDTSHQSGSGDLTPSPPGSSERAPAVAPGETPRDTATSQGPSQT : 203
PAC1   : DLDNYDADFIGQGKKIITKGAADKEEEEEDDEDEEAVEGD-------EEAGERD----EAEAEAVRGDTSHQSGSGDLTPSPPGSSERAPAVAPGETPRDTATSQGPSQT : 203
ORI2   : DLDNYDADFIGQGKKIITKGAADKEEEEEDDEDEEAVEGD-------EEAGERDEA--EAEAEAVRGDTSHQSGSGDLTPSPPGSSERAPAVAPGETPRVTATSQGPSQT : 205
CAR2   : DLDNYDADFIGQGKKIITKGAADKEEEEEDDEDEEAVEGD-------EEAGERD----EAEAEAVRGDTSHQSGSGDLTPSPPGSSERAPAVAPGETPRVTATSQGPSQT : 203
CAR4   : DLDNYDADFIGQGKKIITKGAADKEEEEEDDEDEEAVEGD-------EEAGERD----EAEAEAVRGDTSHQSGSGDLTPSPPGSSERAPAVAPGETPRVTATSQGPSQT : 203
ORI1   : DLDNYDADFIGQGKKIITKGAADKEEEEEDDEDEEAVEGD-------EEAGERD----EAEAEAVRGDTSHQSGSGDLTPSPPGSSERAPAVAPGETPRVTATSQGPSQT : 203
AND7   : DLDNYDADFIGQGKKIITKGAADKEEEEEDDEDEEAVEGD-------EEAGERD----EAEAEAVRGDTSHQSGSGDLTPSPPGSSERAPAVAPGETPRVTATSQGPSQT : 203
AND2   : DLDNYDADFIGQGKKIITKGAADKEEEEEDDEDEEAVEGD-------EEAGERD----EAEAEAVRGDTSHQSGSGDLTPSPPGSSERAPAVAPGETPRVTATSQGPSQT : 203
CAR5   : DLDNYDADFIGQGKKIITKGAADKEEEEEDDEDEEAVEGD-------EEAGERD----EAEAEAVRGDTSHQSGSGDLTPSPPGSSERAPAVAPGETPRVTATSQGPSQT : 203
CAR6   : DLDNYDADFIGQGKKIITKGAADKEEEEEDDEDEEAVEGD-------EEAGERD----EAEAEAVRGDTSHQSGSGDLTPSPPGSSERAPAVAPGETPRVTATSQGPSQT : 203
CAR3   : DLDNYDADFIGQGKKIITKGAADKEEEEEDDEDEEAVEGD-------EEAGERD----EAEAEAVRGDTSHQSGSGDLTPSPPGSSERAPAVAPGETPRDTATSQGPSQT : 203
AND13  : DLDNYDADFIGQGKKIITKGAADKEEEEEDDEDEEAVEGD-------EEAGERD----EAEAEAVRGDTSHQSGSGDLTPSPPGSSERAPAVAPGETPRVTATSQGPSQT : 203
AND10  : DLDNYDADFIGQGKKIITKGAADKEEEEEDDEDEEAVEGD-------EEAGERD----EAEAEAVRGDTSHQSGSGDLTPSPPGSSERAPAVAPGETPRVTATSQGPSQT : 203
CAR13  : DLDNYDADFIGQGKKIITKGAADKEEEEEDDEDEEAVEGD-------EEAGERD----EAEAEAVRGDTSHQSGSGDLTPSPPGSSERAPAVAPGETPRVTATSQGPSQT : 203
PAC3   : DLDNYDADFIGQGKKIITKGAADKEEEEEDDEDEEAVEGD-------EEAGERD----EAEAEAVRGDTSHQSGSGDLTPSPPGSSERAPAVAPGETPRVTATSQGPSQT : 203
CAR10  : DLDNYDADFIGQGKKIITKGAADKEEEEEDDEDEEAVEGDEEAVEGDEEAGEED----EAEAEAVRGDTSHQSGSGDLTPSPPGSSERAPAVAPGETPRVTATSQGPSQT : 210
CAR8   : DLDNYDADFIGQGKKIITKGAVDSNEEDGGDEDEEAVEGD-EA-----EAGEEG------EEEATNEVASHQSGSGDLTPSPPASGELAPATARGETATDSPTSQEPSQT : 202
PAC4   : DLDNYDADFIGQGKKIITKGVVDSNEEDGGDEDEEAVEGD-EA-----EAGEEG------EEEATNEVASHQSDSEASGESDPASGELAPATARGENATDSPTSQEPSQT : 202
PAC5   : DLDNYDADFIGQGKKIITKGVVDSNEEDGGDEDEEAVEGD-EA-----EAGEEG------EEEATNEVASHQSDSEASGESDPASGELAPATARGENATDSPTSQEPSQT : 202
AMA3   : DLDNYDADFIGQGKKIITKGVVDSNEEDGGDEDEEAVEGD-EA-----EAGEEG------EEEATNEVASHQSDSEASGESDPASGELAPATARGENATDSPTSQEPSQT : 202
AND3   : DLDNYDADFIGQGKKIITKGVVDSNEEDGGDEDEEAVEGD-EA-----EAGEEG------EEEATNEVASHQSDSEASGESDPASGELAPATARGENATDSPTSQEPSQT : 202
ORI4   : DLDNYDADFIGQGKKIITKGAVDSNEEDGGDEDEEAVEGD-EA-----EAGEEG------EEEATNEVASHQSDSEASGESDPASGEVAPATARGENATDSPTSQEPSQT : 202
CAR1   : DLDNYDADFIGQGKKIITKGAVDSNEEDGGDEDEEAVEGD-EA-----EAGEEG------EEEATNEVASHQSDSEASGESDPASGEVAPATARGENATDSPTSQEPSQT : 202
AND8   : DLDNYDADFIGQGKKIITKGAVDSNEEDGGDEDEEAVEGD-EA-----EAGEEG------EEEATNEVASHQSDSEASGESDPASGEVAPATARGENATDSPTSQEPSQT : 202
ORI5   : DLDNYDADFIGQGKKIITKGAVDSNEEDGGDEDEEAVEGD-EA-----EAGEEG------EEEATNEVASHQSDSEASGESDPASGEVAPATARGENATDSPTSQEPSQT : 202
PAC7   : DLDNYDADFIGQGKKIITKGAVDSNEEDGGDEDEEAVEGD-EA-----EAGEEG------EEEATNEVASHQSDSAVSPRSVSESREPATATVPAETARDNPPSQETAPT : 202
PAC2   : DLDNYDADFIGQGKKIITKGAVDSNEEDGGDEDEEAVEGD-EA-----EAGEEG------EEEATNEVASHQSDSAVSPRSVSESREPATATAPGETARDNPPSQETAPT : 202
PAC8   : DLDNYDADFIGQGKKIITKGAASSEEGEED---EEDDED--------GEAGQET----------DKGSTSDQPDSAVSPQRAPESPELTTATARGATVRDSPTSPGLSPT : 193
CAR7   : DLDNYDADFIGQGKKIITKGAASSEEGEED---EEDDED--------GEAGQET----------DKGSTSDQPDSAVSPQRAPESPELTTATARGATVRDSPTSPGLSPT : 193
AMA4   : DLDNYDADFIGQGKKIITKGAASSEEGEED---EEDDED--------GEAGQET----------DKGSTSDQPDSAVSPQRAPESPELTTATARGATVRDSPTSPGLSPT : 193
AND4   : DLDNYDADFIGQGKKIITKGAASSEEGEED---EEDDED--------GEAGQET----------DKGSTSDQPDSAVSPQRAPESPELTTATARGATVRDSPTSPGLSPT : 193
         dLDNYDADFIGQGKKIITKGa d  Ee   dedEEavEgd        eag         e ea     ShQs S     s p S E apA a get     tSq psqT
```

```
                  *       240         *       260         *       280         *       300         *       320         *
Sal-I  : HNPGSSGNAEPNSASARTPPAAAEAPLPQPASPDVSTSGEESPNGRVPNVKYLDKLYDEVLKTTDAKDEIHVPPFHSKYNDFRKKYEFTMNEREYQIVKNLFDAFFKKDG : 319
AND11  : HNPGSSGNAEPNSASARTPPAAAEAPLPQPASPDVSTSGEESPNGRVPNVKYLDKLYDEVLKTTDAKDEIHVPPFHSKYNDFRKKYEFTMNEREYQIVKNLFDAFFKKDG : 313
CAR12  : HTTGSSRNVGQSSTSSINPPPAPAAALPQSAPQVVSTPEGEPESGRVPNVKYLDKLYDEVLKTTDAKDEIHVPPFHSKYNDFRKKYEFTMNEREYQIVKNLFDAFFKKDG : 315
AND6   : HTTGSSRNVGQSSTSSINPPPAPAAALPQSAPQVVSTPEGEPESGRVPNVKYLDKLYDEVLKTTDAKDEIHVPPFHSKYNDFRKKYEFTMNEREYQIVKNLFDAFFKKDG : 315
AMA5   : HNPGSSRNEGQSSTSSINPPPAPAAALPQSAPQVVSTPEGEPESGRVPNVKYLDKLYDEVLKTTDAKDEIHVPPFHSKYNDFRKKYEFTMNEREYQIVKNLFDAFFKKDG : 317
AND1   : HNPGSSRNEGQSSTSSINPPPAPAAALPQSAPQVVSTPEGEPESGRVPNVKYLDKLYDEVLKTTDAKDEIHVPPFHSKYNDFRKKYEFTMNEREYQIVKNLFDAFFKKDG : 315
AND5   : HNPGSSRNEGQSSTSSINPPPAPAAALPQSAPQVVSTPEGEPESGRVPNVKYLDKLYDEVLKTTDAKDEIHVPPFHSKYNDFRKKYEFTMNEREYQIVKNLFDAFFKKDG : 313
CAR11  : HNPGSSRNEGQSSTSSINPPPAPAAALPQSAPQVVSTPEGEPESGRVPNVKYLDKLYDEVLKTTDAKDEIHVPPFHSKYNDFRKKYEFTMNEREYQIVKNLFDAFFNKDG : 313
AND12  : HNPGSSRNEGQSSTSSINPPPAPAAALPQSAPQVVSTPEGEPESGRVPNVKYLDKLYDEVLKTTDAKDEIHVPPFHSKYNDFRKKYEFTMNEREYQIVKNLFDAFFNKDG : 313
ORI3   : HNPGSSGNAEPNSASARTPPAAAEAPLPQPASPDVSTSGEESPNGRVPNVKYLDKLYDEVLKTTDAKDEIHVPPFHSKYNDFRKKYEFTMNEREYQIVKNLFDAFFKKDG : 313
AND9   : HNPGSSGNAEPNSASARTPPAAAEAPLPQPASPDVSTSGEESPNGRVPNVKYLDKLYDEVLKTTDAKDEIHVPPFHSKYNDFRKKYEFTMNEREYQIVKNLFDAFFKKDG : 313
PAC6   : HNPGSSGNAEPNSASARTPPAAAEAPLPQPASPDVSTSGEESPNGRVPNVKYLDKLYDEVLKTTDAKDEIHVPPFHSKYNDFRKKYEFTMNEREYQIVKNLFDAFFKKDG : 313
VCG-I  : HNPGSSGNAEPNSASARTPPAAAEAPLPQPASPDVSTSGEESPNGRVPNVKYLDKLYDEVLKTTDAKDEIHVPPFHSKYNDFRKKYEFTMNEREYQIVKNLFDAFFKKDG : 313
PAC1   : HNPGSSGNAEPNSASARTPPAAAEAPLPQPASPDVSTSGEESPNGRVPNVKYLDKLYDEVLKTTDAKDEIHVPPFHSKYNDFRKKYEFTMNEREYQIVKNLFDAFFKKDG : 313
ORI2   : HNPGSSGNAEPNSASARTPPAAAEAPLPQPASPDVSTSGEESPNGRVPNVKYLDKLYDEVLKTTDAKDEIHVPPFHSKYNDFRKGYEFTMNEREYQIVKNLFDAFFNKDG : 315
CAR2   : HNPGSSGNAEPNSASARTPPAAAEAPLPQPASPDVSTSGEESPNGRVPNVKYLDKLYDEVLKTTDAKDEIHVPPFHSKYNDFRKKYEFTMNERECQIVKNLFDACFNKDG : 313
CAR4   : HNPGSSGNAEPNSASARTPPAAAEAPLPQPASPDVSTSGEESPNGRVPNVKYLDKLYDEVLKTTDAKDEIHVPPFHSKYNDFRKKYEFTMNEREYQIVKNLFDAFFKKDG : 313
ORI1   : HNPGSSGNAEPNSASARTPPAAAEAPLPQPASPDVSTSGEESPNGRVPNVKYLDKLYDEVLKTTDAKDEIHVPPFHSKYNDFRKKYEFTMNEREYQIVKNLFDAFFKKDG : 313
AND7   : HNPGSSGNAEPNSASARTPPAAAEAPLPQPASPDVSTSGEESPNGRVPNVKYLDKLYDEVLKTTDAKDEIHVPPFHSKYNDFRKKYEFTMNEREYQIVKNLFDAFFKKDG : 313
AND2   : HNPGSSGNAEPNSASARTPPAAAEAPLPQPASPDVSTSGEESPNGRVPNVKYLDKLYDEVLKTTDAKDEIHVPPFHSKYNDFRKKYEFTMNEREYQIVKNLFDAFFKKDG : 313
CAR5   : HNPGSSGNAEPNSASARTPPAAAEAPLPQPASPDVSTSGEESPNGRVPNVKYLDKLYDEVLKTTDAKDEIHVPPFHSKYNDFRKKYEFTMNEREYQIVKNLFDAFFKKDG : 313
CAR6   : HNPGSSGNAEPNSASARTPPAAAEAPLPQPASPDVSTSGEESPNGRVPNVKYLDKLYDEVLKTTDAKDEIHVPPFHSKYNDFRKKYEFTMNEREYQIVKNLFDAFFKKDG : 313
CAR3   : HNPGSSGNAEPNSASARTPPAAAEAPLPQPASPDVSTSGEESPNGRVPNVKYLDKLYDEVLKTTDAKDEIHVPPFHSKYNDFRKKYEFTMNEREYQIVKNLFDAFFKKDG : 313
AND13  : HNPGSSRNEGQSSTSSINPPPAPAAALPQSAPQVVSTPEGEPESGRVPNVKYLDKLYDEVLKTTDAKDEIHVPPFHSKYNDFRKKYEFTMNEREYQIVKNLFDAFFNKDG : 313
AND10  : HNPGSSRNEGQSSTSSINPPPAPAAALPQSAPQVVSTPEGEPESGRVPNVKYLDKLYDEVLKTTDAKDEIHVPPFHSKYNDFRKKYEFTMNEREYQIVKNLFDAFFNKDG : 313
CAR13  : HNPGSSRNEGQSSTSSINPPPAPAAALPQSAPQVVSTPEGEPESGRVPNVKYLDKLYDEVLKTTDAKDEIHVPPFHSKYNDFRKKYEFTMNEREYQIVKNLFDAFFNKDG : 313
PAC3   : HNPGSSGNAEPNSASARTPPAAAEAPLPQPASPDVSTSGEESPNGRVPNVKYLDKLYDEVLKTTDAKDEIHVPPFHSKYNDFRKKYEFTMNEREYQIVKNLFDAFFKKDG : 313
CAR10  : HNPGSSGNAEPNSASARNPPAAPAAPLPQPASPDVSTSGEESPNGRVPNVKYLDKLYDEVLKTTDAKDEIHVPPFHSKYNDFRKKYEFTMNEREYQIVKNLFDAFFKKDG : 320
CAR8   : HTTGSSRNEGQSSTSSINPPPAPAAALPQSAPQVVSTPEGEPESGRVPNVKYLDKLYDEVLKTTDAKDEIHVPPFHSKYNDFRKKYEFTMNEREYQIVKNLFDAFFKKDG : 312
PAC4   : HTTGSSRNVGQSSTSSINPPPAPAAALPQSAPQVVSTPEGEPESGRVPNVKYLDKLYDEVLKTTDAKDEIHVPPFHSKYNDFRKKYEFTMNEREYQIVKNLFDAFFKKDG : 312
PAC5   : HTTGSSRNVGQSSTSSINPPPAPAAALPQSAPQVVSTPEGEPESGRVPNVKYLDKLYDEVLKTTDAKDEIHVPPFHSKYNDFRKKYEFTMNEREYQIVKNLFDAFFKKDG : 312
AMA3   : HTTGSSRNEGQSSTSSINPPPAPAAALPQSAPQVVSAPEGEPESGRVPNVKYLDKLYDEVLKTTDAKDEIHVPPFHSKYNDFRKGYEFTMNEREYQIVKNLFDAFFNKDG : 312
AND3   : HTTGSSRNEGQSSTSSINPPPAPAAALPQSAPQVVSTPEGEPESGRVPNVKYLDKLYDEVLKTTDAKDEIHVPPFHSKYNDFRKKYEFTMNEREYQIVKNLFDAFFKKDG : 312
ORI4   : HTTGSSRNVGQSSTSSINPPPAPAAALPQSAPQVVSTPEGEPESGRVPNVKYLDKLYDEVLKTTDAKDEIHVPPFHSKYNDFRKKYEFTMNEREYQIVKNLFDAFFKKDG : 312
CAR1   : HTTGSSRNVGQSSTSSINPPPAPAAALPQSAPQVVSTPEGEPESGRVPNVKYLDKLYDEVLKTTDAKDEIHVPPFHSKYNDFRKKYEFTMNEREYQIVKNLFDAFFKKDG : 312
AND8   : HTTGSSRNVGQSSTSSINPPPAPAAALPQSAPQVVSTPEGEPESGRVPNVKYLDKLYDEVLKTTDAKDEIHVPPFHSKYNDFRKKYEFTMNEREYQIVKNLFDAFFKKDG : 312
ORI5   : HTTGSSRNEGQSSTSSINPPPAPAAALPQSAPQVVSTPEGEPESGRVPNVKYLDKLYDEVLKTTDAKDEIHVPPFHSKYNDFRKKYEFTMNEREYHIVKNLFDAFFNKDG : 312
PAC7   : NPPGPSGNVEPNSASARNPPAAAAAAALPQPASPVVSAPEGEPESGRVPNVKYLDKLYDEVLKTTDAKDEIHVPPFHSKYNDFRKGYEFTMNERECQIVKNLFDACFNKDG : 312
PAC2   : NPPGPSGNVEPNSASARNPPAAAAAAALPQPASPVVSAPEGEPESGRVPNVKYLDKLYDEVLKTTDAKDEIHVPPFHSKYNDFRKGYEFTMNERECQIVKNLFDACFKKDG : 312
PAC8   : NPSGQSGNVGQSSTSSINPPPAPAAALPQSAPQVVSTPEGEPESGRVPNVKYLDKLYDEVLKTTDAKDEIHVPPFHSKYNDFRKKYEFTMNEREYQIVKNLFDAFFKKDG : 303
CAR7   : NPSGQSGNVGQSSTSSINPPPAPAAALPQSAPQVVSTPEGEPESGRVPNVKYLDKLYDEVLKTTDAKDEIHVPPFHSKYNDFRKKYEFTMNEREYQIVKNLFDAFFKKDG : 303
AMA4   : NPSGQSGNVGQSSTSSINPPPAPAAALPQSAPQVVSTPEGEPESGRVPNVKYLDKLYDEVLKTTDAKDEIHVPPFHSKYNDFRKGYEFTMNEREYQIVKNLFDAFFNKDG : 303
AND4   : NPSGQSGNVGQSSTSSINPPPAPAAALPQSAPQVVSTPEGEPESGRVPNVKYLDKLYDEVLKTTDAKDEIHVPPFHSKYNDFRKGYEFTMNEREYQIVKNLFDAFFNKDG : 303
         h  GsS N    S S   PP A  A LPQ A   VSt   E   GRVPNVKYLDKLYDEVLKTTDAKDEIHVPPFHSKYNDFRKkYEFTMNEREyqIVKNLFDAfF KDG
```

```
                 340       *         360        *         380        *
Sal-I  : NPSPADAVSFFKKMLNDPNVQKEFDNFVHGLYGFAKRHNYLRDERMNLTDAHKSLFVNTLSLLDTL : 385
AND11  : NPSPADAVSFFKKMLNDPNVQKEFDNFVHGLYGFAKRHNYLRDERMNLTDAHKSLFVNTLSLLDTL : 379
CAR12  : NPSPADAVSFFKKMLNDPNVQKEFDNFVHGLYGFAKRHNYLRDERMNLTDAHKSLFVNTLSLLDTL : 381
AND6   : NPSPADAVSFFKKMLNDPNVQKEFDNFVHGLYGFAKRHNYLRDERMNLTDAHKSLFVNTLSLLDTL : 381
AMA5   : NPSPADAVSFFKKMLNDPNVQKEFDNFVHGLYGFAKRHNYLRDERMNLTDAHKSLFVNTLSLLDTL : 383
AND1   : NPSPADAVSFFKKMLNDPNVQKEFDNFVHGLYGFAKRHNYLRDERMNLTDAHKSLFVNTLSLLDTL : 381
AND5   : NPSPADAVSFFKKMLNDPNVQKEFDNFVHGLYGFAKRHNYLRDERMNLTDAHKSLFVNTLSLLDTL : 379
CAR11  : NPSPADAVSFFKKMLNDPNVQKEFDNFVHGLYGFAKRHNYLRDERMNLTDAHKSLFVNTLSLLDTL : 379
AND12  : NPSPADAVSFFKKMLNDPNVQKEFDNFVHGLYGFAKRHNYLRDERMNLTDAHKSLFVNTLSLLDTL : 379
ORI3   : NPSPADAVSFFKKMLNDPNVQKEFDNFVHGLYGFAKRHNYLRGERMNLTDAHKSLFVNTLSLLDTL : 379
AND9   : NPSPADAVSFFKKMLNDPNVQKEFDNFVHGLYGFAKRHNYLRGERMNLTDAHKSLFVNTLSLLDTL : 379
PAC6   : NPSPADAVSFFKKMLNDPNVQKEFDNFVHGLYGFAKRHNYLRDERMNLTDAHKSLFVNTLSLLDTL : 379
VCG-I  : NPSPADAVSFFKKMLNDPNVQKEFDNFVHGLYGFAKRHNYLRDERMNLTDAHKSLFVNTLSLLDTL : 379
PAC1   : NPSPADAVSFFKKMLNDPNVQKEFDNFVHGLYGFAKRHNYLRDERMNLTDAHKSLFVNTLSLLDTL : 379
ORI2   : NPSPADAVSFFKKMLNDPNVQKEFDNFVHGLYGFAKRHNYLRDERMNLTDAHKSLFVNTLSLLDTL : 381
CAR2   : NPSPADAVSFFKKMLNDPNVQKEFDNFVHGLYGFAKRHNYLRDERMNLTDAHKSLFVNTLSLLDTL : 379
CAR4   : NPSPADAVSFFKKMLNDPNVQKEFDNFVHGLYGFAKRHNYLRDERMNLTDAHKSLFVNTLSLLDTL : 379
ORI1   : NPSPADAVSFFKKMLNDPNVQKEFDNFVHGLYGFAKRHNYLRDERMNLTDAHKSLFVNTLSLLDTL : 379
AND7   : NPSPADAVSFFKKMLNDPNVQKEFDNFVHGLYGFAKRHNYLRDERMNLTDAHKSLFVNTLSLLDTL : 379
AND2   : NPSPADAVSFFKKMLNDPNVQKEFDNFVHGLYGFAKRHNYLRDERMNLTDAHKSLFVNTLSLLDTL : 379
CAR5   : NPSPADAVSFFKKMLNDPNVQKEFDNFVHGLYGFAKRHNYLRDERMNLTDAHKSLFVNTLSLLDTL : 379
CAR6   : NPSPADAVSFFKKMLNDPNVQKEFDNFVHGLYGFAKRHNYLRDERMNLTDAHKSLFVNTLSLLDTL : 379
CAR3   : NPSPADAVSFFKKMLNDPNVQKEFDNFVHGLYGFAKRHNYLRGERMNLTDAHKSLFVNTLSLLDTL : 379
AND13  : NPSPADAVSFFKKMLNDPNVQKEFDNFVHGLYGFAKRHNYLRDERMNLTDAHKSLFVNTLSLLDTL : 379
AND10  : NPSPADAVSFFKKMLNDPNVQKEFDNFVHGLYGFAKRHNYLRDERMNLTDAHKSLFVNTLSLLDTL : 379
CAR13  : NPSPADAVSFFKKMLNDPNVQKEFDNFVHGLYGFAKRHNYLRDERMNLTDAHKSLFVNTLSLLDTL : 379
PAC3   : NPSPADAVSFFKKMLNDPNVQKEFDNFVHGLYGFAKRHNYLRDERMNLTDAHKSLFVNTLSLLDTL : 379
CAR10  : NPSPADAVSFFKKMLNDPNVQKEFDNFVHGLYGFAKRHNYLRDERMNLTDAHKSLFVNTLSLLDTL : 386
CAR8   : NPSPADAVSFFKKMLNDPNVQKEFDNFVHGLYGFAKRHNYLRGERMNLTDAHKSLFVNTLSLLDTL : 378
PAC4   : NPSPADAVSFFKKMLNDPNVQKEFDNFVHGLYGFAKRHNYLRDERMNLTDAHKSLFVNTLSLLDTL : 378
PAC5   : NPSPADAVSFFKKMLNDPNVQKEFDNFVHGLYGFAKRHNYLRDERMNLTDAHKSLFVNTLSLLDTL : 378
AMA3   : NPSPADAVSFFKKMLNDPNVQKEFDNFVHGLYGFAKRHNYLRDERMNLTDAHKSLFVNTLSLLDTL : 378
AND3   : NPSPADAVSFFKKMLNDPNVQKEFDNFVHGLYGFAKRHNYLRDERMNLTDAHKSLFVNTLSLLDTL : 378
ORI4   : NPSPADAVSFFKKMLNDPNVQKEFDNFVHGLYGFAKRHNYLRDERMNLTDAHKSLFVNTLSLLDTL : 378
CAR1   : NPSPADAVSFFKKMLNDPNVQKEFDNFVHGLYGFAKRHNYLRDERMNLTDAHKSLFVNTLSLLDTL : 378
AND8   : NPSPADAVSFFKKMLNDPNVQKEFDNFVHGLYGFAKRHNYLRDERMNLTDAHKSLFVNTLSLLDTL : 378
ORI5   : NPSPADAVSFFKKMLNDPNVQKEFDNFVHGLYGFAKRHNYLRDERMNLTDAHKSLFVNTLSLLDTL : 378
PAC7   : NPSPADAVSFFKKMLNDPNVQKEFDNFVHGLYGFAKRHNYLRDERMNLTDAHKSLFVNTLSLLDTL : 378
PAC2   : NPSPADAVSFFKKMLNDPNVQKEFDNFVHGLYGFAKRHNYLRDERMNLTDAHKSLFVNTLSLLDTL : 378
PAC8   : NPSPADAVSFFKKMLNDPNVQKEFDNFVHGLYGFAKRHNYLRDERMNLTDAHKSLFVNTLSLLDTL : 369
CAR7   : NPSPADAVSFFKKMLNDPNVQKEFDNFVHGLYGFAKRHNYLRGERMNLTDAHKSLFVNTLSLLDTL : 369
AMA4   : NPSPADAVSFFKKMLNDPNVQKEFDNFVHGLYGFAKRHNYLRDERMNLTDAHKSLFVNTLSLLDTL : 369
AND4   : NPSPADAVSFFKKMLNDPNVQKEFDNFVHGLYGFAKRHNYLRDERMNLTDAHKSLFVNTLSLLDTL : 369
         NPSPADAVSFFKKMLNDPNVQKEF1NFVHGLYGFAKRHNYLRdERMNLTDAHKSLFVNTLSLLDTL
```
