# Supplementary material for: Genetic Diversity and Selection in Three Plasmodium vivax Merozoite Surface Protein 7 (Pvmsp-7) Genes in a Colombian Population
Source: PLoS One. 2012 Sep 25;7(9):e45962. doi: 10.1371/journal.pone.0045962 (PMC3458108; doi:10.1371/journal.pone.0045962)
Supplement: Figure S13 — Alignment of the paralogous PvMSP-7deduced amino acid sequences. (PDF) [file pone.0045962.s013.pdf]

```
                            *        20         *        40         *        60         *        80         *       100
AMA4_H   : -MKKKIVLFGSLFVLLSCSTVSSEKLGIQKKKKNLEQDAMHALMKKLESLYKLSATDNSEIFNKEMEALKKQIDQLQQHGGANEEESLGHLLESEAANESTKKTIFGVD : 108
AND4_H   : -MKKKIVLFGSLFVLLSCSTVSSEKLGIQKKKKNLEQDAMHALMKKLESLYKLSATDNSEIFNKEMEALKKQIDQLQQHGGANEEESLGHLLESEAANESTKKTIFGVD : 108
AND1_H   : -MKKKIVLFGSLFVLLSCSTVSSEKLGIQKKKKNLEQDAMHALMKKLESLYKLSATDNSEIFNKEMEALKKQIDQLHQHGGANEEESLGHLLESEAANESTKKTIFGVD : 108
PAC2_H   : -MKKKIVLFGSLFVLLSCSTVSSEKLGIQKKKKNLEQDAMHALMKKLESLYKLSATDNSEIFNKEMEALKKQIDQLQQHGGANEEESLGHLLESEDADDSGKKTIFGVD : 108
PAC3_H   : -MKKKIVLFGSLFVLLSCSTVSSEKLGIQKKKKNLEQDATHALMKKLESLYKLSATDNSEIFNKEIESLKKQIDQLHQHGGENEGESLGHLLESEAANESTKKTIFGVD : 108
CAR5_H   : -MKKKIVLFGSLFVLLSCSTVSSEKLGIQKKKKNLEQDATHALMKKLESLYKLSATDNSEIFNKEIESLKKQIDQLHQHGGENEGESLGHLLESEAANESAKKTIFGVD : 108
AMA3_H   : -MKKKIVLFGSLFVLLSCSTVSSEKLGIQKKKKNLEQDAMHALMKKLESLYKLSATDNSEIFNKEMEALKKQIDQLQQHGGANEEESLGHLLESEAANESTKKTIFGVD : 108
AND8_H   : -MKKKIVLFGSLFVLLSCSTVSSEKLGIQKKKKNLEQDAMHALMKKLESLYKLSATDNSEIFNKEMEALKKQIDQLQQHGGANEEESLGHLLESEAANESTKKTIFGVD : 108
AND11_H  : -MKKKIVLFGSLFVLLSCSTVSSEKLGIQKKKKNLEQDAMHALMKKLESLYKLTATDNSEIFNKEMEALKKQIDQLQQHGGANEEESLGHLLESEAANESTKKTIFGVD : 108
AND9_H   : -MKKKIVLFGSLFVLLSCSTVSSEKLGIQKKKKNLEQDAMHALMKKLESLYKLSATDNSEIFNKEMEALKKQIDQLQQHGGANEEESLGHLLESEAANESTKKTIFGVD : 108
CAR3_H   : -MKKKIVLFGSLFVLLSCSTVSSEKLGIQKKKKNLEQDAMHALMKKLESLYKLSATDNSEIFNKEMEALKKQIDQLQQHGGANEEESLGHLLESEAANESTKKTIFGVD : 108
CAR6_H   : -MKKKIVLFGSLFVLLSCSTVSSEKLGIQKKKKNLEQDAMHALMKKLESLYKLSATDNSEIFNKEMEALKKQIDQLQQHGGANEEESLGHLLESEAANESTKKTIFGVD : 108
SalI_H   : -MKKKIVLFGSLFVLLSCSTVSSEKLGIQKKKKNLEQDATHALMKKLESLYKLSATDNSEIFNKEIESLKKQIDQLHQHGGENEGESLGHLLESEAANESTKKTIFGVD : 108
AND5_H   : -MKKKIVLFGSLFVLLSCSTVSSEKLGIQKKKKNLEQDATHALMKKLESLYKLSATDNSEIFNKEIESLKKQIDQLHQHGGENEGESLGHLLESEAANESTKKTIFGVD : 108
CAR1_H   : -MKKKIVLFGSLFVLLSCSTVSSEKLGIQKKKKNLEQDATHALMKKLESLYKLSATDNSEIFNKEIESLKKQIDQLHQHGGENEGESLGHLLESEAANESTKKTIFGVD : 108
CAR2_H   : -MKKKIVLFGSLFVLLSCSTVSSEKLGIQKKKKNLEQDATHALMKKLESLYKLSATDNSEIFNKEIESLKKQIDQLHQHGGENEGESLGHLLESEAANESTKKTIFGVD : 108
AND3_H   : -MKKKIVLFGSLFVLLSCSTVSSEKLGIQKKKKNLEQDAMHALMKKLESLYKLSATDNSEIFNKEMEALKKQIDQLQQHGGANEEESLGHLLESEAANESTKKTIFGVD : 108
CAR8_H   : -MKKKIVLFGSLFVLLSCSTVSSEKLGIQKKKKNLEQDAMHALMKKLESLYKLSATDNSEIFNKEMEALKKQIDQLQQHGGANEEESLGHLLESEAANESTKKTIFGVD : 108
ORI1_H   : -MKKKIVLFGSLFVLLSCSTVSSEKLGIQKKKKNLEQDAMHALMKKLESLYKLSATDNSEIFNKEMEALKKQIDQLQQHGGANEEESLGHLLESEAANESTKKTIFGVD : 108
AND10_H  : -MKKKIVLFGSLFVLLSCSTVSSEKLGIQKKKKNLEQDAMHALMKKLESLYKLSATDNSEIFNKEMEALKKQIDQLQQHGGANEEESLGHLLESEAANESTKKTIFGVD : 108
AND13_H  : -MKKKIVLFGSLFVLLSCSTVSSEKLGIQKKKKNLEQDAMHALMKKLESLYKLSATDNSEIFNKEMEALKKQIDQLQQHGGANEEESLGHLLESEAANESTKKTIFGVD : 108
CAR11_H  : -MKKKIVLFGSLFVLLSCSTVSSEKLGIQKKKKNLEQDAMHALMKKLESLYKLSATDNSEIFNKEMEALKKQIDQLQQHGGANEEESLGHLLESEAANESTKKTIFGVD : 108
CAR13_H  : -MKKKIVLFGSLFVLLSCSTVSSEKLGIQKKKKNLEQDAMHALMKKLESLYKLSATDNSEIFNKEMEALKKQIDQLQQHGGANEEESLGHLLESEAANESTKKTIFGVD : 108
AND12_H  : -MKKKIVLFGSLFVLLSCSTVSSEKLGIQKKKKNLEQDAMHALMKKLESLYKLSATDNSEIFNKEMEALKKQIDQLQQHGGANEEESLGHLLESEAANESTKKTIFGVD : 108
AND7_H   : -MKKKIVLFGSLFVLLSCSTVSSEKLGIQKKKKNLEQDAMHALMKKLESLYKLSATDNSEIFNKEMEALKKQIDQLQQHGGANEEESLGHLLESEAANESTKKTIFGVD : 108
AND2_H   : -MKKKIVLFGSLFVLLSCSTVSSEKLGIQKKKKNLEQDAMHALMKKLESLYKLSATDNSEIFNKEMEALKKQIDQLQQHGGANEEESLGHLLESEAANESTKKTIFGVD : 108
AND14_H  : -MKKKIVLFGSLFVLLSCSTVSSEKLGIQKKKKNLEQDAMHALMKKLESLYKLSATDNSEIFNKEMEALKKQIDQLHQHGGANEEESLGHLLESEAANESTKKTIFGVD : 108
PAC6_H   : -MKKKIVLFGSLFVLLSCSTVSSEKLGIQKKKKNLEQDAMHALMKKLESLYKLSATDNSEIFNKEMEALKKQIDQLHQHGGANEEESLGHLLESEAANESTKKTIFGVD : 108
CAR4_H   : -MKKKIVLFGSLFVLLSCSTVSSEKLGIQKKKKNLEQDAMHALMKKLESLYKLSATDNSEIFNKEMEALKKQIDQLQQHGGANEEESLGHLLESEAANESTKKTIFGVD : 108
VCGI_H   : -MKKKIVLFGSLFVLLSCSTVSSEKLGIQKKKKNLEQDAMHALMKKLESLYKLSATDNSEIFNKEMEALKKQIDQLQQHGGANEEESLGHLLESEAANESTKKTIFGVD : 108
PAC1_H   : -MKKKIVLFGSLFVLLSCSTVSSEKLGIQKKKKNLEQDAMHALMKKLESLYKLSATDNSEIFNKEMEALKKQIDQLQQHGGANEEESLGHLLESEAANESTKKTIFGVD : 108
ORI3_H   : -MKKKIVLFGSLFVLLSCSTVSSEKLGIQKKKKNLEQDAMHALMKKLESLYKLSATDNSEIFNKEMEALKKQIDQLQQHGGANEEESLGHLLESEAANESTKKTIFGVD : 108
PAC5_H   : -MKKKIVLFGSLFVLLSCSTVSSEKLGIQKKKKNLEQDAMHALMKKLESLYKLSATDNSEIFNKEMEALKKQIDQLQQHGGANEEESLGHLLESEAANESTKKTIFGVD : 108
PAC7_H   : -MKKKIVLFGSLFVLLSCSTVSSEKLGIQKKKKNLEQDAMHALMKKLESLYKLSATDNSEIFNKEMEALKKQIDQLQQHGGANEEESLGHLLESEAANESTKKTIFGVD : 108
PAC4_H   : -MKKKIVLFGSLFVLLSCSTVSSEKLGIQKKKKNLEQDAMHALMKKLESLYKLSATDNSEIFNKEMEALKKQIDQLQQHGGANEEESLGHLLESEAANESTKKTIFGVD : 108
AMA2_H   : -MKKKIVLFGSLFVLLSCSTVSSEKLGIQKKKKNLEQDAMHALMKKLESLYKLSATDNSEIFNKEMEALKKQIDQLQQHGGANEEESLGHLLESEAANESTKKTIFGVD : 108
AND6_H   : -MKKKIVLFGSLFVLLSCSTVSSEKLGIQKKKKNLEQDAMHALMKKLESLYKLSATDNSEIFNKEMEALKKQIDQLQQHGGANEEESLGHLLESEAANESTKKTIFGVD : 108
CAR12_H  : -MKKKIVLFGSLFVLLSCSTVSSEKLGIQKKKKNLEQDAMHALMKKLESLYKLSATDNSEIFNKEMEALKKQIDQLQQHGGANEEESLGHLLESEAANESTKKTIFGVD : 108
CAR4_I   : -------MMSSFLFFLSLHTVPCEKLGIQKKKKNLEQDAMHTLMKKLESLYKLSATDNGEIFNKEIDALKKQIDQLQQHGGDNEGESLGHLLENEAADDSGKKTIFGVD : 102
ORI1_I   : -------MMSSFLFFLSLHTVPCEKLGIQKKKKNLEQDAMHTLMKKLESLYKLSATDNGEIFNKEIDALKKQIDQLQQHGGDNEGESLGHLLENEAADDSGKKTIFGVD : 102
AND7_I   : -------MMSSFLFFLSLHTVPCEKLGIQKKKKNLEQDAMHTLMKKLESLYKLSATDNGEIFNKEIDALKKQIDQLQQHGGDNEGESLGHLLENEAADDSGKKTIFGVD : 102
CAR6_I   : -------MMSSFLFFLSLHTVPCEKLGIQKKKKNLEQDAMHTLMKKLESLYKLSATDNGEIFNKEIDALKKQIDQLQQHGGDNEGESLGHLLENEAADDSGKKTIFGVD : 102
SalI_I   : -MRGKYVMMSSFLFFLSLHTVPCEKLGIQKKKKNLEQDAMHALMKKLESLYKLSATDNGEIFNKEIDALKKQIDQLQQHGGDNEGESLGHLLENEAADDSGKKTIFGVD : 108
CAR5_I   : -------MMSSFLFFLSLHTVPCEKLGIQKKKKNLEQDAMHALMKKLESLYKLSATDNGEIFNKEIDALKKQIDQLQQHGGDNEGESLGHLLENEAADDSGKKTIFGVD : 102
```

```
ORI2_I  : -------MMSSFLFFLSLHTVPCEKLGIQKKKKNLEQDAMHALMKKLESLYKLSATDNGEIFNKEIDALKKQIDQLQQHGGDNEGESLGHLLENEAADDSGKKTIFGVD : 102
CAR2_I  : -------MMSSFLFFLSLHTVPCEKLGIQKKKKNLEQDAMHALMKKLESLYKLSATDNGEIFNKEIDALKKQIDQLQQHGGDNEGESLGHLLENEAADDSGKKTIFGVD : 102
AND2_I  : -------MMSSFLFFLSLHTVPCEKLGIQKKKKNLEQDAMHALMKKLESLYKLSATDNGEIFNKEIDALKKQIDQLQQHGGDNEGESLGHLLENEAADDSGKKTIFGVD : 102
PAC3_I  : -------MMSSFLFFLSLHTVPCEKLGIQKKKKNLEQDAMHALMKKLESLYKLSATDNGEIFNKEIDALKKQIDQLQQHGGDNEGESLGHLLENEAADDSGKKTIFGVD : 102
AND11_I : -------MMSSFLFFLSLHTVPCEKLGIQKKKKNLEQDAMHALMKKLESLYKLSATDNGEIFNKEIDALKKQIDQLQQHGGDNEGESLGHLLENEAADDSGKKTIFGVD : 102
PAC6_I  : -------MMSSFLFFLSLHTVPCEKLGIQKKKKNLEQDAMHALMKKLESLYKLSATDNGEIFNKEIDALKKQIDQLQQHGGDNEGESLGHLLENEAADDSGKKTIFGVD : 102
VCGI_I  : -------MMSSFLFFLSLHTVPCEKLGIQKKKKNLEQDAMHALMKKLESLYKLSATDNGEIFNKEIDALKKQIDQLQQHGGDNEGESLGHLLENEAADDSGKKTIFGVD : 102
PAC1_I  : -------MMSSFLFFLSLHTVPCEKLGIQKKKKNLEQDAMHALMKKLESLYKLSATDNGEIFNKEIDALKKQIDQLQQHGGDNEGESLGHLLENEAADDSGKKTIFGVD : 102
ORI3_I  : -------MMSSFLFFLSLHTVPCEKLGIQKKKKNLEQDAMHALMKKLESLYKLSATDNGEIFNKEIDALKKQIDQLQQHGGDNEGESLGHLLENEAADDSGKKTIFGVD : 102
AND9_I  : -------MMSSFLFFLSLHTVPCEKLGIQKKKKNLEQDAMHALMKKLESLYKLSATDNGEIFNKEIDALKKQIDQLQQHGGDNEGESLGHLLENEAADDSGKKTIFGVD : 102
CAR3_I  : -------MMSSFLFFLSLHTVPCEKLGIQKKKKNLEQDAMHALMKKLESLYKLSATDNGEIFNKEIDALKKQIDQLQQHGGDNEGESLGHLLENEAADDSGKKTIFGVD : 102
CAR10_I : -------MMSSFLFFLSLHTVPCEKLGIQKKKKNLEQDAMHALMKKLESLYKLSATDNGEIFNKEIDALKKQIDQLQQHGGDNEGESLGHLLENEAADDSGKKTIFGVD : 102
CAR12_I : -------MMSSFLFFLSLHTVPCEKLGIQKKKKNLEQDAMHALMKKLESLYKLSATDNGEIFNKEIDALKKQIDQLQQHGGDNEGESLGHLLENEAADDSGKKTIFGVD : 102
AND6_I  : -------MMSSFLFFLSLHTVPCEKLGIQKKKKNLEQDAMHALMKKLESLYKLSATDNGEIFNKEIDALKKQIDQLQQHGGDNEGESLGHLLENEAADDSGKKTIFGVD : 102
AMA5_I  : -------MMSSFLFFLSLHTVPCEKLGIQKKKKNLEQDAMHALMKKLESLYKLSATDNGEIFNKEIDALKKQIDQLQQHGGDNEGESLGHLLENEAADDSGKKTIFGVD : 102
AND1_I  : -------MMSSFLFFLSLHTVPCEKLGIQKKKKNLEQDAMHALMKKLESLYKLSATDNGEIFNKEIDALKKQIDQLQQHGGDNEGESLGHLLENEAADDSGKKTIFGVD : 102
AND5_I  : -------MMSSFLFFLSLHTVPCEKLGIQKKKKNLEQDAMHALMKKLESLYKLSATDNGEIFNKEIDALKKQIDQLQQHGGDNEGESLGHLLENEAADDSGKKTIFGVD : 102
CAR11_I : -------MMSSFLFFLSLHTVPCEKLGIQKKKKNLEQDAMHALMKKLESLYKLSATDNGEIFNKEIDALKKQIDQLQQHGGDNEGESLGHLLENEAADDSGKKTIFGVD : 102
AND12_I : -------MMSSFLFFLSLHTVPCEKLGIQKKKKNLEQDAMHALMKKLESLYKLSATDNGEIFNKEIDALKKQIDQLQQHGGDNEGESLGHLLENEAADDSGKKTIFGVD : 102
AND13_I : -------MMSSFLFFLSLHTVPCEKLGIQKKKKNLEQDAMHALMKKLESLYKLSATDNGEIFNKEIDALKKQIDQLQQHGGDNEGESLGHLLENEAADDSGKKTIFGVD : 102
AND10_I : -------MMSSFLFFLSLHTVPCEKLGIQKKKKNLEQDAMHALMKKLESLYKLSATDNGEIFNKEIDALKKQIDQLQQHGGDNEGESLGHLLENEAADDSGKKTIFGVD : 102
CAR13_I : -------MMSSFLFFLSLHTVPCEKLGIQKKKKNLEQDAMHALMKKLESLYKLSATDNGEIFNKEIDALKKQIDQLQQHGGDNEGESLGHLLENEAADDSGKKTIFGVD : 102
PAC4_I  : -------MMSSFLFFLSLHTVPCEKLGIQKKKKNLEQDAMHALMKKLESLYKLSATDNGEIFNKEIDALKKQIDQLQQHGGDNEGESLGHLLENEAADDSGKKTIFGVD : 102
PAC5_I  : -------MMSSFLFFLSLHTVPCEKLGIQKKKKNLEQDAMHALMKKLESLYKLSATDNGEIFNKEIDALKKQIDQLQQHGGDNEGESLGHLLENEAADDSGKKTIFGVD : 102
AND3_I  : -------MMSSFLFFLSLHTVPCEKLGIQKKKKNLEQDAMHALMKKLESLYKLSATDNGEIFNKEIDALKKQIDQLQQHGGDNEGESLGHLLENEAADDSGKKTIFGVD : 102
AMA3_I  : -------MMSSFLFFLSLHTVPCEKLGIQKKKKNLEQDAMHALMKKLESLYKLSATDNGEIFNKEIDALKKQIDQLQQHGGDNEGESLGHLLENEAADDSGKKTIFGVD : 102
ORI4_I  : -------MMSSFLFFLSLHTVPCEKLGIQKKKKNLEQDAMHALMKKLESLYKLSATDNGEIFNKEIDALKKQIDQLQQHGGDNEGESLGHLLENEAADDSGKKTIFGVD : 102
CAR1_I  : -------MMSSFLFFLSLHTVPCEKLGIQKKKKNLEQDAMHALMKKLESLYKLSATDNGEIFNKEIDALKKQIDQLQQHGGDNEGESLGHLLENEAADDSGKKTIFGVD : 102
AND8_I  : -------MMSSFLFFLSLHTVPCEKLGIQKKKKNLEQDAMHALMKKLESLYKLSATDNGEIFNKEIDALKKQIDQLQQHGGDNEGESLGHLLENEAADDSGKKTIFGVD : 102
ORI5_I  : -------MMSSFLFFLSLHTVPCEKLGIQKKKKNLEQDAMHALMKKLESLYKLSATDNGEIFNKEIDALKKQIDQLQQHGGDNEGESLGHLLENEAADDSGKKTIFGVD : 102
CAR8_I  : -------MMSSFLFFLSLHTVPCEKLGIQKKKKNLEQDAMHALMKKLESLYKLSATDNGEIFNKEIDALKKQIDQLQQHGGDNEGESLGHLLENEAADDSGKKTIFGVD : 102
PAC7_I  : -------MMSSFLFFLSLHTVPCEKLGIQKKKKNLEQDAMHALMKKLESLYKLSATDNGEIFNKEIDALKKQIDQLQQHGGDNEGESLGHLLENEAADDSGKKTIFGVD : 102
PAC2_I  : -------MMSSFLFFLSLHTVPCEKLGIQKKKKNLEQDAMHALMKKLESLYKLSATDNGEIFNKEIDALKKQIDQLQQHGGDNEGESLGHLLENEAADDSGKKTIFGVD : 102
AMA4_I  : -------MMSSFLFFLSLHTVPCEKLGIQKKKKNLEQDAMHALMKKLESLYKLSATDNGEIFNKEIDALKKQIDQLQQHGGDNEGESLGHLLENEAADDSGKKTIFGVD : 102
AND4_I  : -------MMSSFLFFLSLHTVPCEKLGIQKKKKNLEQDAMHALMKKLESLYKLSATDNGEIFNKEIDALKKQIDQLQQHGGDNEGESLGHLLENEAADDSGKKTIFGVD : 102
PAC8_I  : -------MMSSFLFFLSLHTVPCEKLGIQKKKKNLEQDAMHALMKKLESLYKLSATDNGEIFNKEIDALKKQIDQLQQHGGDNEGESLGHLLENEAADDSGKKTIFGVD : 102
CAR7_I  : -------MMSSFLFFLSLHTVPCEKLGIQKKKKNLEQDAMHALMKKLESLYKLSATDNGEIFNKEIDALKKQIDQLQQHGGDNEGESLGHLLENEAADDSGKKTIFGVD : 102
AND6_C  : ------IFFLSFSFLISFHSVLSEKLGIQKKKKNLEQDAMHALMKKLESLYKLSATDNSEIFNKEMEALKKQIDQLQQHGGANEEESLGHLLESEAADDSSKKTIFGVD : 103
ORI2_C  : ------IFFLSFSFLISFHSVLSEKLGIQKKKKNLEQDAMHALMKKLESLYKLSATDNSEIFNKEMEALKKQIDQLQQHGGANEEESLGHLLESEAADDSSKKTIFGVD : 103
CAR8_C  : ------IFFLSFSFLISFHSVLSEKLGIQKKKKNLEQDAMHALMKKLESLYKLSATDNSEIFNKEMEALKKQIDQLQQHGGANEEESLGHLLESEAADDSSKKTIFGVD : 103
AND7_C  : ------IFFLSFSFLISFHSVLSEKLGIQKKKKNLEQDAMHALMKKLESLYKLSATDNSEIFNKEMEALKKQIDQLQQHGGANEEESLGHLLESEAADDSSKKTIFGVD : 103
SalI_C  : MNKTIVIFFLSFSFLISFHSVLSEKLGIQKKKKNLEQDAMHALMKKLESLYKLSATDNSEIFNKEMEALKKQIDQLQQHGGANEEESLGHLLESEAADDSSKKTIFGVD : 109
CAR10_C : ------IFFLSFSFLISFHSVLSEKLGIQKKKKNLEQDAMHALMKKLESLYKLSATDNSEIFNKEMEALKKQIDQLQQHGGANEEESLGHLLESEAADDSSKKTIFGVD : 103
CAR11_C : ------IFFLSFSFLISFHSVLSEKLGIQKKKKNLEQDAMHALMKKLESLYKLSATDNSEIFNKEMEALKKQIDQLQQHGGANEEESLGHLLESEAADDSSKKTIFGVD : 103
AND4_C  : ------IFFLSFSFLISFHSVLSEKLGIQKKKKNLEQDAMHALMKKLESLYKLSATDNSEIFNKEMEALKKQIDHLQQHGGANEEESLGHLLESEAADDSSKKTIFGVD : 103
AND5_C  : ------IFFLSFSFLISFHSVLSEKLGIQKKKKNLEQDAMHALMKKLESLYKLSATDNSEIFNKEMEALKKQIDHLQQHGGANEEESLGHLLESEAADDSSKKTIFGVD : 103
```

```
CAR9_C    : ------IFFLSFSFLISFHSVLSEKLGIQKKKKNLEQDAMHALMKKLESLYKLSATDNSEIFNKEMEALKKQIDQLQQHGGANEEESLGHLLESEAANESTKKTIFGVD : 103
AND8_C    : ------IFFLSFSFLISFHSVLSEKLGIQKKKKNLEQDAMHALMKKLESLYKLSATDNSEIFNKEMEALKKQIDQLQQHGGANEEESLGHLLESEAANESTKKTIFGVD : 103
ORI1_C    : ------IFFLSFSFLISFHSVLSEKLGIQKKKKNLEQDAMHALMKKLESLYKLSATDNSEIFNKEMEALKKQIDQLQQHGGANEEESLGHLLESEAANESTKKTIFGVD : 103
AND2_C    : ------IFFLSFSFLISFHSVLSEKLGIQKKKKNLEQDAMHALMKKLESLYKLSATDNSEIFNKEMEALKKQIDQLQQHGGANEEESLGHLLESEAANESTKKTIFGVD : 103
AND3_C    : ------IFFLSFSFLISFHSVLSEKLGIQKKKKNLEQDAMHALMKKLESLYKLSATDNSEIFNKEMEALKKQIDQLQQHGGANEEESLGHLLESEAANESTKKTIFGVD : 103
CAR15_C   : ------IFFLSFSFLISFHSVLSEKLGIQKKKKNLEQDAMHALMKKLESLYKLSATDNSEIFNKEMEALKKQIDQLQQHGGANEEESLGHLLESEAANESTKKTIFGVD : 103
CAR2_C    : ------IFFLSFSFLISFHSVLSEKLGIQKKKKNLEQDAMHALMKKLESLYKLSATDNSEIFNKEMEALKKQIDQLQQHGGANEEESLGHLLESEAANESTKKTIFGVD : 103
CAR4_C    : ------IFFLSFSFLISFHSVLSEKLGIQKKKKNLEQDAMHALMKKLESLYKLSATDNSEIFNKEMEALKKQIDQLQQHGGANEEESLGHLLESEAANESTKKTIFGVD : 103
CAR7_C    : ------IFFLSFSFLISFHSVLSEKLGIQKKKKNLEQDAMHALMKKLESLYKLSATDNSEIFNKEMEALKKQIDQLQQHGGANEEESLGHLLESEAANESTKKTIFGVD : 103
CAR6_C    : ------IFFLSFSFLISFHSVLSEKLGIQKKKKNLEQDAMHALMKKLESLYKLSATDNSEIFNKEMEALKKQIDQLQQHGGANEEESLGHLLESEAANESTKKTIFGVD : 103
CAR5_C    : ------IFFLSFSFLISFHSVLSEKLGIQKKKKNLEQDAMHALMKKLESLYKLSATDNSEIFNKEMEALKKQIDQLQQHGGANEEESLGHLLESEAANESTKKTIFGVD : 103
ORI3_C    : ------IFFLSFSFLISFHSVLSEKLGIQKKKKNLEQDAMHALMKKLESLYKLSATDNSEIFNKEMEALKKQIDQLQQHGGANEEESLGHLLESEAANESTKKTIFGVD : 103
CAR12_C   : ------IFFLSFSFLISFHSVLSEKLGIQKKKKNLEQDAMHALMKKLESLYKLSATDNSEIFNKEMEALKKQIDQLQQHGGANEEESLGHLLESEAADDSSKKTIFGVD : 103
PAC4_C    : ------IFFLSFSFLISFHSVLSEKLGIQKKKKNLEQDAMHALMKKLESLYKLSATDNSEIFNKEMEALKKQIDQLQQHGGANEEESLGHLLESEAADDSSKKTIFGVD : 103
CAR3_C    : ------IFFLSFSFLISFHSVLSEKLGIQKKKKNLEQDAMHALMKKLESLYKLSATDNSEIFNKEMEALKKQIDQLQQHGGANEEESLGHLLESEAADDSSKKTIFGVD : 103
PAC8_C    : ------IFFLSFSFLISFHSVLSEKLGIQKKKKNLEQDAMHALMKKLESLYKLSATDNSEIFNKEMEALKKQIDQLQQHGGANEEESLGHLLESEAADDSSKKTIFGVD : 103
ORI4_C    : ------IFFLSFSFLISFHSVLSEKLGIQKKKKNLEQDAMHALMKKLESLYKLSATDNSEIFNKEMEALKKQIDQLQQHGGANEEESLGHLLESEAANDSSKKTIFGVD : 103
PAC5_C    : ------IFFLSFSFLISFHSVLSEKLGIQKKKKNLEQDAMHALMKKLESLYKLSATDNSEIFNKEMEALKKQIDQLQQHGGANEEESLGHLLESEAADDSSKKTIFGVD : 103
PAC2_C    : ------IFFLSFSFLISFHSVLSEKLGIQKKKKNLEQDATHALMKKLESLYKLSATDNSEIFNKEIESLKKQIDQLHQHGGENEEESLGHLLESEAADDSGKKTIFGVD : 103
PAC6_C    : ------IFFLSFSFLISFHSVLSEKLGIQKKKKNLEQDATHALMKKLESLYKLSATDNSEIFNKEIESLKKQIDQLHQHGGENEEESLGHLLESEAADDSGKKTIFGVD : 103
PAC3_C    : ------IFFLSFSFLISFHSVLSEKLGIQKKKKNLEQDAMHALMKKLESLYKLSATDNSEIFNKEMEALKKQIDQLQQHGGANEEESLGHLLESEAADDSGKKTIFGVD : 103
CAR13_C   : ------IFFLSFSFLISFHSVLSEKLGIQKKKKNLEQDAMHALMKKLESLYKLSATDNSEIFNKEMEALKKQIDQLQQHGGANEEESLGHLLESEAADDSGKKTIFGVD : 103
AMA5_C    : ------IFFLSFSFLISFHSVLSEKLGIQKKKKNLEQDAMHALMKKLESLYKLSATDNSEIFNKEMEALKKQIDQLQQHGGANEEESLGHLLESEAADDSGKKTIFGVD : 103
CAR14_C   : ------IFFLSFSFLISFHSVLSEKLGIQKKKKNLEQDAMHALMKKLESLYKLSATDNSEIFNKEMEALKKQIDQLQQHGGANEEESLGHLLESEAADDSGKKTIFGVD : 103
AMA2_C    : ------IFFLSFSFLISFHSVLSEKLGIQKKKKNLEQDAMHALMKKLESLYKLSATDNSEIFNKEMEALKKQIDQLQQHGGANEEESLGHLLESEAADDSGKKTIFGVD : 103
CAR1_C    : ------IFFLSFSFLISFHSVLSEKLGIQKKKKNLEQDAMHALMKKLESLYKLSATDNSEIFNKEMEALKKQIDQLQQHGGANEEESLGHLLESEAADDSGKKTIFGVD : 103
AND1_C    : ------IFFLSFSFLISFHSVLSEKLGIQKKKKNLEQDAMHALMKKLESLYKLSATDNSEIFNKEMEALKKQIDQLQQHGGANEEESLGHLLESEAADDSGKKTIFGVD : 103
AMA4_C    : ------IFFLSFSFLISFHSVLSEKLGIQKKKKNLEQDAMHALMKKLESLYKLSATDNSEIFNKEMEALKKQIDHLQQHGGANEEESLGHLLESEAADDSGKKTIFGVD : 103
VCGI_C    : ------IFFLSFSFLISFHSVLSEKLGIQKKKKNLEQDAMHALMKKLESLYKLSATDNSEIFNKEMEALKKQIDQLQQHGGANEEESLGHLLESEAADDSGKKTIFGVD : 103
                        S    6S  3V  EKLGIQKKKKNLEQDAmHaLMKKLESLYKL3ATDN EIFNKE6 aLKKQIDqLqQHGG NE ESLGHLLE EaA1 S KKTIFGVD

             *       120         *       140         *       160         *       180         *       200         *       2
AMA4_H    : EDDLDNYDGDFTGQSKGKFKGHSFKAQKKVEGNDENI----------GGVPVTGNSASN-----SQSTGGSGSQNASPTQGSPSDSAQGSQVTNSAGSTG------TLST : 197
AND4_H    : EDDLDNYDGDFTGQSKGKFKGHSFKAQKKVEGNDENI----------GGVPVTGNSASN-----SQSTGGSGSQNASPTQGSPSDSAQGSQVTNSAGSTG------TLST : 197
AND1_H    : EDDLDNYDGDFTGQSKGKFKGHSFKAQKKVEGNDENI----------GGVPVTGNSASN-----SQSTGGSGSQNASPPQGSPSDSAQGSQVTNSAGSTG------TLST : 197
PAC2_H    : EDDLDNYDADFTGQSKGKIKGHSFKAQKKVEGNDENI----------GGVTVTGNSASN-----SQSTGGSGSQNASQPEGNPGGNPQGTQVTNSAGSTG------TLST : 197
PAC3_H    : EDDLDNYDGDFTGQSKGKFKGHSFKAQKKVEGNDENI----------GGVPVTGNSASN-----SQSTGGSGSQSDSSPQGSPGGNPQGTQVTNSAGSTG------TLST : 197
CAR5_H    : EDDLDNYDGDFTGQSKGKFKGHSFKAQKKVEGNDENFAEAFG----GGVPVTGNSASN-----SQSTGGSGSQSDSSPQGSPGGNPQGTQVTNSAGSTG------TLST : 202
AMA3_H    : EDDLDNYDGDFTGQSKGKFKGHSFKAQKKVEGNDENI----------GGVPVTGNSASN-----SQSTGGSGSQNASPTQGSPGGNPQGTQVTNSAGSTG------TLST : 197
AND8_H    : EDDLDNYDGDFTGQSKGKFKGHSFKAQKKVEGNDENI----------GGVPVTGNSASN-----SQSTGGSGSQNASPTQGSPGGNPQGTQVTNSAGSTG------TLST : 197
AND11_H   : EDDLDNYDGDFTGQSKGKFKGHSFKAQKKVEGNDENF----------GGAPVTGNSAS------SQPAGVWGSQSDSPPQGSPGGDAQGTQVTGSAGSTG------TLST : 196
AND9_H    : EDDLDNYDGDFTGQSKGKFKGHSFKAQKKVEGNDENFAEAFG----GGAPVTGNSAS------SQPAGVSGSQSDSPPQGSPGGNPQGTQVTNSAGSTG------TLST : 201
CAR3_H    : EDDLDNYDGDFTGQSKGKFKGHSFKAQKKVEGNDENFAEAFG----GGAPVTGNSAS------SQPAGVSGSQSDSPPQGSPGGNPQGTQVTNSAGSTG------TLST : 201
CAR6_H    : EDDLDNYDGDFTGQSKGKFKGHSFKAQKKVEGNDENFAEAFG----GGAPVTGNSAS------SQPAGVSGSQSDSPPQGSPGGNPQGTQVTNSAGSTG------TLST : 201
SalI_H    : EDDLDNYDGDFTGQSKGKFKGHSFKAQKKVEGNDENI----------GGVPVTGNSASN-----SQSTGGSGSQNASPPQGSPSDSAQGSQVTNSTGST------VTLNA : 197
AND5_H    : EDDLDNYDGDFTGQSKGKFKGHSFKAQKKVEGNDENI----------GGVPVTGNSASN-----SQSTGGSGSQNASPPQGSPSDSAQGSQVTNSTGST------VTLNA : 197
```

```
CAR1_H   : EDDLDNYDGDFTGQSKGKFKGHSFKAQKKVEGNDENI---------GGVPVTGNSASN-----SQSTGGSGSQNASPPQGSPSDSAQGSQVTNSTGST------VTLNA : 197
CAR2_H   : EDDLDNYDGDFTGQSKGKFKGHSFKAQKKVEGNDENI---------GGVPVTGNSASN-----SQSTGGSGSQNASPPQGSPSDSAQGSQVTNSTGST------VTLNA : 197
AND3_H   : EDDLDNYDGDFTGQSKGKFKGHSFKAQKKVEGNDENI---------GGVPVTGNSASN-----SQSTGGSGSQNASPPEGNNGGSEQGPQVTEGT---------VTLNT : 194
CAR8_H   : EDDLDNYDGDFTGQSKGKFKGHSFKAQKKVEGNDENI---------GGVTVTGNSASN-----SQSTGGSGSQNASPPQDNPGGGAQGSPGTGGTGGTG---GTVTLST : 200
ORI1_H   : EDDLDNYDGDFTGQSKGKFKGHSFKAQKKVEGNDENI---------GGVTVTGNSAS------SQSTGDLNSQNPSQPEGNNGGGAQGSPGTGGTG------GTVTLST : 196
AND10_H  : EDDLDNYDGDFTGQSKGKFKGHSFKAQKKVEGNDENI---------GGVTVTGNSAS------SQSTGDLNSQNPSQPEGNNGGGAQGSPGTGGTGGTG---GTVTLST : 199
AND13_H  : EDDLDNYDGDFTGQSKGKFKGHSFKAQKKVEGNDENI---------GGVTVTGNSAS------SQSTGDLNSQNPSQPEGNNGGGAQGSPGTGGTGGTG---GTVTLST : 199
CAR11_H  : EDDLDNYDGDFTGQSKGKFKGHSFKAQKKVEGNDENI---------GGVTVTGNSAS------SQSTGDLNSQNPSQPEGNNGGGAQGSPGTGGTGGTG---GTVTLST : 199
CAR13_H  : EDDLDNYDGDFTGQSKGKFKGHSFKAQKKVEGNDENI---------GGVTVTGNSAS------SQSTGDLNSQNPSQPEGNNGGGAQGSPGTGGTGGTG---GTVTLST : 199
AND12_H  : EDDLDNYDGDFTGQSKGKFKGHSFKAQKKVEGNDENI---------GGVTVTGNSAS------SQSTGDLNSQNPSQPEGNNGGGAQGSPGTGGTGGTG---GTVTLST : 199
AND7_H   : EDDLDNYDGDFTGQSKGKFKGHSFKAQKKVEGNDENI---------GGVTVTGNSAS------SQSTGDLNSQNPSQPEGNNGGGAQGSPGTGGTGGTG---GTVTLST : 199
AND2_H   : EDDLDNYDGDFTGQSKGKFKGHSFKAQKKVEGNDENI---------GGVTVTGNSAS------SQSTGDLNSQNPSQPEGNNGGGAQGSPGTGGTGGTG---GTVTLST : 199
AND14_H  : EDDLDNYDGDFTGQSKGKFKGHSFKAQKKVEGNDENI---------GGVPVTGNSAS------SQSTGDLNSQNPSPPEGNNGGGAQGSPGTGGTG------STVTLST : 196
PAC6_H   : EDDLDNYDGDFTGQSKGKFKGHSFKAQKKVEGNDENI---------GGVPVTGNSAS------SQSTGDLNSQNPSQPEGNNGGGAQGSPGTGGTGGTG---GTVTLST : 199
CAR4_H   : EDDLDNYDGDFTGQSKGKFKGHSFKAQKKVEGNDENI---------GGVTVTGNSAS------SQSTGDLNSQNPSQPEGNNGGGAQGSPGTGGTGGTG---GTVTLST : 199
VCGI_H   : EDDLDNYDGDFTGQSKGKIKGHSFKAQKKVEGNDENI---------GGVTVTGNSAS------SQSTGDLNSQNPSQPEGNNGGGAQGSPGTGGTGGTG---GTVTLST : 199
PAC1_H   : EDDLDNYDGDFTGQSKGKIKGHSFKAQKKVEGNDENI---------GGVTVTGNSAS------SQSTGDLNSQNPSQPEGNNGGGAQGSPGTGGTGGTG---GTVTLST : 199
ORI3_H   : EDDLDNYDGDFTGQSKGKFKGHSFKAQKKVEGNDENI---------GGVTVTGNSAT------SPPSGGGGSKSPSQPQDNPSDSAQGSPGTGGTGGTG---GTETLST : 199
PAC5_H   : EDDLDNYDGDFTGQSKGKFKGHSFKAQKKVEGNDENI---------GGVTVTGNSAT------SPPSGGGGSKSPSQPQDNPSDSAQGSPGTGGTGGTGGTGGTETLST : 202
PAC7_H   : EDDLDNYDGDFTGQSKGKFKGHSFKAQKKVEGNDENI---------GGVTVTGNSAT------SPPSGGGGSESPSQPQDNPSDSAQGSPGTGGTGGTG---GTETLST : 199
PAC4_H   : EDDLDNYDGDFTGQSKGKFKGHSFKAQKKVEGNDENI---------GGVTVTGNSAT------SPPSGGGGSESPSQPQDNPSDSAQGSPGTGGTGGTGGTGGTETLST : 202
AMA2_H   : EDDLDNYDGDFTGQSKGKFKGHSFKAQKKVEGNDENI---------GGVTVTGNSAT------SPPSGGGGSESPSQPQDNPGGGAQGSPSTGGTGGTG---GTVTLST : 199
AND6_H   : EDDLDNYDGDFTGQSKGKFKGHSFKAQKKVEGNDENI---------GGVTVTGNSAT------SPPSGGGGSESPSQPQDNPGGGAQGSPSTGGTGGTG---GTVTLST : 199
CAR12_H  : EDDLDNYDGDFTGQSKGKFKGHSFKAQKKVEGNDENI---------GGVTVTGNSAT------SPPSGGGGSESPSQPQDNPGGGAQGSPSTGGTGGTG---GTVTLST : 199
CAR4_I   : EDDLDNYDADFIGQGKKIITKGAADKEEEEEDDEDEEAVEGD-------EEAGERD----EAEAEAVRGDTSHQSGSGDLTPSPPGSSERAPAVAPGET-----PRVTA : 195
ORI1_I   : EDDLDNYDADFIGQGKKIITKGAADKEEEEEDDEDEEAVEGD-------EEAGERD----EAEAEAVRGDTSHQSGSGDLTPSPPGSSERAPAVAPGET-----PRVTA : 195
AND7_I   : EDDLDNYDADFIGQGKKIITKGAADKEEEEEDDEDEEAVEGD-------EEAGERD----EAEAEAVRGDTSHQSGSGDLTPSPPGSSERAPAVAPGET-----PRVTA : 195
CAR6_I   : EDDLDNYDADFIGQGKKIITKGAADKEEEEEDDEDEEAVEGD-------EEAGERD----EAEAEAVRGDTSHQSGSGDLTPSPPGSSERAPAVAPGET-----PRVTA : 195
SalI_I   : EDDLDNYDADFIGQGKKIITKGAADKEEEEEDDEDEEAVEGD-------EEAGERD----EAEAEAVRGDTSHQSGSGDLTPSPPGSSERAPAVAPGET-----PRVTA : 201
CAR5_I   : EDDLDNYDADFIGQGKKIITKGAADKEEEEEDDEDEEAVEGD-------EEAGERD----EAEAEAVRGDTSHQSGSGDLTPSPPGSSERAPAVAPGET-----PRVTA : 195
ORI2_I   : EDDLDNYDADFIGQGKKIITKGAADKEEEEEDDEDEEAVEGD-------EEAGERDE--AEAEAEAVRGDTSHQSGSGDLTPSPPGSSERAPAVAPGET-----PRVTA : 197
CAR2_I   : EDDLDNYDADFIGQGKKIITKGAADKEEEEEDDEDEEAVEGD-------EEAGERD----EAEAEAVRGDTSHQSGSGDLTPSPPGSSERAPAVAPGET-----PRVTA : 195
AND2_I   : EDDLDNYDADFIGQGKKIITKGAADKEEEEEDDEDEEAVEGD-------EEAGERD----EAEAEAVRGDTSHQSGSGDLTPSPPGSSERAPAVAPGET-----PRVTA : 195
PAC3_I   : EDDLDNYDADFIGQGKKIITKGAADKEEEEEDDEDEEAVEGD-------EEAGERD----EAEAEAVRGDTSHQSGSGDLTPSPPGSSERAPAVAPGET-----PRVTA : 195
AND11_I  : EDDLDNYDADFIGQGKKIITKGAADKEEEEEDDEDEEAVEGD-------EEAGERD----EAEAEAVRGDTSHQSGSGDLTPSPPGSSERAPAVAPGET-----PRDTA : 195
PAC6_I   : EDDLDNYDADFIGQGKKIITKGAADKEEEEEDDEDEEAVEGD-------EEAGERD----EAEAEAVRGDTSHQSGSGDLTPSPPGSSERAPAVAPGET-----PRDTA : 195
VCGI_I   : EDDLDNYDADFIGQGKKIITKGAADKEEEEEDDEDEEAVEGD-------EEAGERD----EAEAEAVRGDTSHQSGSGDLTPSPPGSSERAPAVAPGET-----PRDTA : 195
PAC1_I   : EDDLDNYDADFIGQGKKIITKGAADKEEEEEDDEDEEAVEGD-------EEAGERD----EAEAEAVRGDTSHQSGSGDLTPSPPGSSERAPAVAPGET-----PRDTA : 195
ORI3_I   : EDDLDNYDADFIGQGKKIITKGAADKEEEEEDDEDEEAVEGD-------EEAGERD----EAEAEAVRGDTSHQSGSGDLTPSPPGSSERAPAVAPGET-----PRDTA : 195
AND9_I   : EDDLDNYDADFIGQGKKIITKGAADKEEEEEDDEDEEAVEGD-------EEAGERD----EAEAEAVRGDTSHQSGSGDLTPSPPGSSERAPAVAPGET-----PRDTA : 195
CAR3_I   : EDDLDNYDADFIGQGKKIITKGAADKEEEEEDDEDEEAVEGD-------EEAGERD----EAEAEAVRGDTSHQSGSGDLTPSPPGSSERAPAVAPGET-----PRDTA : 195
CAR10_I  : EDDLDNYDADFIGQGKKIITKGAADKEEEEEDDEDEEAVEGDEEAVEGDEEAGEED----EAEAEAVRGDTSHQSGSGDLTPSPPGSSERAPAVAPGET-----PRVTA : 202
CAR12_I  : EDDLDNYDADFIGQGKKIITKGAADKEEEEEDDEDEEAVEGD-------EEAGERD--EAEAEAEAVRGDTSHQSGSGDLTPSPPGSSERAPAVAPGET-----PRVTA : 197
AND6_I   : EDDLDNYDADFIGQGKKIITKGAADKEEEEEDDEDEEAVEGD-------EEAGERD--EAEAEAEAVRGDTSHQSGSGDLTPSPPGSSERAPAVAPGET-----PRVTA : 197
AMA5_I   : EDDLDNYDADFIGQGKKIITKGAADKEEEEEDDEDEEAVEGD-------EEAGERDEAEAEAEAEAVRGDTSHQSGSGDLTPSPPGSSERAPAVAPGET-----PRVTA : 199
AND1_I   : EDDLDNYDADFIGQGKKIITKGAADKEEEEEDDEDEEAVEGD-------EEAGERD--EAEAEAEAVRGDTSHQSGSGDLTPSPPGSSERAPAVAPGET-----PRVTA : 197
```

```
AND5_I   : EDDLDNYDADFIGQGKKIITKGAADKEEEEEDDEDEEAVEGD-------EEAGERD----EAEAEAVRGDTSHQSGSGDLTPSPPGSSERAPAVAPGET-----PRVTA : 195
CAR11_I  : EDDLDNYDADFIGQGKKIITKGAADKEEEEEDDEDEEAVEGD-------EEAGERD----EAEAEAVRGDTSHQSGSGDLTPSPPGSSERAPAVAPGET-----PRVTA : 195
AND12_I  : EDDLDNYDADFIGQGKKIITKGAADKEEEEEDDEDEEAVEGD-------EEAGERD----EAEAEAVRGDTSHQSGSGDLTPSPPGSSERAPAVAPGET-----PRVTA : 195
AND13_I  : EDDLDNYDADFIGQGKKIITKGAADKEEEEEDDEDEEAVEGD-------EEAGERD----EAEAEAVRGDTSHQSGSGDLTPSPPGSSERAPAVAPGET-----PRVTA : 195
AND10_I  : EDDLDNYDADFIGQGKKIITKGAADKEEEEEDDEDEEAVEGD-------EEAGERD----EAEAEAVRGDTSHQSGSGDLTPSPPGSSERAPAVAPGET-----PRVTA : 195
CAR13_I  : EDDLDNYDADFIGQGKKIITKGAADKEEEEEDDEDEEAVEGD-------EEAGERD----EAEAEAVRGDTSHQSGSGDLTPSPPGSSERAPAVAPGET-----PRVTA : 195
PAC4_I   : EDDLDNYDADFIGQGKKIITKGVVDSNEEDGGDEDEEAVEGDE------AEAGEEG------EEEATNEVASHQSDSEASGESDPASGELAPATARGEN-----ATDSP : 194
PAC5_I   : EDDLDNYDADFIGQGKKIITKGVVDSNEEDGGDEDEEAVEGDE------AEAGEEG------EEEATNEVASHQSDSEASGESDPASGELAPATARGEN-----ATDSP : 194
AND3_I   : EDDLDNYDADFIGQGKKIITKGVVDSNEEDGGDEDEEAVEGDE------AEAGEEG------EEEATNEVASHQSDSEASGESDPASGELAPATARGEN-----ATDSP : 194
AMA3_I   : EDDLDNYDADFIGQGKKIITKGVVDSNEEDGGDEDEEAVEGDE------AEAGEEG------EEEATNEVASHQSDSEASGESDPASGELAPATARGEN-----ATDSP : 194
ORI4_I   : EDDLDNYDADFIGQGKKIITKGAVDSNEEDGGDEDEEAVEGDE------AEAGEEG------EEEATNEVASHQSDSEASGESDPASGEVAPATARGEN-----ATDSP : 194
CAR1_I   : EDDLDNYDADFIGQGKKIITKGAVDSNEEDGGDEDEEAVEGDE------AEAGEEG------EEEATNEVASHQSDSEASGESDPASGEVAPATARGEN-----ATDSP : 194
AND8_I   : EDDLDNYDADFIGQGKKIITKGAVDSNEEDGGDEDEEAVEGDE------AEAGEEG------EEEATNEVASHQSDSEASGESDPASGEVAPATARGEN-----ATDSP : 194
ORI5_I   : EDDLDNYDADFIGQGKKIITKGAVDSNEEDGGDEDEEAVEGDE------AEAGEEG------EEEATNEVASHQSDSEASGESDPASGEVAPATARGEN-----ATDSP : 194
CAR8_I   : EDDLDNYDADFIGQGKKIITKGAVDSNEEDGGDEDEEAVEGDE------AEAGEEG------EEEATNEVASHQSGSGDLTPSPPASGELAPATARGET-----ATDSP : 194
PAC7_I   : EDDLDNYDADFIGQGKKIITKGAVDSNEEDGGDEDEEAVEGDE------AEAGEEG------EEEATNEVASHQSDSAVSPRSVSESREPATATVPAET-----ARDNP : 194
PAC2_I   : EDDLDNYDADFIGQGKKIITKGAVDSNEEDGGDEDEEAVEGDE------AEAGEEG------EEEATNEVASHQSDSAVSPRSVSESREPATATAPGET-----ARDNP : 194
AMA4_I   : EDDLDNYDADFIGQGKKIITKGAASSEE---GEEDEEDDEDG--------EAGQET----------DKGSTSDQPDSAVSPQRAPESPELTTATARGAT-----VRDSP : 185
AND4_I   : EDDLDNYDADFIGQGKKIITKGAASSEE---GEEDEEDDEDG--------EAGQET----------DKGSTSDQPDSAVSPQRAPESPELTTATARGAT-----VRDSP : 185
PAC8_I   : EDDLDNYDADFIGQGKKIITKGAASSEE---GEEDEEDDEDG--------EAGQET----------DKGSTSDQPDSAVSPQRAPESPELTTATARGAT-----VRDSP : 185
CAR7_I   : EDDLDNYDADFIGQGKKIITKGAASSEE---GEEDEEDDEDG--------EAGQET----------DKGSTSDQPDSAVSPQRAPESPELTTATARGAT-----VRDSP : 185
AND6_C   : EDDLDNYDGDFTGQSKGKIKGQADTAPS-VKGDVSPPP---N------LPAAAASSP-----KETVPAGTSNGLVEADYVVLNTPDGNPRPVGPGGGSR----PSASGP : 193
ORI2_C   : EDDLDNYDGDFTGQSKGKIKGQADTAPS-VKGDVSPPP---N------LPAAAASSP-----KETVPAGTSNGLVEADYVVLNTPDGNPRPVGPGGGSR----PSASGP : 193
CAR8_C   : EDDLDNYDGDFTGQSKGKIKGQADTAPS-VKGDVSPPP---N------LPAAAASSP-----KETVPAGTSNGLVEADYVVLNTPDGNPRPVGPGGGSR----PSASGP : 193
AND7_C   : EDDLDNYDGDFTGQSKGKIKGQADTAPS-VKGDVSPPP---N------LPAAAASSP-----KETVPAGTSNGLVEADYVVLNTPDGNPRPVGPGGGSR----PSASGP : 193
SalI_C   : EDDLDNYDGDFTGQSKGKIKGQADTAPS-VKGDVSPPP---N------LPAAAASSP-----KETVPAGTSNGLVEADYVVLNTPDGNPRPVGPGGGSR----PSASGP : 199
CAR10_C  : EDDLDNYDGDFTGQSKGKIKGQADTAPS-VKGDVSPPP---N------LPAAAASSP-----KETVPAGTSNGLVEADYVVLNTPDGNPRPVGPGGGSR----PSASGP : 193
CAR11_C  : EDDLDNYDGDFTGQSKGKIKGQADTAPS-VKGDVSPPP---N------LPAAAASSP-----KETVPAGTSNGLVEADYVVLNTPDGNPRPVGPGGGSR----PSASGP : 193
AND4_C   : EDDLDNYDGDFTGQSKGKIKGQADTAPS-VKGDVSPPP---N------LPAAAASSP-----KETVPAGTSNGLVEADYVVLNTPDGNPGPVGPGGGSR----PSASGP : 193
AND5_C   : EDDLDNYDGDFTGQSKGKIKGQADTAPS-VKGDVSPPP---N------LPAAAASSP-----KETVPAGTSNGLVEADYVVLNTPDGNPGPVGPGGGSR----PSASGP : 193
CAR9_C   : EDDLDNYDGDFTGQSKGKIKGQADASQS-VKGADTPGS---K------LPAAADSPP-----RGTAADGRNSHVVEIGYINRNSADSSPLAAGSGGDST----LSASGP : 193
AND8_C   : EDDLDNYDGDFTGQSKGKIKGQADASQS-VKGADTPGS---K------LPAAADSPP-----RGTAADGRNSHVVEIGYINRNSADSSPLAAGSGGDST----LSASGP : 193
ORI1_C   : EDDLDNYDGDFTGQSKGKIKGQADASQS-VKGADTPGS---K------LPAAADSPP-----RGTAADGRNSHVVEIGYINRNSADSSPLAAGSGGDST----LSASGP : 193
AND2_C   : EDDLDNYDGDFTGQSKGKIKGQADASQS-VKGADTPGS---K------LPAAADSPP-----RGTAADGRNSHVVEIGYINRNSADSSPLAAGSGGDST----LSASGP : 193
AND3_C   : EDDLDNYDGDFTGQSKGKIKGQADASQS-VKGADTPGS---K------LPAAADSPP-----RGTAADGRNSHVVEIGYINRNSADSSPLAAGSGGDST----LSASGP : 193
CAR15_C  : EDDLDNYDGDFTGQSKGKIKGQADASQS-VKGADTPGS---K------LPAAADSPP-----RGTAADGRNSHVVEIGYINRNSADSSPLAAGSGGDST----LSASGP : 193
CAR2_C   : EDDLDNYDGDFTGQSKGKIKGQADASQS-VKGADTPGS---K------LPAAADSPP-----RGTAADGRNSHVVEIGYINRNSADSSPLAAGSGGDST----LSASGP : 193
CAR4_C   : EDDLDNYDGDFTGQSKGKIKGQADASQS-VKGADTPGS---K------LPAAADSPP-----RGTAADGRNSHVVEIGYINRNSADSSPLAAGSGGDST----LSASGP : 193
CAR7_C   : EDDLDNYDGDFTGQSKGKIKGQADASQS-VKGADTPGS---K------LPAAADSPP-----RGTAADGRNSHVVEIGYINRNSADSSPLAAGSGGDST----LSASGP : 193
CAR6_C   : EDDLDNYDGDFTGQSKGKIKGQADASQS-VKGADTPGS---K------LPAAADSPP-----RGTAADGRNSHVVEIGYINRNSADSSPLAAGSGGDST----LSASGP : 193
CAR5_C   : EDDLDNYDGDFTGQSKGKIKGQADASQS-VKGADTPGS---K------LPAAADSPP-----RGTAADGRNSHVVEIGYINRNSADSSPLAAGSGGDST----LSASGP : 193
ORI3_C   : EDDLDNYDGDFTGQSKGKIKGQADATQS-VKGADTPGS---K------LPAAADSPP-----KGTAADGRNSHVVEIGYINRNSPDSSPLAAGSGGDST----LSASGP : 193
CAR12_C  : EDDLDNYDGDFTGQSKGKIKGQADASQS-VKGADTPGS---K------LPAAADSPP-----RGTAADGRNSHVVEIGYINRNSADSSPLAAGSGGDST----LSASGP : 193
PAC4_C   : EDDLDNYDGDFTGQSKGKIKGQADASQS-VKGADTPGS---K------LPAAADSPP-----RGTAADGRNSHVVEIGYINRNSADSSPLAAGSGGDST----LSASGP : 193
CAR3_C   : EDDLDNYDGDFTGQSKGKIKGQADAAPS-VKGAVTPAS---K------LPAAADSPP-----RGTAADGRNSHVVEIGYINRNSADSSPLAAGSGGDST----LSASGP : 193
PAC8_C   : EDDLDNYDGDFTGQSKGKIKGQADASQS-VKGADTPGS---K------LPAAADSPP-----KETAPEGTNSHVVEIGYINRNSPDSSPLAAGSGGDST----LSASGP : 193
```

```
ORI4_C   : EDDLDNYDGDFTGQSKGKIKGQADASQS-VKGDDTPRS---N------LPAAADSSP-----KETAAAGTSSHVVEIGYVNRNSADSSPRAVGSGGDSR----PSASGP : 193
PAC5_C   : EDDLDNYDGDFTGQSKGKIKGQADAGQS-VKGDVSPRP---N------LPAAADSSP-----KETAPAGTSSGVVEVRYINPNSPDSSPLAAGSGGDST----LSASGP : 193
PAC2_C   : EDDLDNYDADFTGQSKGKIKGQADAGQT-VESNVSPRP---A------SSAADNSLP-----KKTTPAGTSSGVVEVRYVNPNSPD-SPSDALSGGGSR----PSSQGP : 192
PAC6_C   : EDDLDNYDADFTGQSKGKIKGQADAGQT-VESNVSPRP---A------SSAADNSLP-----KKTTPAGTSSGVVEVRYVNPNSPD-SPSDALSGGGSR----PSSQGP : 192
PAC3_C   : EDDLDNYDADFTGQSKGKIKGQADAGQT-VESNVSPRP---A------SSAADNSLP-----KETTPAGTSSGVVEVRYVNPNSPD-SPSDALSGGGSR----PSSQGP : 192
CAR13_C  : EDDLDNYDADFTGQSKGKIKGQADAGQT-VESNVSPRP---A------SSAADNSLP-----KETTPAGTSSGVVEVRYVNPNSPD-SPSDALSGGGSR----PSSQGP : 192
AMA5_C   : EDDLDNYDADFTGQSKGKIKGQADAGQT-VESNVSPRP---A------SSAADNSLP-----KETTPAGTSSGVVEVRYVNPNSPD-SPSDALSGGGSR----PSSQGP : 192
CAR14_C  : EDDLDNYDADFTGQSKGKIKGQADAGQT-VESNVSPRP---A------SSAADNSLP-----KETTPAGTSSGVVEVRYVNPNSPD-SPSDALSGGGSR----PSSQGP : 192
AMA2_C   : EDDLDNYDADFTGQSKGKIKGQADAGQT-VESNVSPRP---A------SSAADNSLP-----KETTPAGTSSGVVEVRYVNPNSPD-SPSDALSGGGSR----PSSQGP : 192
CAR1_C   : EDDLDNYDADFTGQSKGKIKGQADAGQT-VESNVSPRP---A------SSAADNSLP-----KETTPAGTSSGVVEVRYVNPNSPD-SPSDALSGGGSR----PSSQGP : 192
AND1_C   : EDDLDNYDADFTGQSKGKIKGQADAGQT-VESNVSPRP---A------SSAADNSLP-----KETTPAGTSSGVVEVRYVNPNSPD-SPSDALSGGGSR----PSSQGP : 192
AMA4_C   : EDDLDNYDADFTGQSKGKIKGQADAGQT-VESNVSPRP---A------SSAADNSLP-----KKTTPAGTSSGVVEVRYVNPNSPD-SPSDALSGGGSR----PSSQGP : 192
VCGI_C   : EDDLDNYDADFTGQSKGKIKGQADAGQT-VESNVSPRA---A------SSAAGNSLP-----KETTPAGTSSGVVEVRYVNPNSPD-SPSDALSGGGSR----PSSQGP : 192
           EDDLDNYD DF GQ K                                                          s

           20       *       240       *       260       *       280       *       300       *       320
AMA4_H   : SSSSQSTGQSQQSNGAE---------PPAGTTQEVNPNAGQPPPPAGSTG-PAATPGQPESGRVPNVKYLDKLYDEVLKTTDAKDEIHVPPFHSKYNDFRKKYEFTMNE : 296
AND4_H   : SSSSQSTGQSQQSNGAE---------PPAGTTQEVNPNAGQPPPPAGSTG-PAATPGQPESGRVPNVKYLDKLYDEVLKTTDAKDEIHVPPFHSKYNDFRKKYEFTMNE : 296
AND1_H   : SSSSQSTGQSQQSNGAE---------PPAGTTQEVNPNAGQPPPPAGSTG-PAATPGQPESGRVPNVKYLDKLYDEVLKTTDAKDEIHVPPFHSKYNDFRKKYEFTMNE : 296
PAC2_H   : SSSSQSTGQSQQSNGAE---------PPAGTTQEVNTNAGQPPPPAGSTG-PAATPGQPESGRVPNVKYLDKLYDEVLKTTDAKDEIHVPPFHSKYNDFRKKYEFTMNE : 296
PAC3_H   : SSSSQSTGQSQQSNGAE---------PPAGTTQEVNPNTAQTSPPAGSPGGQAATSGQPESGRVPNVKYLDKLYDEVLKTTDAKDEIHVPPFHSKYNDFRKKYEFTMNE : 297
CAR5_H   : SSSSQSTGQSQQSNGAE---------PPAGTTQEVNPNAGQTSPPAGSPGGQAATSGQPESGRVPNVKYLDKLYDEVLKTTDAKDEIHVPPFHSKYNDFRKKYEFTMNE : 302
AMA3_H   : SSSSQSTGQSQPSAGVA---------PSVGNAEAVVTNTEHSSPPAGLPGGQAATSGQPESGRVPNVKYLDKLYDEVLKTTDAKDEIHVPPFHSKYNDFRKKYEFTMNE : 297
AND8_H   : SSSSQSTGQSQPSAGVA---------PSVGNAEAVVTNTEHSSPPAGLPGGQAATSGQPESGRVPNVKYLDKLYDEVLKTTDAKDEIHVPPFHSKYNDFRKKYEFTMNE : 297
AND11_H  : SSSSQSTGGSQPSAGVA---------PSVGNAEAVVTNTEHSSPPAGLPGGQAATPGQPESGRVPNVKYLDKLYDEVLKTTDAKDEIHVPPFHSKYNDFRKKYEFTMNE : 296
AND9_H   : SSSSQSTGGSQPSAGVA---------LPTGTAETVASNTAQTSPPAGLPGGQAATPGQPESGRVPNVKYLDKLYDEVLKTTDAKDEIHVPPFHSKYNDFRKKYEFTMNE : 301
CAR3_H   : SSSSQSTGGSQPSAGVA---------LPTGTAETVASNTAQTSPPAGLPGGQAATPGQPESGRVPNVKYLDKLYDEVLKTTDAKDEIHVPPFHSKYNDFRKKYEFTMNE : 301
CAR6_H   : SSSSQSTGGSQPSAGVA---------LPTGTAETVASNTAQTSPPAGLPGGQAATPGQPESGRVPNVKYLDKLYDEVLKTTDAKDEIHVPPFHSKYNDFRKKYEFTMNE : 301
SalI_H   : PSSSHSTGQPQQSAGVS---------LPTGTAETVASNTAQTSPPAGSPGGQAATSGQPESGRVPNVKYLDKLYDEVLKTTDAKDEIHVPPFHSKYNDFRKKYEFTMNE : 297
AND5_H   : PSSSHSTGQPQQSAGVS---------LPTGTAETVASNTAQTSPPAGSPGGQAATSGQPESGRVPNVKYLDKLYDEVLKTTDAKDEIHVPPFHSKYNDFRKKYEFTMNE : 297
CAR1_H   : PSSSHSTGQPQQSAGVS---------LPTGTAETVASNTAQTSPPAGSPGGQAATSGQPESGRVPNVKYLDKLYDEVLKTTDAKDEIHVPPFHSKYNDFRKKYEFTMNE : 297
CAR2_H   : PSSSHSTGQPQQSAGVS---------LPTGTAETVASNTAQTSPPAGSPGGQAATSGQPESGRVPNVKYLDKLYDEVLKTTDAKDEIHVPPFHSKYNDFRKKYEFTMNE : 297
AND3_H   : PSSGPSTGGSQPSAGVS---------LPTGTAETVASNTAQTSPPAGSPGGQAATSGQPESGRVPNVKYLDKLYDEVLKTTDAKDEIHVPPFHSKYNDFRKKYEFTMNE : 294
CAR8_H   : SSSSHSTGQPQQSAGVS---------LPTGTAETVASNTAQTSPPAGSPGGQAATSGQPESGRVPNVKYLDKLYDEVLKTTDAKDEIHVPPFHSKYNDFRKKYEFTMNE : 300
ORI1_H   : SSSSHSTGQPQQSAGVS---------LPTGTAETVASNTAQTSPPAGSPGGQAATSGQPESGRVPNVKYLDKLYDEVLKTTDAKDEIHVPPFHSKYNDFRKKYEFTMNE : 296
AND10_H  : SSSSHSTGQPQQSAGVS---------LPTGTAETVASNTAQTSPPAGSPGGQAATSGQPESGRVPNVKYLDKLYDEVLKTTDAKDEIHVPPFHSKYNDFRKKYEFTMNE : 299
AND13_H  : SSSSHSTGQPQQSAGVS---------LPTGTAETVASNTAQTSPPAGSPGGQAATSGQPESGRVPNVKYLDKLYDEVLKTTDAKDEIHVPPFHSKYNDFRKKYEFTMNE : 299
CAR11_H  : SSSSHSTGQPQQSAGVS---------LPTGTAETVASNTAQTSPPAGSPGGQAATSGQPESGRVPNVKYLDKLYDEVLKTTDAKDEIHVPPFHSKYNDFRKKYEFTMNE : 299
CAR13_H  : SSSSHSTGQPQQSAGVS---------LPTGTAETVASNTAQTSPPAGSPGGQAATSGQPESGRVPNVKYLDKLYDEVLKTTDAKDEIHVPPFHSKYNDFRKKYEFTMNE : 299
AND12_H  : SSSSHSTGQPQQSAGVS---------LPTGTAETVASNTAQTSPPAGSPGGQAATSGQPESGRVPNVKYLDKLYDEVLKTTDAKDEIHVPPFHSKYNDFRKKYEFTMNE : 299
AND7_H   : SSSSHSTGQPQQSAGVS---------LPTGTAETVASNTAQTSPPAGSPGGQAATSGQPESGRVPNVKYLDKLYDEVLKTTDAKDEIHVPPFHSKYNDFRKKYEFTMNE : 299
AND2_H   : SSSSHSTGQPQQSAGVS---------LPTGTAETVASNTAQTSPPAGSPGGQAATSGQPESGRVPNVKYLDKLYDEVLKTTDAKDEIHVPPFHSKYNDFRKKYEFTMNE : 299
AND14_H  : SSSSHSTGQPQQSAGVS---------LPTGTAETVASNTAQTSPPAGSPGGQAATSGQPESGRVPNVKYLDKLYDEVLKTTDAKDEIHVPPFHSKYNDFRKKYEFTMNE : 296
PAC6_H   : SSSSHSTGQPQQSAGVS---------LPTGTAETVASNTAQTSPPAGSPGGQAATSGQPESGRVPNVKYLDKLYDEVLKTTDAKDEIHVPPFHSKYNDFRKKYEFTMNE : 299
CAR4_H   : SSSSHSTGQPQQSAGVS---------LPTGTAETVASNTAQTSPPAGSPGGQAATSGQPESGRVPNVKYLDKLYDEVLKTTDAKDEIHVPPFHSKYNDFRKKYEFTMNE : 299
VCGI_H   : SSSSPSTGGSQPSAGVS---------LPTGTAETVASNTAQTSPPAGLPGGQAATPGQPESGRVPNVKYLDKLYDEVLKTTDAKDEIHVPPFHSKYNDFRKKYEFTMNE : 299
```

```
PAC1_H   : SSSSPSTGGSQPSAGVS---------LPTGTAETVASNTAQTSPPAGLPGGQAATPGQPESGRVPNVKYLDKLYDEVLKTTDAKDEIHVPPFHSKYNDFRKKYEFTMNE : 299
ORI3_H   : SSSSHSTGQPQQSAGVS---------LPTGTAETVASNTAQTSPPAGSPGGQAATSGQPESGRVPNVKYLDKLYDEVLKTTDAKDEIHVPPFHSKYNDFRKKYEFTMNE : 299
PAC5_H   : SSSSHSTGQPQQSAGVS---------LPTGTAETVASNTAQTSPPAGSPGGQAATSGQPESGRVPNVKYLDKLYDEVLKTTDAKDEIHVPPFHSKYNDFRKKYEFTMNE : 302
PAC7_H   : SSSSHSTGQPQQSAGVS---------LPTGTAETVASNTAQTSPPAGSPGGQAATSGQPESGRVPNVKYLDKLYDEVLKTTDAKDEIHVPPFHSKYNDFRKKYEFTMNE : 299
PAC4_H   : SSSSHSTGQPQQSAGVS---------LPTGTAETVASNTAQTSPPAGSPGGQAATSGQPESGRVPNVKYLDKLYDEVLKTTDAKDEIHVPPFHSKYNDFRKKYEFTMNE : 302
AMA2_H   : SSSGPSTGGSQPSAGVA---------PSVGNAEAVVTNTEHSSPPAGLPGGQAAPPGQPESGRVPNVKYLDKLYDEVLKTTDAKDEIHVPPFHSKYNDFRKKYEFTMNE : 299
AND6_H   : SSSGPSTGGSQPSAGVA---------PSVGNAEAVVTNTEHSSPPAGLPGGQAAPPGQPESGRVPNVKYLDKLYDEVLKTTDAKDEIHVPPFHSKYNDFRKKYEFTMNE : 299
CAR12_H  : SSSGPSTGGSQPSAGVA---------PSVGNAEAVVTNTEHSSPPAGLPGGQAAPPGQPESGRVPNVKYLDKLYDEVLKTTDAKDEIHVPPFHSKYNDFRKKYEFTMNE : 299
CAR4_I   : TSQGPSQTHNPGSSGNA---------EPNSASARTPPAAAEAPLPQPASPDVSTSGEESPNGRVPNVKYLDKLYDEVLKTTDAKDEIHVPPFHSKYNDFRKKYEFTMNE : 295
ORI1_I   : TSQGPSQTHNPGSSGNA---------EPNSASARTPPAAAEAPLPQPASPDVSTSGEESPNGRVPNVKYLDKLYDEVLKTTDAKDEIHVPPFHSKYNDFRKKYEFTMNE : 295
AND7_I   : TSQGPSQTHNPGSSGNA---------EPNSASARTPPAAAEAPLPQPASPDVSTSGEESPNGRVPNVKYLDKLYDEVLKTTDAKDEIHVPPFHSKYNDFRKKYEFTMNE : 295
CAR6_I   : TSQGPSQTHNPGSSGNA---------EPNSASARTPPAAAEAPLPQPASPDVSTSGEESPNGRVPNVKYLDKLYDEVLKTTDAKDEIHVPPFHSKYNDFRKKYEFTMNE : 295
SalI_I   : TSQGPSQTHNPGSSGNA---------EPNSASARTPPAAAEAPLPQPASPDVSTSGEESPNGRVPNVKYLDKLYDEVLKTTDAKDEIHVPPFHSKYNDFRKKYEFTMNE : 301
CAR5_I   : TSQGPSQTHNPGSSGNA---------EPNSASARTPPAAAEAPLPQPASPDVSTSGEESPNGRVPNVKYLDKLYDEVLKTTDAKDEIHVPPFHSKYNDFRKKYEFTMNE : 295
ORI2_I   : TSQGPSQTHNPGSSGNA---------EPNSASARTPPAAAEAPLPQPASPDVSTSGEESPNGRVPNVKYLDKLYDEVLKTTDAKDEIHVPPFHSKYNDFRKGYEFTMNE : 297
CAR2_I   : TSQGPSQTHNPGSSGNA---------EPNSASARTPPAAAEAPLPQPASPDVSTSGEESPNGRVPNVKYLDKLYDEVLKTTDAKDEIHVPPFHSKYNDFRKKYEFTMNE : 295
AND2_I   : TSQGPSQTHNPGSSGNA---------EPNSASARTPPAAAEAPLPQPASPDVSTSGEESPNGRVPNVKYLDKLYDEVLKTTDAKDEIHVPPFHSKYNDFRKKYEFTMNE : 295
PAC3_I   : TSQGPSQTHNPGSSGNA---------EPNSASARTPPAAAEAPLPQPASPDVSTSGEESPNGRVPNVKYLDKLYDEVLKTTDAKDEIHVPPFHSKYNDFRKKYEFTMNE : 295
AND11_I  : TSQGPSQTHNPGSSGNA---------EPNSASARTPPAAAEAPLPQPASPDVSTSGEESPNGRVPNVKYLDKLYDEVLKTTDAKDEIHVPPFHSKYNDFRKKYEFTMNE : 295
PAC6_I   : TSQGPSQTHNPGSSGNA---------EPNSASARTPPAAAEAPLPQPASPDVSTSGEESPNGRVPNVKYLDKLYDEVLKTTDAKDEIHVPPFHSKYNDFRKKYEFTMNE : 295
VCGI_I   : TSQGPSQTHNPGSSGNA---------EPNSASARTPPAAAEAPLPQPASPDVSTSGEESPNGRVPNVKYLDKLYDEVLKTTDAKDEIHVPPFHSKYNDFRKKYEFTMNE : 295
PAC1_I   : TSQGPSQTHNPGSSGNA---------EPNSASARTPPAAAEAPLPQPASPDVSTSGEESPNGRVPNVKYLDKLYDEVLKTTDAKDEIHVPPFHSKYNDFRKKYEFTMNE : 295
ORI3_I   : TSQGPSQTHNPGSSGNA---------EPNSASARTPPAAAEAPLPQPASPDVSTSGEESPNGRVPNVKYLDKLYDEVLKTTDAKDEIHVPPFHSKYNDFRKKYEFTMNE : 295
AND9_I   : TSQGPSQTHNPGSSGNA---------EPNSASARTPPAAAEAPLPQPASPDVSTSGEESPNGRVPNVKYLDKLYDEVLKTTDAKDEIHVPPFHSKYNDFRKKYEFTMNE : 295
CAR3_I   : TSQGPSQTHNPGSSGNA---------EPNSASARTPPAAAEAPLPQPASPDVSTSGEESPNGRVPNVKYLDKLYDEVLKTTDAKDEIHVPPFHSKYNDFRKKYEFTMNE : 295
CAR10_I  : TSQGPSQTHNPGSSGNA---------EPNSASARNPPAAPAAPLPQPASPDVSTSGEESPNGRVPNVKYLDKLYDEVLKTTDAKDEIHVPPFHSKYNDFRKKYEFTMNE : 302
CAR12_I  : TSQGPSQTHTTGSSRNV---------GQSSTSSINPPPAPAAALPQSAPQVVSTPEGEPESGRVPNVKYLDKLYDEVLKTTDAKDEIHVPPFHSKYNDFRKKYEFTMNE : 297
AND6_I   : TSQGPSQTHTTGSSRNV---------GQSSTSSINPPPAPAAALPQSAPQVVSTPEGEPESGRVPNVKYLDKLYDEVLKTTDAKDEIHVPPFHSKYNDFRKKYEFTMNE : 297
AMA5_I   : TSQGPSQTHNPGSSRNE---------GQSSTSSINPPPAPAAALPQSAPQVVSTPEGEPESGRVPNVKYLDKLYDEVLKTTDAKDEIHVPPFHSKYNDFRKKYEFTMNE : 299
AND1_I   : TSQGPSQTHNPGSSRNE---------GQSSTSSINPPPAPAAALPQSAPQVVSTPEGEPESGRVPNVKYLDKLYDEVLKTTDAKDEIHVPPFHSKYNDFRKKYEFTMNE : 297
AND5_I   : TSQGPSQTHNPGSSRNE---------GQSSTSSINPPPAPAAALPQSAPQVVSTPEGEPESGRVPNVKYLDKLYDEVLKTTDAKDEIHVPPFHSKYNDFRKKYEFTMNE : 295
CAR11_I  : TSQGPSQTHNPGSSRNE---------GQSSTSSINPPPAPAAALPQSAPQVVSTPEGEPESGRVPNVKYLDKLYDEVLKTTDAKDEIHVPPFHSKYNDFRKKYEFTMNE : 295
AND12_I  : TSQGPSQTHNPGSSRNE---------GQSSTSSINPPPAPAAALPQSAPQVVSTPEGEPESGRVPNVKYLDKLYDEVLKTTDAKDEIHVPPFHSKYNDFRKKYEFTMNE : 295
AND13_I  : TSQGPSQTHNPGSSRNE---------GQSSTSSINPPPAPAAALPQSAPQVVSTPEGEPESGRVPNVKYLDKLYDEVLKTTDAKDEIHVPPFHSKYNDFRKKYEFTMNE : 295
AND10_I  : TSQGPSQTHNPGSSRNE---------GQSSTSSINPPPAPAAALPQSAPQVVSTPEGEPESGRVPNVKYLDKLYDEVLKTTDAKDEIHVPPFHSKYNDFRKKYEFTMNE : 295
CAR13_I  : TSQGPSQTHNPGSSRNE---------GQSSTSSINPPPAPAAALPQSAPQVVSTPEGEPESGRVPNVKYLDKLYDEVLKTTDAKDEIHVPPFHSKYNDFRKKYEFTMNE : 295
PAC4_I   : TSQEPSQTHTTGSSRNV---------GQSSTSSINPPPAPAAALPQSAPQVVSTPEGEPESGRVPNVKYLDKLYDEVLKTTDAKDEIHVPPFHSKYNDFRKKYEFTMNE : 294
PAC5_I   : TSQEPSQTHTTGSSRNV---------GQSSTSSINPPPAPAAALPQSAPQVVSTPEGEPESGRVPNVKYLDKLYDEVLKTTDAKDEIHVPPFHSKYNDFRKKYEFTMNE : 294
AND3_I   : TSQEPSQTHTTGSSRNE---------GQSSTSSINPPPAPAAALPQSAPQVVSTPEGEPESGRVPNVKYLDKLYDEVLKTTDAKDEIHVPPFHSKYNDFRKKYEFTMNE : 294
AMA3_I   : TSQEPSQTHTTGSSRNE---------GQSSTSSINPPPAPAAALPQSAPQVVSAPEGEPESGRVPNVKYLDKLYDEVLKTTDAKDEIHVPPFHSKYNDFRKGYEFTMNE : 294
ORI4_I   : TSQEPSQTHTTGSSRNV---------GQSSTSSINPPPAPAAALPQSAPQVVSTPEGEPESGRVPNVKYLDKLYDEVLKTTDAKDEIHVPPFHSKYNDFRKKYEFTMNE : 294
CAR1_I   : TSQEPSQTHTTGSSRNV---------GQSSTSSINPPPAPAAALPQSAPQVVSTPEGEPESGRVPNVKYLDKLYDEVLKTTDAKDEIHVPPFHSKYNDFRKKYEFTMNE : 294
AND8_I   : TSQEPSQTHTTGSSRNV---------GQSSTSSINPPPAPAAALPQSAPQVVSTPEGEPESGRVPNVKYLDKLYDEVLKTTDAKDEIHVPPFHSKYNDFRKKYEFTMNE : 294
ORI5_I   : TSQEPSQTHTTGSSRNE---------GQSSTSSINPPPAPAAALPQSAPQVVSTPEGEPESGRVPNVKYLDKLYDEVLKTTDAKDEIHVPPFHSKYNDFRKKYEFTMNE : 294
CAR8_I   : TSQEPSQTHTTGSSRNE---------GQSSTSSINPPPAPAAALPQSAPQVVSTPEGEPESGRVPNVKYLDKLYDEVLKTTDAKDEIHVPPFHSKYNDFRKKYEFTMNE : 294
PAC7_I   : PSQETAPTNPPGPSGNV---------EPNSASARNPPAAAAAALPQPASPVVSAPEGEPESGRVPNVKYLDKLYDEVLKTTDAKDEIHVPPFHSKYNDFRKGYEFTMNE : 294
```

```
PAC2_I   : PSQETAPTNPPGPSGNV---------EPNSASARNPPAAAAAALPQPASPVVSAPEGEPESGRVPNVKYLDKLYDEVLKTTDAKDEIHVPPFHSKYNDFRKGYEFTMNE : 294
AMA4_I   : TSPGLSPTNPSGQSGNV---------GQSSTSSINPPPAPAAALPQSAPQVVSTPEGEPESGRVPNVKYLDKLYDEVLKTTDAKDEIHVPPFHSKYNDFRKGYEFTMNE : 285
AND4_I   : TSPGLSPTNPSGQSGNV---------GQSSTSSINPPPAPAAALPQSAPQVVSTPEGEPESGRVPNVKYLDKLYDEVLKTTDAKDEIHVPPFHSKYNDFRKGYEFTMNE : 285
PAC8_I   : TSPGLSPTNPSGQSGNV---------GQSSTSSINPPPAPAAALPQSAPQVVSTPEGEPESGRVPNVKYLDKLYDEVLKTTDAKDEIHVPPFHSKYNDFRKKYEFTMNE : 285
CAR7_I   : TSPGLSPTNPSGQSGNV---------GQSSTSSINPPPAPAAALPQSAPQVVSTPEGEPESGRVPNVKYLDKLYDEVLKTTDAKDEIHVPPFHSKYNDFRKKYEFTMNE : 285
AND6_C   : DAASNLQNQATAEAGGS-----TNTQGSQ---TGGVSTTPGANQQAGSTP-PGAATAQTEGEREPNVKYLDKLYDDVLATQDSANGIDVPPFHSKYNDFRKKYEFKMND : 293
ORI2_C   : DAASNLQNQATAEAGGS-----TNTQGSQ---TGGVSTTPGANQQAGSTP-PGAATAQTEGEREPNVKYLDKLYDDVLATQDSANGIDVPPFHSKYNDFRKKYEFKMND : 293
CAR8_C   : DAASNLQNQATAEAGGS-----TNTQGSQ---TGGVSTTPGANQQAGSTP-PGAATAQTEGEREPNVKYLDKLYDDVLATQDSANGIDVPPFHSKYNDFRKKYEFKMND : 293
AND7_C   : DAASNLQNQATAEAGGS-----TNTQGSQ---TGGVSTTPGANQQAGSTP-PGAATAQTEGEREPNVKYLDKLYDDVLATQDSANGIDVPPFHSKYNDFRKKYEFKMND : 293
SalI_C   : DAASNLQNQATAEAGGS-----TNTQGSQ---TGGVSTTPGANQQAGSTP-PGAATAQTESEREPNVKYLDKLYDDVLATQDSANGIDVPPFHSKYNDFRKKYEFKMND : 299
CAR10_C  : DAASNLQNQATAEAGGS-----TNTQGSQ---TGGVSTTPGANQQAGSTP-PGASTAQTEGEREPNVKYLDKLYDDVLATQDSANGIDVPPFHSKYNDFRKKYEFKMND : 293
CAR11_C  : DAASNLQNQATAEAGGS-----TNTQGSQ---TGGVSTTPGANQQAGSTP-PGASTAQTEGEREPNVKYLDKLYDDVLATQDSANGIDVPPFHSKYNDFRKKYEFKMND : 293
AND4_C   : DAASNLQNPATAEAGGS-----TNTQGSQ---TGGVSTTPGANQQAGSTP-PGAATAQTEGEREPNVKYLDKLYDDVLATQDSANGIDVPPFHSKYNDFRKKYEFKMND : 293
AND5_C   : DAASNLQNPATAEAGGS-----TNTQGSQ---TGGVSTTPGANQQAGSTP-PGAATAQTEGEREPNVKYLDKLYDDVLATQDSANGIDVPPFHSKYNDFRKKYEFKMND : 293
CAR9_C   : GSASQITQPSPSSPGGVPGNTLTNVQPQTPVGAGGGTNTPGANQQAGSTP-PGAATAQTEGEREPNVKYLDKLYDDVLATQDSANGIDVPPFHSKYNDFRKKYEFKMND : 301
AND8_C   : GSASQITQPSPSSPGGVPGNTLTNVQPQTPVGAGGGTNTPGANQQAGSTP-PGAATAQTEGEREPNVKYLDKLYDDVLATQDSANGIDVPPFHSKYNDFRKKYEFKMND : 301
ORI1_C   : GSASQITQPSPSSPGGVPGNTLTNVQPQTPVGAGGGTNTPGANQQAGSTP-PGAATAQTEGEREPNVKYLDKLYDDVLATQDSANGIDVPPFHSKYNDFRKKYEFKMND : 301
AND2_C   : GSASQITQPSPSSPGGVPGNTLTNVQPQTPVGAGGGTNTPGANQQAGSTP-PGAATAQTEGEREPNVKYLDKLYDDVLATQDSANGIDVPPFHSKYNDFRKKYEFKMND : 301
AND3_C   : GSASQITQPSPSSPGGVPGNTLTNVQPQTPVGAGGGTNTPGANQQAGSTP-PGAATAQTEGEREPNVKYLDKLYDDVLATQDSANGIDVPPFHSKYNDFRKKYEFKMND : 301
CAR15_C  : GSASQITQPSPSSPGGVPGNTLTNVQPQTPVGAGGGTNTPGANQQAGSTP-PGAATAQTEGEREPNVKYLDKLYDDVLATQDSANGIDVPPFHSKYNDFRKKYEFKMND : 301
CAR2_C   : GSASQITQPSPSSPGGVPGNTLTNVQPQTPVGAGGGTNTPGANQQAGSTP-PGAATAQTEGEREPNVKYLDKLYDDVLATQDSANGIDVPPFHSKYNDFRKKYEFKMND : 301
CAR4_C   : GSASQITQPSPSSPGGVPGNTLTNVQPQTPVGAGGGTNTPGANQQAGSTP-PGAATAQTEGEREPNVKYLDKLYDDVLATQDSANGIDVPPFHSKYNDFRKKYEFKMND : 301
CAR7_C   : GSASQITQPSPSSPGGVPGNTLTNVQPQTPVGAGGGTNTPGANQQAGSTP-PGAATAQTEGEREPNVKYLDKLYDDVLATQDSANGIDVPPFHSKYNDFRKKYEFKMND : 301
CAR6_C   : GSASQITQPSPSSPGGVPGNTLTNVQPQTPVGAGGGTNTPGANQQAGSTP-PGAATAQTEGEREPNVKYLDKLYDDVLATQDSANGIDVPPFHSKYNDFRKKYEFKMND : 301
CAR5_C   : GSASQITQPSPSSPGGVPGNTLTNVQPQTPVGAGGGTNTPGANQQAGSTP-PGAATAQTEGEREPNVKYLDKLYDDVLATQDSANGIDVPPFHSKYNDFRKKYEFKMND : 301
ORI3_C   : GSASQITQPSPSSPGGVPGNTLTNVQPQTPVGAGGGTNTPGANQQAGSTP-PGAATAQTEGEREPNVKYLDKLYDDVLATQDSANGIDVPPFHSKYNDFRKKYEFKMND : 301
CAR12_C  : GSASQITQPSPSSPGGVPGNTLTNVQPQTPVGAGGSTNTPGANQQAGSTP-PGAATAQTEGEREPNVKYLDKLYDDVLATQDSANGIDVPPFHSKYNDFRKKYEFKMND : 301
PAC4_C   : GSASQITQPSPSSPGGVPGNTLTNVQPQTPVGAGGGTNTPGANQQAGSTP-PGAATAQTEGEREPNVKYLDKLYDDVLATQDSANGIDVPPFHSKYNDFRKKYEFKMND : 301
CAR3_C   : GSASQITQPSPSSPGGVPGNTLTNVQPQTPVGAGGSTNTPGANQQAGSTP-PGAATAQTEGEREPNVKYLDKLYDDVLATQDSANGIDVPPFHSKYNDFRKKYEFKMND : 301
PAC8_C   : GSASQITQPSPSSPAGVPGNTLTNVQPQTPVGAGGGTNTPGANQQAGSTP-PGAATAQTEGEREPNVKYLDKLYDDVLATQDSANGIDVPPFHSKYNDFRKKYEFKMND : 301
ORI4_C   : GSASQITQPTPSSPGGVPGNTLTNVQPQTPVGAGGGTNTPGANQQAGSTP-PGAATAQTEGEREPNVKYLDKLYDDVLATQDSANGIDVPPFHSKYNDFRKKYEFKMND : 301
PAC5_C   : GSASQITQPSPSSPGGVPGNTLTNVQPQTPVGAGGGTNTPGANQQAGSTP-PGAATAQTEGEREPNVKYLDKLYDDVLATQDSANGIDVPPFHSKYNDFRKKYEFKMND : 301
PAC2_C   : GTSPNVQNQSGNSQAAA--------------EAGGSTNTPGANQQAGSTP-PGAATAQTEGEREPNVKYLDKLYDDVLATQDSANGIDVPPFHSKYNDFRKKYEFKMND : 286
PAC6_C   : GTSPNVQNQSGNSQAAA--------------EAGGSTNTPGANQQAGSTP-PGAATAQTEGEREPNVKYLDKLYDDVLATQDSANGIDVPPFHSKYNDFRKKYEFKMND : 286
PAC3_C   : GTSPNVQNQSGNSQAAA--------------EAGGSTNTPGANQQAGSTP-PGAATAQTEGEREPNVKYLDKLYDDVLATQDSANGIDVPPFHSKYNDFRKKYEFKMND : 286
CAR13_C  : GTSPNVQNQSGNSQAAA--------------EAGGSTNTPGANQQAGSTP-PGAATAQTEGEREPNVKYLDKLYDDVLATQDSANGIDVPPFHSKYNDFRKKYEFKMND : 286
AMA5_C   : GTSPNVQNQSGNSQAAA--------------EAGGSTNTPGANQQAGSTP-PGAATAQTEGEREPNVKYLDKLYDDVLATQDSANGIDVPPFHSKYNDFRKKYEFKMND : 286
CAR14_C  : GTSPNVQNQSGNSQAAA--------------EAGGSTNTPGANQQAGSTP-PGAATAQTEGEREPNVKYLDKLYDDVLATQDSANGIDVPPFHSKYNDFRKKYEFKMND : 286
AMA2_C   : GTSPNVQNQSGNSQAAA--------------EAGGSTNTPGANQQAGSTP-PGAATAQTEGEREPNVKYLDKLYDDVLATQDSANGIDVPPFHSKYNDFRKKYEFKMND : 286
CAR1_C   : GTSPNVQNQSGNSQAAA--------------EAGGSTNTPGANQQAGSTP-PGAATAQTEGEREPNVKYLDKLYDDVLATQDSANGIDVPPFHSKYNDFRKKYEFKMND : 286
AND1_C   : GTSPNVQNQSGNSQAAT--------------EAGGSTNTPGANQQAGSTP-PGAATAQTEGEREPNVKYLDKLYDDVLATQDSANGIDVPPFHSKYNDFRKKYEFKMND : 286
AMA4_C   : GTSPNVQNQSGNSQAAA--------------EAGGSTNTPGANQQAGSTP-PGAATAQTEGEREPNVKYLDKLYDDVLATQDSANGIDVPPFHSKYNDFRKKYEFKMND : 286
VCGI_C   : GTSPNVQNQSGNSQAAA--------------EAGGSTNTPGANQQAGSTP-PGAATAQTEGEREPNVKYLDKLYDDVLATQDSANGIDVPPFHSKYNDFRKKYEFKMND : 286
                        s                                                2 e  R PNVKYLDKLYD VL T D  1 I VPPFHSKYNDFRKkYEF MN

              *       340         *       360         *       380         *       400         *
```

```
AMA4_H   : REYQIVKNLFDAFFKKDGN-PSPADAVSFFKKMLNDPNVQKEFDNFVHGLYGFAKRHNYL-RGERMTDTKLYDKLLKNVVNLLNTIEVK : 383
AND4_H   : REYQIVKNLFDAFFKKDGN-PSPADAVSFFKKMLNDPNVQKEFDNFVHGLYGFAKRHNYL-RGERMTDTKLYDELLKNVVNLLNTIEVK : 383
AND1_H   : REYQIVKNLFDAFFKKDGN-PSPADAVSFFKKMLNDPNVQKEFDNFVHGLYGFAKRHNYL-RGERMTDTKLYDKLLKNVVNLLNTIEVK : 383
PAC2_H   : REYQIVKNLFDAFFKKDGN-PSPADAVSFFKKMLNDPNVQKEFDNFVHGLYGFAKRHNYL-RGERMTDTNLYDKLLKNVVNLLNTIEVK : 383
PAC3_H   : REYQIVKNLFDAFFKKDGN-PSPADAVSFFKKMLNDPNVQKEFDNFVHGLYGFAKRHNYL-RGERMTDTKLYDELLKNVVNLLNTIEVK : 384
CAR5_H   : REYQIVKNLFDAFFKKDGN-PSPADAVSFFKKMLNDPNVQKEFDNFVHGLYGFAKRHNYL-RGERMTDTKLYDELLKNVVNLLNTIEVK : 389
AMA3_H   : REYQIVKNLFDAFFKKDGN-PSPADAVSFFKKMLNDPNVQKEFDNFVHGLYGFAKRHNYL-RGERMTDTKLYDELLKNVVNLLNTIEVK : 384
AND8_H   : REYQIVKNLFDAFFKKDGN-PSPADAVSFFKKMLNDPNVQKEFDNFVHGLYGFAKRHNYL-RGERMTDTKLYDELLKNVVNLLNTIEVK : 384
AND11_H  : REYQIVKNLFDAFFKKDGN-PSPADAVSFFKKMLNDPNVQKEFDNFVHGLYGFAKRHNYL-RGERMTDTKLYDELLKNVVNLLNTIEVK : 383
AND9_H   : REYQIVKNLFDAFFKKDGN-PSPADAVSFFKKMLNDPNVQKEFDNFVHGLYGFAKRHNYL-RGERMTDTKLYDELLKNVVNLLNTIEVK : 388
CAR3_H   : REYQIVKNLFDAFFKKDGN-PSPADAVSFFKKMLNDPNVQKEFDNFVHGLYGFAKRHNYL-RGERMTDTKLYDELLKNVVNLLNTIEVK : 388
CAR6_H   : REYQIVKNLFDAFFKKDGN-PSPADAVSFFKKMLNDPNVQKEFDNFVHGLYGFAKRHNYL-RGERMTDTKLYDELLKNVVNLLNTIEVK : 388
SalI_H   : REYQIVKNLFDAFFKKDGN-PSPADAVSFFKKMLNDPNVQKEFDNFVHGLYGFAKRHNYL-RGERMTDTKLYDELLKNVVNLLNTIEVK : 384
AND5_H   : REYQIVKNLFDAFFKKDGN-PSPADAVSFFKKMLNDPNVQKEFDNFVHGLYGFAKRHNYL-RGERMTDTKLYDELLKNVVNLLNTIEVK : 384
CAR1_H   : REYQIVKNLFDAFFKKDGN-PSPADAVSFFKKMLNDPNVQKEFDNFVHGLYGFAKRHNYL-RGERMTDTKLYDELLKNVVNLLNTIEVK : 384
CAR2_H   : REYQIVKNLFDAFFKKDGN-PSPADAVSFFKKMLNDPNVQKEFDNFVHGLYGFAKRHNYL-RGERMTDTKLYDELLKNVVNLLNTIEVK : 384
AND3_H   : REYQIVKNLFDAFFKKDGN-PSPADAVSFFKKMLNDPNVQKEFDNFVHGLYGFAKRHNYL-RGERMTDTKLYDELLKNVVNLLNTIEVK : 381
CAR8_H   : REYQIVKNLFDAFFKKDGN-PSPADAVSFFKKMLNDPNVQKEFDNFVHGLYGFAKRHNYL-RGERMTDTKLYDKLLKNVVNLLNTIEVK : 387
ORI1_H   : REYQIVKNLFDAFFKKDGN-PSPADAVSFFKKMLNDPNVQKEFDNFVHGLYGFAKRHNYL-RGERMTDTKLYDELLKNVVNLLNTIEVK : 383
AND10_H  : REYQIVKNLFDAFFKKDGN-PSPADAVSFFKKMLNDPNVQKEFDNFVHGLYGFAKRHNYL-RGERMTDTKLYDELLKNVVNLLNTIEVK : 386
AND13_H  : REYQIVKNLFDAFFKKDGN-PSPADAVSFFKKMLNDPNVQKEFDNFVHGLYGFAKRHNYL-RGERMTDTKLYDELLKNVVNLLNTIEVK : 386
CAR11_H  : REYQIVKNLFDAFFKKDGN-PSPADAVSFFKKMLNDPNVQKEFDNFVHGLYGFAKRHNYL-RGERMTDTKLYDELLKNVVNLLNTIEVK : 386
CAR13_H  : REYQIVKNLFDAFFKKDGN-PSPADAVSFFKKMLNDPNVQKEFDNFVHGLYGFAKRHNYL-RGERMTDTKLYDELLKNVVNLLNTIEVK : 386
AND12_H  : REYQIVKNLFDAFFKKDGN-PSPADAVSFFKKMLNDPNVQKEFDNFVHGLYGFAKRHNYL-RGERMTDTKLYDELLKNVVNLLNTIEVK : 386
AND7_H   : REYQIVKNLFDAFFKKDGN-PSPADAVSFFKKMLNDPNVQKEFDNFVHGLYGFAKRHNYL-RGERMTDTKLYDELLKNVVNLLNTIEVK : 386
AND2_H   : REYQIVKNLFDAFFKKDGN-PSPADAVSFFKKMLNDPNVQKEFDNFVHGLYGFAKRHNYL-RGERMTDTKLYDELLKNVVNLLNTIEVK : 386
AND14_H  : REYQIVKNLFDAFFKKDGN-PSPADAVSFFKKMLNDPNVQKEFDNFVHGLYGFAKRHNYL-RGERMTDTKLYDELLKNVVNLLNTIEVK : 383
PAC6_H   : REYQIVKNLFDAFFKKDGN-PSPADAVSFFKKMLNDPNVQKEFDNFVHGLYGFAKRHNYL-RGERMTDTKLYDELLKNVVNLLNTIEVK : 386
CAR4_H   : REYQIVKNLFDAFFKKDGN-PSPADAVSFFKKMLNDPNVQKEFDNFVHGLYGFAKRHNYL-RGERMTDTQLYDELLKNVVNLLNTIEVK : 386
VCGI_H   : REYQIVKNLFGAFFKKDGN-PSPADAVSFFKKMLNDPNVQKEFDNFVHGLYGFAKRHNYL-RGERMTDTQLYDELLKNVVNLLNTIEVK : 386
PAC1_H   : REYQIVKNLFGAFFKKDGN-PSPADAVSFFKKMLNDPNVQKEFDNFVHGLYGFAKRHNYL-RGERMTDTQLYDELLKNVVNLLNTIEVK : 386
ORI3_H   : REYQIVKNLFDAFFKKDGN-PSPADAVSFFKKMLNDPNVQKEFDNFVHGLYGFAKRHNYL-RGERMTDTQLYDELLKNVVNLLNTIEVK : 386
PAC5_H   : REYQIVKNLFDAFFKKDGN-PSPADAVSFFKKMLNDPNVQKEFDNFVHGLYGFAKRHNYL-RGERMTDTQLYDELLKNVVNLLNTIEVK : 389
PAC7_H   : REYQIVKNLFDAFFKKDGN-PSPADAVSFFKKMLNDPNVQKEFDNFVHGLYGFAKRHNYL-RGERMTDTQLYDELLKNVVNLLNTIEVK : 386
PAC4_H   : REYQIVKNLFDAFFKKDGN-PSPADAVSFFKKMLNDPNVQKEFDNFVHGLYGFAKRHNYL-RGERMTDTQLYDELLKNVVNLLNTIEVK : 389
AMA2_H   : REYQIVKNLFDAFFKKDGN-PSPADAVSFFKKMLNDPNVQKEFDNFVHGLYGFAKRHNYL-RGERMTDTNLYDELLKNVVNLLNTIEVK : 386
AND6_H   : REYQIVKNLFDAFFKKDGN-PSPADAVSFFKKMLNDPNVQKEFDNFVHGLYGFAKRHNYL-RGERMTDTNLYDELLKNVVNLLNTIEVK : 386
CAR12_H  : REYQIVKNLFDAFFKKDGN-PSPADAVSFFKKMLNDPNVQKEFDNFVHGLYGFAKRHNYL-RGERMTDTKLYDKLLKNVVNLLNTIEVK : 386
CAR4_I   : REYQIVKNLFDAFFKKDGN-PSPADAVSFFKKMLNDPNVQKEFDNFVHGLYGFAKRHNYL-RDERMNLTDAHKSLFVNTLSLLDTL--- : 379
ORI1_I   : REYQIVKNLFDAFFKKDGN-PSPADAVSFFKKMLNDPNVQKEFDNFVHGLYGFAKRHNYL-RDERMNLTDAHKSLFVNTLSLLDTL--- : 379
AND7_I   : REYQIVKNLFDAFFKKDGN-PSPADAVSFFKKMLNDPNVQKEFDNFVHGLYGFAKRHNYL-RDERMNLTDAHKSLFVNTLSLLDTL--- : 379
CAR6_I   : REYQIVKNLFDAFFKKDGN-PSPADAVSFFKKMLNDPNVQKEFDNFVHGLYGFAKRHNYL-RDERMNLTDAHKSLFVNTLSLLDTL--- : 379
SalI_I   : REYQIVKNLFDAFFKKDGN-PSPADAVSFFKKMLNDPNVQKEFDNFVHGLYGFAKRHNYL-RDERMNLTDAHKSLFVNTLSLLDTL--- : 385
CAR5_I   : REYQIVKNLFDAFFKKDGN-PSPADAVSFFKKMLNDPNVQKEFDNFVHGLYGFAKRHNYL-RDERMNLTDAHKSLFVNTLSLLDTL--- : 379
ORI2_I   : REYQIVKNLFDAFFNKDGN-PSPADAVSFFKKMLNDPNVQKEFDNFVHGLYGFAKRHNYL-RDERMNLTDAHKSLFVNTLSLLDTL--- : 381
CAR2_I   : RECQIVKNLFDACFNKDGN-PSPADAVSFFKKMLNDPNVQKEFDNFVHGLYGFAKRHNYL-RDERMNLTDAHKSLFVNTLSLLDTL--- : 379
```

```
AND2_I  : REYQIVKNLFDAFFKKDGN-PSPADAVSFFKKMLNDPNVQKEFDNFVHGLYGFAKRHNYL-RDERMNLTDAHKSLFVNTLSLLDTL--- : 379
PAC3_I  : REYQIVKNLFDAFFKKDGN-PSPADAVSFFKKMLNDPNVQKEFDNFVHGLYGFAKRHNYL-RDERMNLTDAHKSLFVNTLSLLDTL--- : 379
AND11_I : REYQIVKNLFDAFFKKDGN-PSPADAVSFFKKMLNDPNVQKEFDNFVHGLYGFAKRHNYL-RDERMNLTDAHKSLFVNTLSLLDTL--- : 379
PAC6_I  : REYQIVKNLFDAFFKKDGN-PSPADAVSFFKKMLNDPNVQKEFDNFVHGLYGFAKRHNYL-RDERMNLTDAHKSLFVNTLSLLDTL--- : 379
VCGI_I  : REYQIVKNLFDAFFKKDGN-PSPADAVSFFKKMLNDPNVQKEFDNFVHGLYGFAKRHNYL-RDERMNLTDAHKSLFVNTLSLLDTL--- : 379
PAC1_I  : REYQIVKNLFDAFFKKDGN-PSPADAVSFFKKMLNDPNVQKEFDNFVHGLYGFAKRHNYL-RDERMNLTDAHKSLFVNTLSLLDTL--- : 379
ORI3_I  : REYQIVKNLFDAFFKKDGN-PSPADAVSFFKKMLNDPNVQKEFDNFVHGLYGFAKRHNYL-RGERMNLTDAHKSLFVNTLSLLDTL--- : 379
AND9_I  : REYQIVKNLFDAFFKKDGN-PSPADAVSFFKKMLNDPNVQKEFDNFVHGLYGFAKRHNYL-RGERMNLTDAHKSLFVNTLSLLDTL--- : 379
CAR3_I  : REYQIVKNLFDAFFKKDGN-PSPADAVSFFKKMLNDPNVQKEFDNFVHGLYGFAKRHNYL-RGERMNLTDAHKSLFVNTLSLLDTL--- : 379
CAR10_I : REYQIVKNLFDAFFKKDGN-PSPADAVSFFKKMLNDPNVQKEFDNFVHGLYGFAKRHNYL-RDERMNLTDAHKSLFVNTLSLLDTL--- : 386
CAR12_I : REYQIVKNLFDAFFKKDGN-PSPADAVSFFKKMLNDPNVQKEFDNFVHGLYGFAKRHNYL-RDERMNLTDAHKSLFVNTLSLLDTL--- : 381
AND6_I  : REYQIVKNLFDAFFKKDGN-PSPADAVSFFKKMLNDPNVQKEFDNFVHGLYGFAKRHNYL-RDERMNLTDAHKSLFVNTLSLLDTL--- : 381
AMA5_I  : REYQIVKNLFDAFFKKDGN-PSPADAVSFFKKMLNDPNVQKEFDNFVHGLYGFAKRHNYL-RDERMNLTDAHKSLFVNTLSLLDTL--- : 383
AND1_I  : REYQIVKNLFDAFFKKDGN-PSPADAVSFFKKMLNDPNVQKEFDNFVHGLYGFAKRHNYL-RDERMNLTDAHKSLFVNTLSLLDTL--- : 381
AND5_I  : REYQIVKNLFDAFFKKDGN-PSPADAVSFFKKMLNDPNVQKEFDNFVHGLYGFAKRHNYL-RDERMNLTDAHKSLFVNTLSLLDTL--- : 379
CAR11_I : REYQIVKNLFDAFFNKDGN-PSPADAVSFFKKMLNDPNVQKEFDNFVHGLYGFAKRHNYL-RDERMNLTDAHKSLFVNTLSLLDTL--- : 379
AND12_I : REYQIVKNLFDAFFNKDGN-PSPADAVSFFKKMLNDPNVQKEFDNFVHGLYGFAKRHNYL-RDERMNLTDAHKSLFVNTLSLLDTL--- : 379
AND13_I : REYQIVKNLFDAFFNKDGN-PSPADAVSFFKKMLNDPNVQKEFDNFVHGLYGFAKRHNYL-RDERMNLTDAHKSLFVNTLSLLDTL--- : 379
AND10_I : REYQIVKNLFDAFFNKDGN-PSPADAVSFFKKMLNDPNVQKEFDNFVHGLYGFAKRHNYL-RDERMNLTDAHKSLFVNTLSLLDTL--- : 379
CAR13_I : REYQIVKNLFDAFFNKDGN-PSPADAVSFFKKMLNDPNVQKEFDNFVHGLYGFAKRHNYL-RDERMNLTDAHKSLFVNTLSLLDTL--- : 379
PAC4_I  : REYQIVKNLFDAFFKKDGN-PSPADAVSFFKKMLNDPNVQKEFDNFVHGLYGFAKRHNYL-RDERMNLTDAHKSLFVNTLSLLDTL--- : 378
PAC5_I  : REYQIVKNLFDAFFKKDGN-PSPADAVSFFKKMLNDPNVQKEFDNFVHGLYGFAKRHNYL-RDERMNLTDAHKSLFVNTLSLLDTL--- : 378
AND3_I  : REYQIVKNLFDAFFKKDGN-PSPADAVSFFKKMLNDPNVQKEFDNFVHGLYGFAKRHNYL-RDERMNLTDAHKSLFVNTLSLLDTL--- : 378
AMA3_I  : REYQIVKNLFDAFFNKDGN-PSPADAVSFFKKMLNDPNVQKEFDNFVHGLYGFAKRHNYL-RDERMNLTDAHKSLFVNTLSLLDTL--- : 378
ORI4_I  : REYQIVKNLFDAFFKKDGN-PSPADAVSFFKKMLNDPNVQKEFDNFVHGLYGFAKRHNYL-RDERMNLTDAHKSLFVNTLSLLDTL--- : 378
CAR1_I  : REYQIVKNLFDAFFKKDGN-PSPADAVSFFKKMLNDPNVQKEFDNFVHGLYGFAKRHNYL-RDERMNLTDAHKSLFVNTLSLLDTL--- : 378
AND8_I  : REYQIVKNLFDAFFKKDGN-PSPADAVSFFKKMLNDPNVQKEFDNFVHGLYGFAKRHNYL-RDERMNLTDAHKSLFVNTLSLLDTL--- : 378
ORI5_I  : REYHIVKNLFDAFFNKDGN-PSPADAVSFFKKMLNDPNVQKEFDNFVHGLYGFAKRHNYL-RDERMNLTDAHKSLFVNTLSLLDTL--- : 378
CAR8_I  : REYQIVKNLFDAFFKKDGN-PSPADAVSFFKKMLNDPNVQKEFDNFVHGLYGFAKRHNYL-RGERMNLTDAHKSLFVNTLSLLDTL--- : 378
PAC7_I  : RECQIVKNLFDACFNKDGN-PSPADAVSFFKKMLNDPNVQKEFDNFVHGLYGFAKRHNYL-RDERMNLTDAHKSLFVNTLSLLDTL--- : 378
PAC2_I  : RECQIVKNLFDACFKKDGN-PSPADAVSFFKKMLNDPNVQKEFDNFVHGLYGFAKRHNYL-RDERMNLTDAHKSLFVNTLSLLDTL--- : 378
AMA4_I  : REYQIVKNLFDAFFNKDGN-PSPADAVSFFKKMLNDPNVQKEFDNFVHGLYGFAKRHNYL-RDERMNLTDAHKSLFVNTLSLLDTL--- : 369
AND4_I  : REYQIVKNLFDAFFNKDGN-PSPADAVSFFKKMLNDPNVQKEFDNFVHGLYGFAKRHNYL-RDERMNLTDAHKSLFVNTLSLLDTL--- : 369
PAC8_I  : REYQIVKNLFDAFFKKDGN-PSPADAVSFFKKMLNDPNVQKEFDNFVHGLYGFAKRHNYL-RDERMNLTDAHKSLFVNTLSLLDTL--- : 369
CAR7_I  : REYQIVKNLFDAFFKKDGN-PSPADAVSFFKKMLNDPNVQKEFDNFVHGLYGFAKRHNYL-RGERMNLTDAHKSLFVNTLSLLDTL--- : 369
AND6_C  : SEYKIMKNLFDVSFKKEGQQSSAACLVNVFKKVLDDEHLQKEFDNFVHGFYGFAKRHNYL-RGERMANENLYKDIFKNVVN-------- : 373
ORI2_C  : SEYKIMKNLFDVSFKKEGQQSSAACLVNVFKKVLDDEHLQKEFDNFVHGFYGFAKRHNYL-RGERMANENLYKDIFKNVVN-------- : 373
CAR8_C  : SEYKIMKNLFDVSFKKEGQQSSAACLVNVFKKVLDDEHLQKEFDNFVHGFYGFAKRHNYL-RGERMANENLYKDIFKNVVN-------- : 373
AND7_C  : SEYKIMKNLFDVSFKKEGQQSSAACLVNVFKKVLDDEHLQKEFDNFVHGFYGFAKRHNYL-RGERMANENLYKDIFKNVVN-------- : 373
SalI_C  : SEYKIMKNLFDVSFKKEGQQSSAACLVNVFKKVLDDEHLQKEFDNFVHGFYGFAKRHNYL-RGERMANENLYKDIFKNVVNLLNTIEVV : 387
CAR10_C : SEYKIMKNLFDVSFKKEGQQSSAACLVNVFKKVLDDEHLQKEFDNFVHGFYGFAKRHNYL-RGERMANENLYKDIFKNVVN-------- : 373
CAR11_C : SEYKIMKNLFDVSFKKEGQQSSAACLVNVFKKVLDDEHLQKEFDNFVHGFYGFAKRHNYL-RGERMANENLYKDIFKNVVN-------- : 373
AND4_C  : SEYKIMKNLFDVSFKKEGQQSSAACLVNVFKKVLDDEHLQKEFDNFVHGFYGFAKRHNYL-RGERMANENLYKDIFKNVVN-------- : 373
AND5_C  : SEYKIMKNLFDVSFKKEGQQSSAACLVNVFKKVLDDEHLQKEFDNFVHGFYGFAKRHNYL-RGERMANENLYKDIFKNVVN-------- : 373
CAR9_C  : SEYKIMKNLFDVSFKKEGQQSSAACLVNVFKKVLDDEHLQKEFDNFVHGFYGFAKRHNYL-RGERMANENLYKDIFKNVVN-------- : 381
AND8_C  : SEYKIMKNLFDVSFKKEGQQSSAACLVNVFKKVLDDEHLQKEFDNFVHGFYGFAKRHNYL-RGERMANENLYKDIFKNVVN-------- : 381
```

```
ORI1_C  : SEYKIMKNLFDVSFKKEGQQSSAACLVNVFKKVLDDEHLQKEFDNFVHGFYGFAKRHNYL-RGERMANENLYKDIFKNVVN-------- : 381
AND2_C  : SEYKIMKNLFDVSFKKEGQQSSAACLVNVFKKVLDDEHLQKEFDNFVHGFYGFAKRHNYL-RGERMANENLYKDIFKNVVN-------- : 381
AND3_C  : SEYKIMKNLFDVSFKKEGQQSSAACLVNVFKKVLDDEHLQKEFDNFVHGFYGFAKRHNYL-RGERMANENLYKDIFKNVVN-------- : 381
CAR15_C : SEYKIMKNLFDVSFKKEGQQSSAACLVNVFKKVLDDEHLQKEFDNFVHGFYGFAKRHNYL-RGERMANENLYKDIFKNVVN-------- : 381
CAR2_C  : SEYKIMKNLFDVSFKKEGQQSSAACLVNVFKKVLDDEHLQKEFDNFVHGFYGFAKRHNYL-RGERMANENLYKDIFKNVVN-------- : 381
CAR4_C  : SEYKIMKNLFDVSFKKEGQQSSAACLVNVFKKVLDDEHLQKEFDNFVHGFYGFAKRHNYL-RGERMANENLYKDIFKNVVN-------- : 381
CAR7_C  : SEYKIMKNLFDVSFKKEGQQSSAACLVNVFKKVLDDEHLQKEFDNFVHGFYGFAKRHNYL-RGERMANENLYKDIFKNVVN-------- : 381
CAR6_C  : SEYKIMKNLFDVSFKKEGQQSSAACLVNVFKKVLDDEHLQKEFDNFVHGFYGFAKRHNYL-RGERMANENLYKDIFKNVVN-------- : 381
CAR5_C  : SEYKIMKNLFDVSFKKEGQQSSAACLVNVFKKVLDDEHLQKEFDNFVHGFYGFAKRHNYL-RGERMANENLYKDIFKNVVN-------- : 381
ORI3_C  : SEYKIMKNLFDVSFKKEGQQSSAACLVNVFKKVLDDEHLQKEFDNFVHGFYGFAKRHNYL-RGERMANENLYKDIFKNVVN-------- : 381
CAR12_C : SEYKIMKNLFDVSFKKEGQQSSAACLVNVFKKVLDDEHLQKEFDNFVHGFYGFAKRHNYL-RGERMANENLYKDIFKNVVN-------- : 381
PAC4_C  : SEYKIMKNLFDVSFKKEGQQSSAACLVNVFKKVLDDEHLQKEFDNFVHGFYGFAKRHNYL-RGERMANENLYKDIFKNVVN-------- : 381
CAR3_C  : SEYKIMKNLFDVSFKKEGQQSSAACLVNVFKKVLDDEHLQKEFDNFVHGFYGFAKRHNYL-RGERMANENLYKDIFKNVVN-------- : 381
PAC8_C  : SEYKIMKNLFDVSFKKEGQQSSAACLVNVFKKVLDDEHLQKEFDNFVHGFYGFAKRHNYL-RGERMANENLYKDIFKNVVN-------- : 381
ORI4_C  : SEYKIMKNLFDVSFKKEGQQSSAACLVNVFKKVLDDEHLQKEFDNFVHGFYGFAKRHNYL-RGERMANENLYKDIFKNVVN-------- : 381
PAC5_C  : SEYKIMKNLFDVSFKKEGQQSSAACLVNVFKKVLDDEHLQKEFDNFVHGFYGFAKRHNYL-RGERMANENLYKDIFKNVVN-------- : 381
PAC2_C  : SEYKIMKNLFDVSFKKEGQQSSAACLVNVFKKVLDDEHLQKEFDNFVHGFYGFAKRHNYL-RGERMANENLYKDIFKNVVN-------- : 366
PAC6_C  : SEYKIMKNLFDVSFKKEGQQSSAACLVNVFKKVLDDEHLQKEFDNFVHGFYGFAKRHNYL-RGERMANENLYKDIFKNVVN-------- : 366
PAC3_C  : SEYKIMKNLFDVSFKKEGQQSSAACLVNVFKKVLDDEHLQKEFDNFVHGFYGFAKRHNYL-RGERMANENLYKDIFKNVVN-------- : 366
CAR13_C : SEYKIMKNLFDVSFKKEGQQSSAACLVNVFKKVLDDEHLQKEFDNFVHGFYGFAKRHNYL-RGERMANENLYKDIFKNVVN-------- : 366
AMA5_C  : SEYKIMKNLFDVSFKKEGQQSSAACLVNVFKKVLDDEHLQKEFDNFVHGFYGFAKRHNYL-RGERMANENLYKDIFKNVVN-------- : 366
CAR14_C : SEYKIMKNLFDVSFKKEGQQSSAACLVNVFKKVLDDEHLQKEFDNFVHGFYGFAKRHNYL-RGERMANENLYKDIFKNVVN-------- : 366
AMA2_C  : SEYKIMKNLFDVSFKKEGQQSSAACLVNVFKKVLDDEHLQKEFDNFVHGFYGFAKRHNYL-RGERMANENLYKDIFKNVVN-------- : 366
CAR1_C  : SEYKIMKNLFDVSFKKEGQQSSAACLVNVFKKVLDDEHLQKEFDNFVHGFYGFAKRHNYL-RGERMANENLYKDIFKNVVN-------- : 366
AND1_C  : SEYKIMKNLFDVSFKKEGQQSSAACLVNVFKKVLDDEHLQKEFDNFVHGFYGFAKRHNYL-RGERMANENLYKDIFKNVVN-------- : 366
AMA4_C  : SEYKIMKNLFDVSFKKEGQQSSAACLVNVFKKVLDDEHLQKEFDNFVHGFYGFAKRHNYLERGERMANENLYKDIFKNVVN-------- : 367
VCGI_C  : SEYKIMKNLFDVSFKKEGQQSSAACLVNVFKKVLDDEHLQKEFDNFVHGFYGFAKRHNYL-RGERMANENLYKDIFKNVVN-------- : 366
           Ey I6KNLFd  FkK G   S A  V  FKK6L1D  6QKEFDNFVHG YGFAKRHNYL R ERM        6  N 6
```
