# Supplementary material for: Genetic Diversity and Selection in Three Plasmodium vivax Merozoite Surface Protein 7 (Pvmsp-7) Genes in a Colombian Population
Source: PLoS One. 2012 Sep 25;7(9):e45962. doi: 10.1371/journal.pone.0045962 (PMC3458108; doi:10.1371/journal.pone.0045962)
Supplement: Table S1 — Nucleotide diversity for P. vivax antigens. n: number of isolates, π: nucleotide diversity. (PDF) [file pone.0045962.s014.pdf]

Table S1: Nucleotide diversity for *P. vivax* antigens

Gene	isolates	sites	π	Reference
msp-7C	39	1,043	0.0548	This study
msp-3α	35	2,577	0.0496	(MASCORRO *et al.* 2005)
rbp-2	4	8,454	0,0481	(RAYNER *et al.* 2005)
msp-7I	44	1,053	0.0430	This study
msp-1	175	417	0.0451	(FIGTREE *et al.* 2000)
msp-5	22	1,524	0.0375	(GOMEZ *et al.* 2006)
msp-7H	59	1,068	0.0357	This study
ama-1	72	900	0.0130	(THAKUR *et al.* 2008)
dbp	23	1,388	0.0122	(MARTINEZ *et al.* 2004)
trap	39	1,470	0.0059	(PUTAPORNTIP *et al.* 2001)
msp-7K	27	886	0.0022	(GARZON-OSPINA *et al.* 2011)
rbp-1	4	8,499	0.0018	(RAYNER *et al.* 2005)
rap-2	35	1,161	0.0013	(GARZON-OSPINA *et al.* 2010)
msp-4	30	846	0.0011	(MARTINEZ *et al.* 2005)
rap-1	29	2,413	0.0008	(GARZON-OSPINA *et al.* 2010)
msp-7A	29	1,100	0.0002	(GARZON-OSPINA *et al.* 2011)
msp-10	23	1,336	0.0002	(GARZON-OSPINA *et al.* 2011)

n: number of isolates, π: nucleotide diversity.

References

FIGTREE, M., C. J. PASAY, R. SLADE, Q. CHENG, N. CLOONAN *et al.*, 2000 Plasmodium vivax synonymous substitution frequencies, evolution and population structure deduced from diversity in AMA 1 and MSP 1 genes. Mol Biochem Parasitol **108:** 53-66.

GARZON-OSPINA, D., L. ROMERO-MURILLO and M. A. PATARROYO, 2010 Limited genetic polymorphism of the Plasmodium vivax low molecular weight rhoptry protein complex in the Colombian population. Infect Genet Evol **10:** 261-267.

GARZON-OSPINA, D., L. ROMERO-MURILLO, L. F. TOBON and M. A. PATARROYO, 2011 Low genetic polymorphism of merozoite surface proteins 7 and 10 in Colombian Plasmodium vivax isolates. Infect Genet Evol **11:** 528-531.

GOMEZ, A., C. F. SUAREZ, P. MARTINEZ, C. SARAVIA and M. A. PATARROYO, 2006 High polymorphism in Plasmodium vivax merozoite surface protein-5 (MSP5). Parasitology **133:** 661-672.

MARTINEZ, P., C. F. SUAREZ, P. P. CARDENAS and M. A. PATARROYO, 2004 Plasmodium vivax Duffy binding protein: a modular evolutionary proposal. Parasitology **128:** 353-366.

MARTINEZ, P., C. F. SUAREZ, A. GOMEZ, P. P. CARDENAS, J. E. GUERRERO *et al.*, 2005 High level of conservation in Plasmodium vivax merozoite surface protein 4 (PvMSP4). Infect Genet Evol **5:** 354-361.

MASCORRO, C. N., K. ZHAO, B. KHUNTIRAT, J. SATTABONGKOT, G. YAN *et al.*, 2005 Molecular evolution and intragenic recombination of the merozoite surface protein MSP-3alpha from the malaria parasite Plasmodium vivax in Thailand. Parasitology **131:** 25-35.

PUTAPORNTIP, C., S. JONGWUTIWES, T. TIA, M. U. FERREIRA, H. KANBARA *et al.*, 2001 Diversity in the thrombospondin-related adhesive protein gene (TRAP) of Plasmodium vivax. Gene **268:** 97-104.

RAYNER, J. C., T. M. TRAN, V. CORREDOR, C. S. HUBER, J. W. BARNWELL *et al.*, 2005 Dramatic difference in diversity between Plasmodium falciparum and Plasmodium vivax reticulocyte binding-like genes. Am J Trop Med Hyg **72:** 666-674.

THAKUR, A., M. T. ALAM, H. BORA, P. KAUR and Y. D. SHARMA, 2008 Plasmodium vivax: sequence polymorphism and effect of natural selection at apical membrane antigen 1 (PvAMA1) among Indian population. Gene **419:** 35-42.
