# Supplementary material for: Genetic Diversity and Selection in Three Plasmodium vivax Merozoite Surface Protein 7 (Pvmsp-7) Genes in a Colombian Population
Source: PLoS One. 2012 Sep 25;7(9):e45962. doi: 10.1371/journal.pone.0045962 (PMC3458108; doi:10.1371/journal.pone.0045962)
Supplement: Table S2 — Nucleotide diversity (π) values for subpopulations within Colombia. (PDF) [file pone.0045962.s015.pdf]

Table S2: Nucleotide diversity (π) values for subpopulations within Colombia.

n	Gene	Origin	Sites	π
		Amazon		
4	*msp-7C*		1,101	0.0059 (0.002)
4	*msp-7H*		1,146	0.0460 (0.009)
4	*msp-7I*		1,109	0.0658 (0.014)
		Andean		
8	*msp-7C*		1,098	0.0571 (0.009)
14	*msp-7H*		1,137	0.0336 (0.004)
13	*msp-7I*		1,109	0.0392 (0.008)
		Caribbean		
15	*msp-7C*		1,098	0.0506 (0.008)
10	*msp-7H*		1,149	0.0398 (0.006)
12	*msp-7I*		1,109	0.0425 (0.008)
		Orinoco		
4	*msp-7C*		1,119	0.0531 (0.019)
2	*msp-7H*		1,149	0.0252 (0.013)
5	*msp-7I*		1,133	0.0525 (0.014)
		Pacific		
6	*msp-7C*		1,098	0.0525 (0.010)
7	*msp-7H*		1,146	0.0443 (0.010)
8	*msp-7I*		1,109	0.0620 (0.008)
