# Supplementary material for: Genetic Diversity and Selection in Three Plasmodium vivax Merozoite Surface Protein 7 (Pvmsp-7) Genes in a Colombian Population
Source: PLoS One. 2012 Sep 25;7(9):e45962. doi: 10.1371/journal.pone.0045962 (PMC3458108; doi:10.1371/journal.pone.0045962)
Supplement: Table S3 — Nucleotide and amino acid positions within the 5′- end, central region and 3′-end. (PDF) [file pone.0045962.s016.pdf]

Table S3: Nucleotide and amino acid positions within the 5'- end, central region and 3'-end.

	5'-end	central region	3'-end	
msp-7C	1-390	391-717	718-1,191	**Nucleotide**
	1-130	131-239	240-397	**Amino acid**
msp-7H	1-471	472-771	772-1,200	**Nucleotide**
	1-157	158-257	258-400	**Amino acid**
msp-7I	1-525	526-789	790-1,188	**Nucleotide**
	1-175	176-263	264-396	**Amino acid**
