# Supplementary material for: Genetic Diversity and Selection in Three Plasmodium vivax Merozoite Surface Protein 7 (Pvmsp-7) Genes in a Colombian Population
Source: PLoS One. 2012 Sep 25;7(9):e45962. doi: 10.1371/journal.pone.0045962 (PMC3458108; doi:10.1371/journal.pone.0045962)
Supplement: Table S4 — Positively selected sites detected for Pvmsp-7 genes without taking recombination into account. Numbers according to the reference Sal-I protein sequence Pvmsp-7C: XP_001614132.1, Pvmsp-7H: XP_001614137.1 and Pvmsp-7I: XP_001614138.1. (PDF) [file pone.0045962.s017.pdf]

Table S4: Positively selected sites for *Pvmsp-7* genes without taking recombination into account.

	SLAC	FEL	REL	IFEL
msp-7C		135, 145 and 184	135, 145, 146, 152, 155, 160, 161, 162, 167, 172, 179, 184, 185, 186, 187, 194, 201 and 229	147 and 172
msp-7H	206 and 244	169, 171, 176, 179, 180, 183, 206, 207, 214, 222, 224, 225, 236 and 244	169, 171, 179, 183, 206, 207, 209, 214, 222, 224, 225, 230, 236 and 244	76,81, 84, 100,127, 145, 149, 161, 165, 168, 169, 171, 172, 173, 175, 176, 177, 178, 179, 180, 183, 196, 198, 201, 202, 206, 207, 209, 211, 214, 216, 217, 219, 222, 227, 229, 230, 236, 244, 365 and 369
msp-7I		178, 179, 181, 211, 214, 218 and 294		178, 179, 181, 185, 187, 193, 211, 214 and 218

Numbers according to the reference Sal-I protein sequence *Pvmsp-7C*: XP_001614132.1, *Pvmsp-7H*: XP_001614137.1 and *Pvmsp-7I*: XP_001614138.1.
