# Supplementary material for: Genetic Diversity and Selection in Three Plasmodium vivax Merozoite Surface Protein 7 (Pvmsp-7) Genes in a Colombian Population
Source: PLoS One. 2012 Sep 25;7(9):e45962. doi: 10.1371/journal.pone.0045962 (PMC3458108; doi:10.1371/journal.pone.0045962)
Supplement: Table S5 — Negatively selected sites detected for Pvmsp-7 genes without taking recombination into account. Numbers according to the reference Sal-I protein sequence Pvmsp-7C: XP_001614132.1, Pvmsp-7H: XP_001614137.1 and Pvmsp-7I: XP_001614138.1. (PDF) [file pone.0045962.s018.pdf]

Table S5: Negatively selected sites for *Pvmsp-7* genes without taking recombination into account.

	SLAC	FEL	REL	IFEL
msp-7C	52, 71, 96, 103, 112, 125, 247 and 263	38, 52, 57, 65, 70, 71, 96, 97, 102, 103, 112, 122, 123, 125, 195, 225, 230, 247, 248, 254 and 263	38, 52, 57, 65, 70, 71, 96, 97, 102, 103, 112, 123, 195, 225, 230, 247, 248, 254 and 263	52, 71, 96, 97, 103, 112, 125, 195, 230, 247 and 263
msp-7H	25, 37, 44, 47, 69, 73, 75, 97, 101, 105, 129, 144, 146, 182, 204, 255 and 261	25, 37, 38, 44, 47, 56, 57, 69, 73, 75, 79, 85, 88, 91, 97, 101, 105, 106, 111, 122, 124, 129, 144, 146, 147, 182, 204, 253, 255, 261 and 296	25, 37, 38, 44, 47, 69, 73, 75, 79, 88, 91, 97, 101, 105, 106, 129, 144, 146, 147, 182, 204, 253, 255 and 296	25, 37, 38, 44, 47, 53, 57, 69, 73, 75, 79, 88, 97, 101, 105, 106, 129, 144, 146, 147, 182, 204, 253, 255, 261 and 296
msp-7I	14, 85, 111, 112, 127, 257, 265, 292, 302, 307, 358 and 359	14, 37, 47, 85, 87, 100, 111, 112, 127, 228, 257, 265, 292, 302, 307, 358 and 359	14, 37, 38, 47, 85, 87, 100, 111, 112, 127, 132, 153, 154, 155, 172, 191, 198, 212, 228, 234, 237, 246, 251, 257, 265, 292, 302, 307, 358 and 359	14, 37, 47, 85, 87, 100, 111, 112, 127, 153, 154, 228, 257, 265, 292, 302, 307, 358 and 359

Numbers according to the reference Sal-I protein sequence *Pvmsp-7C*: XP_001614132.1, *Pvmsp-7H*: XP_001614137.1 and *Pvmsp-7I*: XP_001614138.1.
