# Supplementary material for: Genetic Diversity and Selection in Three Plasmodium vivax Merozoite Surface Protein 7 (Pvmsp-7) Genes in a Colombian Population
Source: PLoS One. 2012 Sep 25;7(9):e45962. doi: 10.1371/journal.pone.0045962 (PMC3458108; doi:10.1371/journal.pone.0045962)
Supplement: Table S6 — Positively selected sites detected for Pvmsp-7 genes taking recombination into account. Numbers according to the reference Sal-I protein sequence Pvmsp-7C: XP_001614132.1, Pvmsp-7H: XP_001614137.1 and Pvmsp-7I: XP_001614138.1. (PDF) [file pone.0045962.s019.pdf]

Table S6: Positively selected sites detected for *Pvmsp-7* genes taking recombination into account.

	SLAC	FEL	REL	IFEL
msp-7C		145, 184		
msp-7H	244	171,176, 187, 214, 225 and 244	244 and 365	171, 206, 214, 224, 236 and 244
msp-7I	179	178, 179, 181 and 187		178, 179, 181 and 187

Numbers according to the reference Sal-I protein sequence *Pvmsp-7C*: XP_001614132.1, *Pvmsp-7H*: XP_001614137.1 and *Pvmsp-7I*: XP_001614138.1.
