# Supplementary material for: Genetic Diversity and Selection in Three Plasmodium vivax Merozoite Surface Protein 7 (Pvmsp-7) Genes in a Colombian Population
Source: PLoS One. 2012 Sep 25;7(9):e45962. doi: 10.1371/journal.pone.0045962 (PMC3458108; doi:10.1371/journal.pone.0045962)
Supplement: Table S7 — Negatively selected sites detected for Pvmsp-7 genes taking recombination into account. Numbers according to the reference Sal-I protein sequence Pvmsp-7C: XP_001614132.1, Pvmsp-7H: XP_001614137.1 and Pvmsp-7I: XP_001614138.1. (PDF) [file pone.0045962.s020.pdf]

Table S7: Negatively selected sites detected for *Pvmsp-7* genes taking recombination into account.

	SLAC	FEL	REL	IFEL
msp-7C	103, 112 and 125	38, 52, 57, 65, 70, 71, 96, 102, 103, 112, 122, 123, 125, 176, 195, 202, 225, 230, 248 and 254	38, 39, 52, 53, 57, 65, 68, 70, 71, 76, 96, 97, 98, 100, 102, 103, 112, 121, 122, 123, 125, 126, 139, 142, 164, 176, 188, 195, 202, 209, 213, 215, 221, 222, 224, 225, 226, 229, 230, 247, 248, 254 and 263	52, 65, 70, 71, 96, 102, 103, 112, 122, 123, 125, 176, 195, 225 and 230
msp-7H	44, 47, 69, 97, 101, 144 and 146	25, 37, 44, 47, 56, 57, 69, 73, 79, 85, 97,101, 105, 111, 122,124, 129, 144, 146, 147, 204, 255 and 296	25, 44, 47, 69, 97, 101, 129, 144, 146, 147, 204 and 255	44, 47, 53, 69, 97, 101, 129, 144, 146, 147, 204, 255 and 296
msp-7I	14, 85, 257 and 265	14, 37, 47, 85, 87, 111, 112, 127, 257, 265, 302, 358 and 359	14, 85, 87, 112, 257, 265, 302	14, 37, 47, 85, 87, 111, 112, 257, 265, 302, 358 and 359

Numbers according to the reference Sal-I protein sequence *Pvmsp-7C*: XP_001614132.1, *Pvmsp-7H*: XP_001614137.1 and *Pvmsp-7I*: XP_001614138.1.
