# Supplementary material for: Genetic Diversity and Selection in Three Plasmodium vivax Merozoite Surface Protein 7 (Pvmsp-7) Genes in a Colombian Population
Source: PLoS One. 2012 Sep 25;7(9):e45962. doi: 10.1371/journal.pone.0045962 (PMC3458108; doi:10.1371/journal.pone.0045962)
Supplement: Table S8 — Conversion tracks identified by DnaSP and GENECONV, between Pvmsp-7 genes. Nucleotides and amino acids based in alignment of the Fig. S13. (PDF) [file pone.0045962.s021.pdf]

Table S8: Conversion tracks identified by DnaSP and GENECONV, between *Pvmsp-7* genes.

msp-7C/msp-7H	*msp-7C/msp-7I*	*msp-7H/msp-7I*	
114-245	156-171	114-144	**Nucleotide**
38-82	52-57	38-48	**Amino acid**
156-225	838-1085	144-171	**Nucleotide**
52-75	280-362	38-57	**Amino acid**
291-382		240-245	**Nucleotide**
97-128		80-82	**Amino acid**
294-306		254-276	**Nucleotide**
98-102		85-92	**Amino acid**
804-809		291-306	**Nucleotide**
268-270		97-102	**Amino acid**
71-265		1,155-1,169	**Nucleotide**
24-89		385-390	**Amino acid**
1,089-1,180		913-1,090	**Nucleotide**
363-394		305-364	**Amino acid**
		913-1175	**Nucleotide**
		305-392	**Amino acid**

Nucleotides and amino acids based en alignment of the Fig S13.
