# Supplementary material for: Genetic Diversity and Selection in Three Plasmodium vivax Merozoite Surface Protein 7 (Pvmsp-7) Genes in a Colombian Population
Source: PLoS One. 2012 Sep 25;7(9):e45962. doi: 10.1371/journal.pone.0045962 (PMC3458108; doi:10.1371/journal.pone.0045962)
Supplement: Table S9 — Nucleotides and amino acid positions excluded from the analysis. (PDF) [file pone.0045962.s022.pdf]

Table S9: Nucleotides and amino acid positions excluded from the analysis.

	Sites excluded for analysis										
msp-7C	1-17	544-546	616-618	625-627	631-648	655-666	676-684	706-708	1,105-1,107	1,168-1,191	**Nucleotide**
	1-6	182	206	209	211-216	219-222	226-228	236	369	390-397	**Amino acid**
msp-7H	436-450	448-486	568-627	772-774							**Nucleotide**
	146-150	162	190-209	258							**Amino acid**
msp-7I	1-18	421-522	526-537								**Nucleotide**
	1-6	141-174	176-179								**Amino acid**
